# Evolution of the EGFR pathway in Metazoa and its diversification in the planarian *Schmidtea mediterranea*

**DOI:** 10.1038/srep28071

**Published:** 2016-06-21

**Authors:** Sara Barberán, José M. Martín-Durán, Francesc Cebrià

**Affiliations:** 1 Department of Genetics, Faculty of Biology, University of Barcelona and Institute of Biomedicine of the University of Barcelona (IBUB), Av. Diagonal 643, edifici Prevosti, planta 1, 08028 Barcelona, Catalunya, Spain; 2 Sars International Centre for Marine Molecular Biology, University of Bergen, Thormøhlensgate 55, 5008 Bergen, Norway

## Abstract

The EGFR pathway is an essential signaling system in animals, whose core components are the epidermal growth factors (EGF ligands) and their trans-membrane tyrosine kinase receptors (EGFRs). Despite extensive knowledge in classical model organisms, little is known of the composition and function of the EGFR pathway in most animal lineages. Here, we have performed an extensive search for the presence of EGFRs and EGF ligands in representative species of most major animal clades, with special focus on the planarian *Schmidtea mediterranea*. With the exception of placozoans and cnidarians, we found that the EGFR pathway is potentially present in all other analyzed animal groups, and has experienced frequent independent expansions. We further characterized the expression domains of the EGFR/EGF identified in *S. mediterranea*, revealing a wide variety of patterns and localization in almost all planarian tissues. Finally, functional experiments suggest an interaction between one of the previously described receptors, *Smed-egfr-5*, and the newly found ligand *Smed-egf-6*. Our findings provide the most comprehensive overview to date of the EGFR pathway, and indicate that the last common metazoan ancestor had an initial complement of one EGFR and one putative EGF ligand, which was often expanded or lost during animal evolution.

In multicellular organisms, communication between individual cells is essential for the regulation of complex biological processes, such as growth, differentiation, tissue renewal, and cell death. In animals, the receptor tyrosine kinases (RTKs) are well known mediators of intercellular signaling^1^,^2^. In particular, the epidermal growth factor receptor (EGFR) subfamily is the central constituent of a signaling pathway involved in multiple embryonic and adult processes^3^. For instance, it controls the fate and proliferation of multiple cell types in the invertebrate model systems *Caenorhabditis elegans* and *Drosophila melanogaster*^4^,^5^, and exerts a crucial function in the development and morphogenesis of many organs in vertebrate animals, such as the skin^6^, intestine^7^, the central nervous system^8^ and mammary gland^9^, as well as in the progression of prevalent human diseases, such as cancers^3^.

Although constitutive and ligand-independent activations of the EGFR pathway have been described, especially in the context of several cancers^10^, EGFR signaling depends mainly on the binding of a peptide growth factor of the EGF-family to an EGFR. This interaction induces the phosphorylation of the cytoplasmic tyrosine kinase (TK) domain of the EGFR, and the subsequent activation of different intercellular transduction cascades that regulate downstream effector genes^11^. Despite slight differences, all identified EGFRs share conserved structural features that distinguish them from other RTKs. All consist of an extracellular ligand-binding domain containing two or more cysteine rich clusters, a trans-membrane region, and a cytoplasmic domain with tyrosine kinase activity^12^. The nematode *C. elegans* and the fruit fly *D. melanogaster* have one single receptor (*let-23* and *der*, respectively)^13^,^14^, but vertebrates have four EGFR classes: *egfr* (*ErbB-1/her1*), *ErbB-2* (*her2*), *ErbB-3* (*her3*) and *ErbB-4* (*her4*)^15^. With respect to the ligands, all known EGF proteins are synthesized as transmembrane precursors characterized by the presence of an EGF-like domain (in some cases multiple EGF-like domains) in their extracellular region^16^. Cleavage of this membrane precursor in the extracellular milieu releases the active EGF ligand, which contains a conserved cysteine-containing motif that confers binding specificity^17^. While *C. elegans* has only one ligand (*lin-3*)^18^, *D. melanogaster* has four (*spitz*, *vein*, *gurken* and *keren*)^4^,^19^, and vertebrates have an expanded set of 11 EGF ligands: the *epidermal growth factor* (*egf*), *amphiregulin* (*areg*), *epigen*, *epiregulin* (*epr*), *betacellulin* (*btc*), *heparin-binding EGF-like growth factor* (*hb-egf*), *transforming growth factor-α* (*TGFα*) and four neuregulins (*nrg1-4*)^16^,^20^,^21^. Notably, an additional extracellular immunoglobulin-like domain is present in the *D. melanogaster* ligand *vein,* and in the vertebrate ligands *nrg1* and *nrg2*, which also contain an intracellular neuregulin domain^21^,^22^.

In recent years, research on the planarian flatworm *Schmidtea mediterranea* (Platyhelminthes)^23^,^24^,^25^ has clarified the role of the EGFR signaling pathway in the Spiralia (previously referred to as Lophotrochozoa^26^), which together with the Ecdysozoa (e.g. *C. elegans* and *D. melanogaster*) and the Deuterostomia (e.g. vertebrates) form the three major groups of bilaterally symmetrical animals^26^. The planarian *S. mediterranea* is a well-established model in regenerative research, due to its striking ability to regrow any missing body part after injury^27^. Previous studies have identified four EGFRs in *S. mediterranea* (*Smed-egfr-1*, *Smed-egfr-2*, *Smed-egfr-3* and *Smed-egfr-5*), but only one representative in the related parasitic flatworm species *Echinococcus multilocularis* (*EmER*) and *Schistosoma mansoni* (*SER*)^23^,^25^,^28^,^29^. Additionally, one putative EGF ligand (*Smed-epiregulin-1*) and a neuregulin-like ligand (*Smed-nrg-1*) have been identified^30^,^31^. Interestingly, the planarian EGFRs and ligand show a broad spectrum of expression patterns^23^,^25^,^30^ and are functionally implicated in the regeneration and homeostasis of different organs and cell types, such as the pharynx, the excretory and nervous systems, and the eye pigment cells^23^,^25^. Despite this progress in a member of the Spiralia, the presence, composition, and role of the EGFR pathway in most animal lineages –apart from the classical developmental and biomedical model systems–, is virtually unknown. Accordingly, we also know little about the evolution and functional diversification of this important signaling pathway during animal history.

In this study, we use the numerous accessible genomic and transcriptomic datasets to present phylogenetic analyses of the two key EGFR signaling pathway components –EGFRs and EGF ligands– in representatives of most major animal clades, together with the identification and characterization of two new EGFRs and nine new EGF ligands in the planarian *S. mediterranea*. Our findings indicate that *S. mediterranea* has a total of six EGFRs and nine EGF ligands, which exhibit a wide variety of expression patterns. Silencing of the newly identified EGF ligand *Smed-egf-6* by RNA interference phenocopies the previously reported role of the EGFR *Smed-egfr-5*^25^, suggesting a mechanistic connection between them. Altogether, our data permit us to hypothesize that the last common metazoan ancestor had one EGFR and one probable EGF-like ligand, and that the neuregulin-like type of ligands appeared in the last common bilaterian ancestor. Our study indicates that the evolutionary history of the EGFR pathway is characterized by frequent independent expansions of an originally minimal set of one EGFR and one (Metazoa) or two (Bilateria) EGF ligands. These data may help us to understand how this signaling pathway has acquired its broad diversity of physiological roles, as observed in extant animals.

## Results and Discussion

### Distribution and diversification of EGFRs in Metazoa

**Figure 1:**
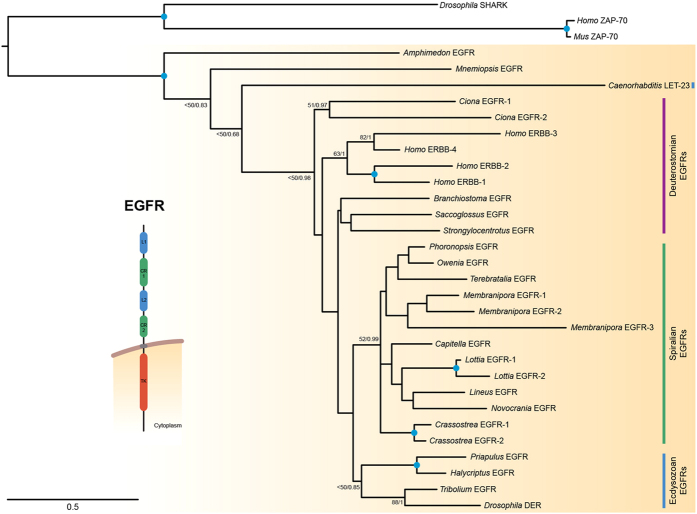
Maximum likelihood (ML) phylogenetic tree of representative EGFRs as obtained by RAxML. The tree was rooted with the EGFR-related tyrosine kinases SHARK and ZAP-70. The model of protein evolution used was LG + G + *I*. Nodal support was obtained by RAxML 1000 replicates (bootstrap value [BV]) and Bayesian posterior probabilities (PP). Both values are shown for key branches. A blue dot at the node indicates BV > 95% and PP > 0.95. Fast evolving sequences, such as those of the planarian *S. mediterranea*, were not included in this analysis (for the entire dataset, see Additional file 1: Figure S1). The archetypical domain architecture of an EGFR is shown on the left.

A thorough investigation of the genomic and transcriptomic data available for a wide diversity of animal lineages revealed that the EGFR subfamily of RTKs is present in almost all animal groups (Table 1), including ctenophores and sponges^32^, the two earliest branching animal clades^26^. Significant exceptions are cnidarians and placozoans, for which the absence of an EGFR ortholog has been previously reported^33^,^34^, and the gastrotrich *Lepidodermella squamata*. In this latter case, further sequencing and sampling efforts will be required to confirm this absence. The orthology assignment of all identified EGFR sequences was confirmed by a phylogenetic analysis of the tyrosine kinase (TK) domain, including representatives of all metazoan RTKs subfamilies and the RTKs of the amoebozoan *Acanthamoeba castellanii* (Supplementary Figure 1). This analysis was corroborated by a further phylogenetic analysis of the whole EGFR sequence (Fig. 1).

Despite particular relationships are not strongly supported in our orthology analysis, the number, distribution (Table 1) and general phylogenetic relationships (Fig. 1) of the EGFRs suggest that a single copy of this RTK was present in the last common metazoan ancestor, as well as at the base of the three major bilaterian groups: the Deuterostomia, the Ecdysozoa and the Spiralia. Subsequent independent expansions occurred in certain bilaterian lineages, such as xenacoelomorphs, tunicates, vertebrates, platyhelminthes, clitellate annelids, molluscs, and bryozoans. While the overall domain architecture of all EGFRs is highly similar (Fig. 1), the number of extracellular cysteine rich domains can vary considerably (Table 1). The previous assumption that vertebrate and invertebrate EGFRs differ in the number of cysteine rich domains (2 in vertebrate EGFRs; 3 in the EGFRs of *D. melanogaster* and *C. elegans*)^35^ is not supported by our data. In fact, the copy number of this extracellular domain seems to oscillate between 2 and 5, and rather than being species-specific, it appears to vary even between paralog EGFRs (Table 1). Interestingly, we found EGFR sequences with potentially inactive (or at least highly divergent) tyrosine kinase domains in the planarian *S. mediterranea* and the limpet *Lottia gigantea* (Table 1) (Supplementary Figure 2). This condition is similar to the situation observed in the human *Erbb3* receptor^36^,^37^,^38^,^39^, and suggests that EGFR duplication in some spiralian lineages has been accompanied by the evolution of alternative regulation/modulation of EGFR signaling.

### The EGFR complement of the planarian *S. mediterranea*

**Figure 2:**
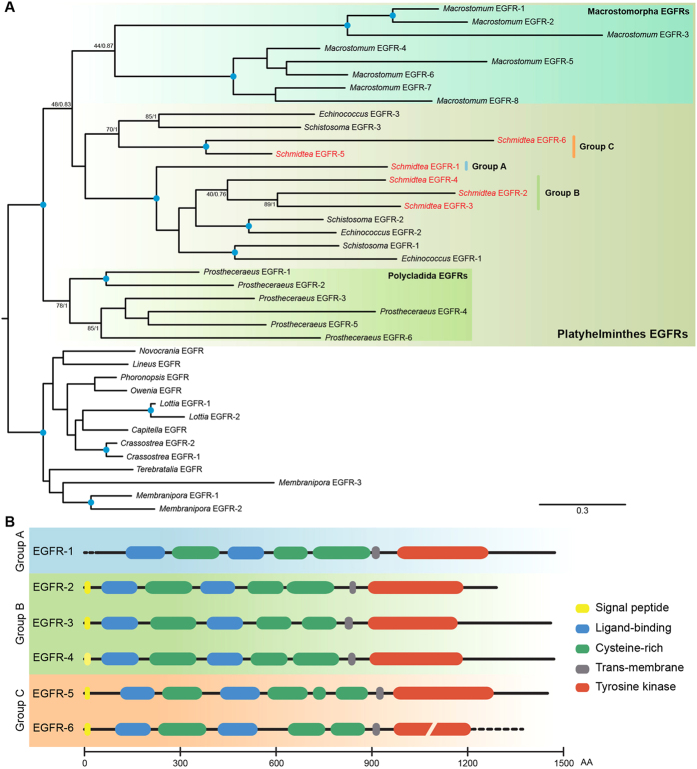
Maximum likelihood (ML) phylogenetic tree of flatworm EGFRs as obtained by RAxML. (**A**) The tree was rooted with the EGFRs of representative spiralian taxa. The model of protein evolution used was LG + G + *I*. Nodal support was obtained by RAxML 550 replicates (bootstrap value [BV]) and Bayesian posterior probabilities (PP). Both values are shown in key branches. A blue dot in the node indicates BV > 95% and PP > 0.95. Planarian sequences are highlighted in red. (**B**) Schematic representation of the domain architecture of each *S. mediterranea* EGFR, drawn to scale.

The re-investigation of the most recent transcriptome and genome resources of *S. mediterranea*^40^,^41^ using the newly identified EGFR sequences of the early branching polyclad flatworm *Prostheceraeus vittatus* (this study) as queries^42^, allowed us to recover two new EGFRs, which we named *Smed-egfr-4* and *Smed-egfr-6*. As such, *S. mediterranea* includes six EGFRs in its genome, which is in the range of the other flatworm species that we analyzed: 8 EGFRs in the macrostomid *Macrostomum lignano*, 6 EGFRs in the polyclad *P. vittatus*, and 3 EGFRs in the parasites *Schistosoma mansoni* and *Echinoccocus multilocularis* (Table 1). An orthology analysis of the recovered flatworm EGFRs using other spiralian sequences as an outgroup (Fig. 2A) revealed that the expansions observed in Macrostomorpha, Polycladida and Neoophora (planarians and parasites) occurred independently, and thus there was likely one single EGFR copy in the last common platyhelminth ancestor, as in most other spiralian lineages. Importantly, planarian EGFRs group in three distinct clades, which we named group A, B, and C (Fig. 2A). The receptor *Smed-egfr-1* is the single representative of group A (Fig. 2B). Group B includes the receptors *Smed-egfr-2*, *Smed-egfr-3* and *Smed-egfr-4*, which exhibit canonical domain architectures, but a varying number of cysteine rich domains (Fig. 2B). Finally, group C consists of the receptors *Smed-egfr-5* and *Smed-egfr-6*, the latter with key amino acid substitutions that may inactivate the ATP binding and active sites of the tyrosine kinase domain (Supplementary Figure 2).

**Figure 3:**
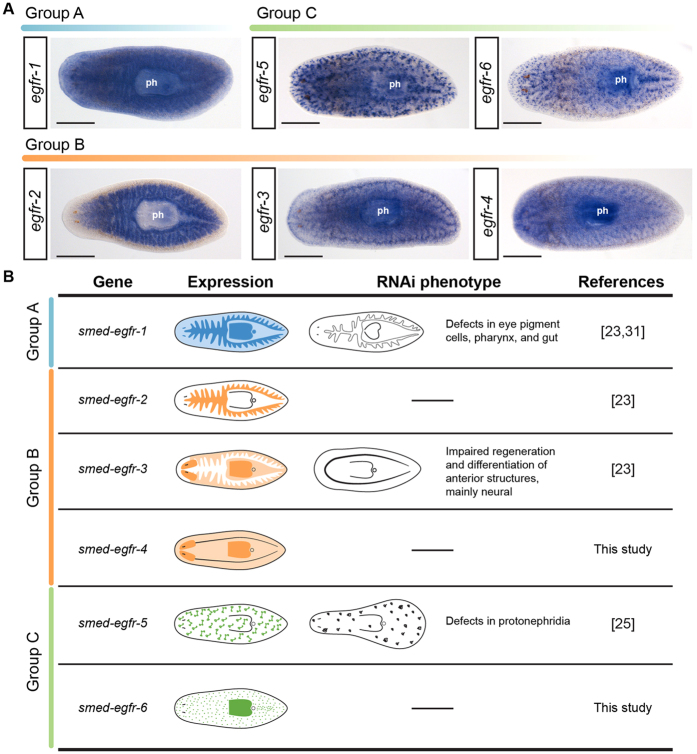
Expression patterns of planarian EGFRs. **(A)** Whole-mount *in situ* hybridizations of all planarian EGFRs on intact adult specimens of *S. mediterranea*, grouped according to their phylogenetic relationship. See main text for details of the expression patterns. **(B)** Schematic summary of the expression patterns of planarian EGFRs and the phenotype observed after their silencing by dsRNA injection, a dash indicates no phenotype was observed (see references for further details). In (**A**), anterior is to the left. ph: pharynx. Scale bars: 500 μm in all panels.

Previous studies characterized the expression and function of the *S. mediterranea* EGFRs *Smed-egfr-1*, *Smed-egfr-2*, *Smed-egfr-3* and *Smed-egfr-5* in adult worms^23^,^25^. The receptor *Smed-egfr-1* is expressed in the gut, pharynx, and eye pigment cells (Fig. 3A), and is required for the proper regeneration and homeostasis of the eyes, pharynx and gut (Fig. 3B)^23^,^31^. The paralog *Smed-egfr-2* is expressed in the gut (Fig. 3A), and no apparent phenotype is observed after silencing^23^. The receptor *Smed-egfr-3* is detected in the neoblast, pharynx and the cephalic ganglia (Fig. 3A) and is required for the proper growth of the blastema during regeneration, probably by regulating cell differentiation (Fig. 3B)^23^,^24^. Finally, *Smed-egfr-5* is expressed in the excretory system (Fig. 3A) and is required for its proper regeneration (Fig. 3B)^25^. We performed whole mount *in situ* hybridization experiments to characterize the expression domains of the newly identified *Smed-egfr-4* and *Smed-egfr-6* genes. The receptor *Smed-egfr-4* was mainly expressed in the central nervous system and pharynx, and weakly in the mesenchyme (Fig. 3A). The paralog *Smed-egfr-6* was expressed in the pharynx and in a discrete pattern throughout the body (Fig. 3A). The silencing by RNAi of either *Smed-egfr-4* or *Smed-egfr-6* gave no perceivable external phenotypes (data not shown). Altogether, our findings support that the expansion of EGFRs that occurred in the lineages leading to *S. mediterranea* has been accompanied by a molecular, transcriptional and functional diversification of the different paralogs. However, further expression and functional analysis in other platyhelminthes and spiralian lineages are essential to better understand the ancestral role of the EGFR in these organisms.

### Distribution of EGF ligands in Metazoa

The evolution of EGF ligands has been mostly studied in vertebrates, *C. elegans*, and *D. melanogaster*^17^,^43^,^44^,^45^. Because the only common feature to all ligands is the presence of an EGF domain, which is a motif present in a tremendous diversity of proteins^17^, we designed a conservative approach to identify new putative EGF ligands. Candidate genes had to be full length (see Material and Methods for the very few exceptions to this criteria), with only one EGF motif, and with domain architectures consistent with the structures of already known EGF ligands^17^. Our *in silico* searches reported 75 new potential EGF ligands (Table 2), in addition to those already described for *Homo sapiens*, *C. elegans*, *D. melanogaster* and *Tribolium castaneum*. Consistent with the absence of an EGFR, we did not recover any putative EGF ligands in the cnidarian databases analyzed. An analysis of domain compositions revealed two basic architectures: the EGF-type of ligands, with only an EGF domain and a varying number of transmembrane regions (from zero, in soluble candidates, to two); and the neuregulin-type (NRG-type) of ligands, which showed an EGF domain combined with an immunoglobulin I-set domain and a transmembrane region (Supplementary Figure 3). Representatives of the first group are the *H. sapiens* EGF ligands *egf*, *hb-egf*, *TGFα*, *areg*, *epr*, *btc*, and *epigen*, *lin-3* from *C. elegans*, and the *D. melanogaster* ligands *gurken*, *keren* and *spitz*. Examples of the NRG-type of EGF ligands are the human *nrg-1* and *nrg-2*, and the *D. melanogaster* gene *vein*. While we were able to recover putative ligands of the EGF-type in almost all animal lineages analyzed –with the exception of the sea urchin *Strongylocentrotus purpuratus* and the phoronid *Phoronopsis harmerii*–, NRG-type ligands were only identified in bilaterian groups (Table 2).

**Figure 4:**
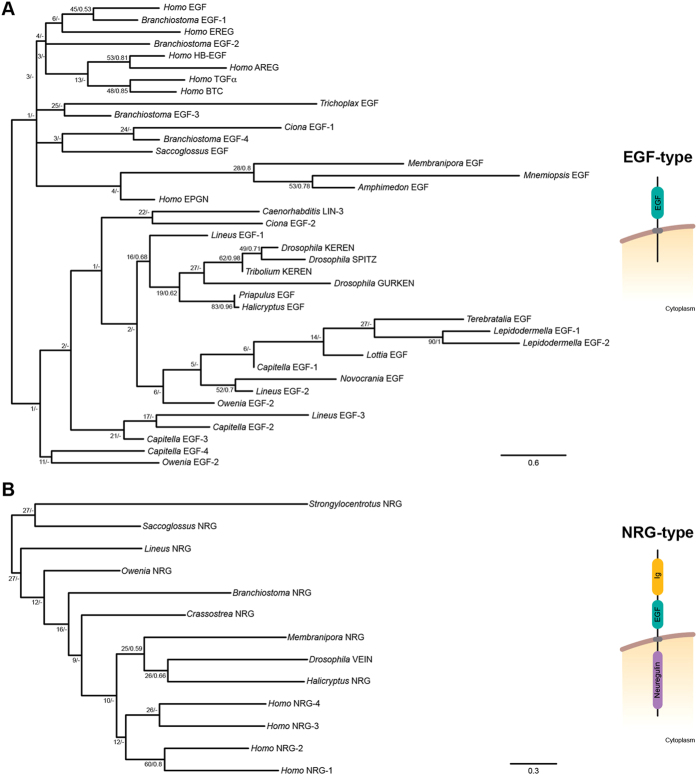
Maximum likelihood (ML) phylogenetic tree of the putative EGF ligands as obtained by RAxML. (**A**) Unrooted tree of the EGF-type of EGF ligands, using the Whelan and Goldman (WAG) + G model of protein evolution. Nodal support was obtained by RAxML 500 replicates (bootstrap value [BV]) and Bayesian posterior probabilities (PP). (**B**) Unrooted tree of the NRG-type of EGF ligands, using the LG + G model of protein evolution. Nodal support was obtained by RAxML 1000 replicates (BV) and Bayesian posterior probabilities (PP). Fast evolving sequences, such as those of the planarian *S. mediterranea*, were not included in these analyses (for the entire dataset, see Additional files 4, 5: Figure S4, S5). The archetypal domain architectures of the EGF-type and NRG-type of ligands are shown on the left.

Phylogenetic studies of the EGF ligands tend to analyze vertebrate and invertebrate sequences separately^43^, to avoid artifacts associated with the low phylogenetic signal obtained from the EGF domain. Therefore, we first performed a sequence-similarity-based clustering analysis of all retrieved putative EGF ligands, with the aim of supporting our domain-based classification (Supplementary Figure 4). NRG-type ligands clustered together, which supported considering them a *bona fide* subtype, but most EGF-type ligands were highly dispersed and weakly interconnected. The failure to recover a well-defined cluster of EGF-type ligands is another sign of the low phylogenetic signal of EGF ligands. This is most likely caused by the extreme variability in the protein structure of these genes, and the fact that the only common region –the EGF motif– is also very variable, beyond the few conserved amino acids that are invariable among all species (e.g. the six conserved cysteines^17^). Following the results of the clustering analysis, we studied the orthology assignments of the two major subtypes of EGF ligands separately (Fig. 4A,B) (Supplementary Figures 5 and 6). In line with previous reports^43^ and our findings after the sequence-similarity analysis, we could not resolve the deepest relationships between all metazoan EGF-type putative ligands. Regarding the NRG-type, we could tentatively assign orthology relationships between the two ambulacrarian NRGs, the two ecdysozoan NRGs, and the four human NRGs, which confirmed a previous analysis of vertebrate EGF ligands^43^. All things considered, the distribution of EGF ligands, the domain architecture of the diversity of candidate genes, and the clustering and phylogenetic analyses, support dividing the EGF ligands into two separate classes: the EGF-type and the NRG-type. The presence of the EGF-type in the ctenophore *Mnemiopsis leidyi*, the sponge *Amphimedon queenslandica* and the placozoan *Trichoplax adhaerens* indicates that this was likely the ancestral EGFR ligand. However, further biochemical analyses are needed to confirm that this putative EGF ligand actually binds the EGFRs present in the ctenophore and the sponge, and thus the existence of a truly functional EGFR signaling pathway in these early branching animal lineages. Consistent with its presence in xenacoelomorphs and most other bilaterian groups, the NRG-type appears to be an innovation of Bilateria.

### The EGF ligand complement of *S. mediterranea*

**Figure 5:**
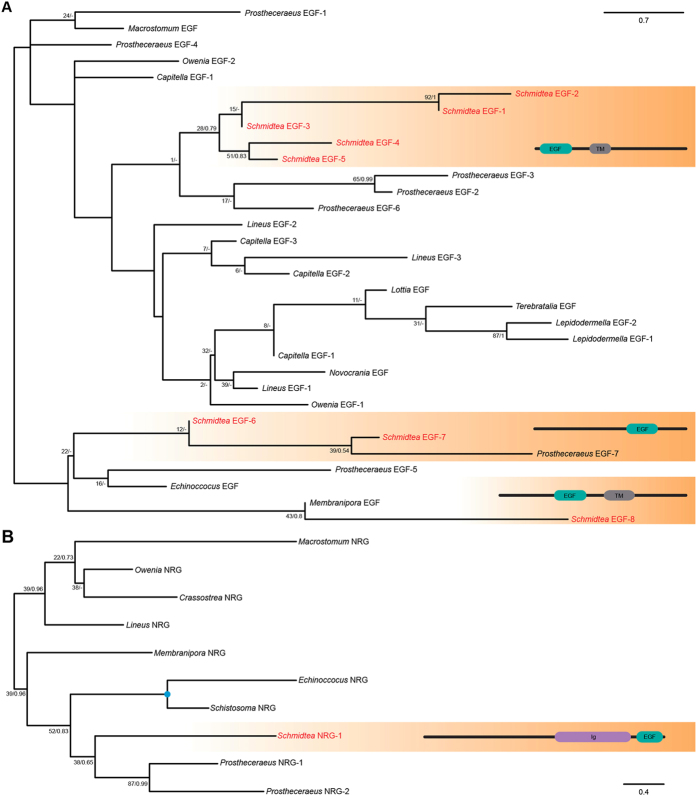
Maximum likelihood (ML) phylogenetic tree of the putative flatworm EGF ligands as obtained by RAxML. (**A**) Unrooted tree of the EGF-type of EGF ligands, using the Whelan and Goldman (WAG) + G model of protein evolution. Nodal support was obtained by RAxML 900 replicates (bootstrap value [BV]) and Bayesian posterior probabilities (PP). (**B**) Unrooted tree of the NRG-type of EGF ligands, using the LG + G model of protein evolution. Nodal support was obtained by RAxML 450 replicates (BV) and Bayesian posterior probabilities (PP). The domain architectures of each subgroup of planarian EGF ligands are shown on the right. Planarian sequences are highlighted in red.

Our search for EGF ligands in the genome^40^ and publicly available transcriptomes of *S. mediterranea*^41^ reported nine candidate genes (Table 2), including the previously described *Smed-epiregulin-1*^30^ and the recently identified *Smed-nrg-1*^31^. Eight of these were of the EGF-type, and only one was of the NRG-type. *Smed-epiregulin-1* belongs to the EGF-type, and given the fact that the gene *epiregulin* is a vertebrate-specific paralog of an ancestral EGF-like gene^44^, we renamed this planarian candidate as *Smed-egf-1*. We named the rest of the planarian putative EGF-type ligands consecutively, from *Smed-egf-2* to *Smed-egf-8*, and the only NRG-type ligand as *Smed-nrg-1*^31^. The phylogenetic analysis of spiralian putative EGF-type ligands did not recover monophyly of the platyhelminth sequences (Fig. 5A), most probably due to the low phylogenetic signal of the EGF domain. However, this analysis suggested three planarian-specific clusters (Fig. 5A). The first group includes from *Smed-egf-1* to *Smed-egf-5*, which are all characterized by presenting the EGF domain upstream of the transmembrane region. The second cluster consists of *Smed-egf-6* and *Smed-egf-7*, and both are secreted ligands without transmembrane domain. Finally, *Smed-egf-8* branches independently from other planarian EGF ligands in our orthology analyses, and has a domain architecture similar to the group of *Smed-egf-1* to *Smed-egf-5*. As observed in the current genomic version^40^, the presence of introns in all the identified ligands and the six EGFRs suggests that the expansion of these two gene families did not occur by retroposition, but rather by gene duplications.

**Figure 6:**
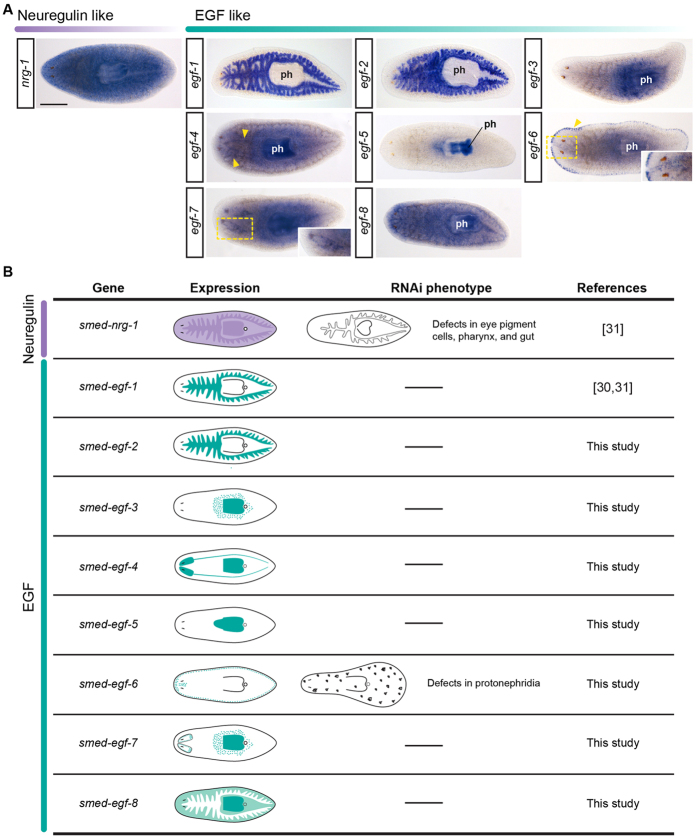
Planarian epidermal growth factors ligands. (**A**) Whole-mount *in situ* hybridizations of all putative planarian EGF ligands on intact adult specimens of *S. mediterranea*, grouped according to their subtype and phylogenetic relationships. See main text for details of the expression patterns. In *egf-4*, the yellow arrowheads indicate the central nervous system. In *egf-6*, the yellow arrowheads indicate expression in the body margin, and the inset (region delimited by the yellow rectangle) is a magnification of the expression in the dorsal anterior midline. Faint signal in the pharynx and central body region in *egf-6* is background staining. In *egf-7*, the inset is a magnification showing the expression in the brain. **(B)** Schematic summary of the expression patterns of the planarian putative EGF ligands and the phenotype observed after their silencing by dsRNA injection (see references and main text for further details). In (**A**), anterior is to the left. ph: pharynx. Scale bars: 500 μm in all panels.

We next analyzed the expression of the identified putative EGF ligands in adult intact specimens of *S. mediterranea* (Fig. 6A). The ligands *Smed-egf-1* and *Smed-egf-2* were expressed in the gut, and *Smed-egf-3* transcripts localized to the pharynx and in scattered mesenchymal cells around it, a pattern reminiscent of other pharynx-related genes, such as *foxA*^46^,^47^. Expression of *Smed-egf-4* was detected in the pharynx, the mesenchyme around it, and the central nervous system (Fig. 6A), while *Smed-egf-5* was expressed in the pharynx and esophagus (Fig. 6A). The putative ligand *Smed-egf-6* was mainly detected in isolated cells of the lateral margin of the planarian, at the boundary between the dorsal and the ventral epidermis. Notably, the expression was stronger and the number of cells greater in the margin of the head, where *Smed-egf-6* also localized to isolated cells of the anterior dorsal midline (Fig. 6A; inset). The EGF-type putative ligand *Smed-egf-7* was expressed in the brain –strongly in the posterior region of the cephalic ganglia–, pharynx and the mesenchyme around it (Fig. 6A), and *Smed-egf-8* was detected in the mesenchyme and pharynx. Finally, *Smed-nrg-1* was expressed in the pharynx and mesenchyme (Fig. 6A). Altogether, these findings show that the nine putative EGF ligands of *S. mediterranea* exhibit a broad diversity of expression patterns, relating mostly to pharyngeal and gut tissues as well as the nervous system (Fig. 6B).

**Figure 7:**
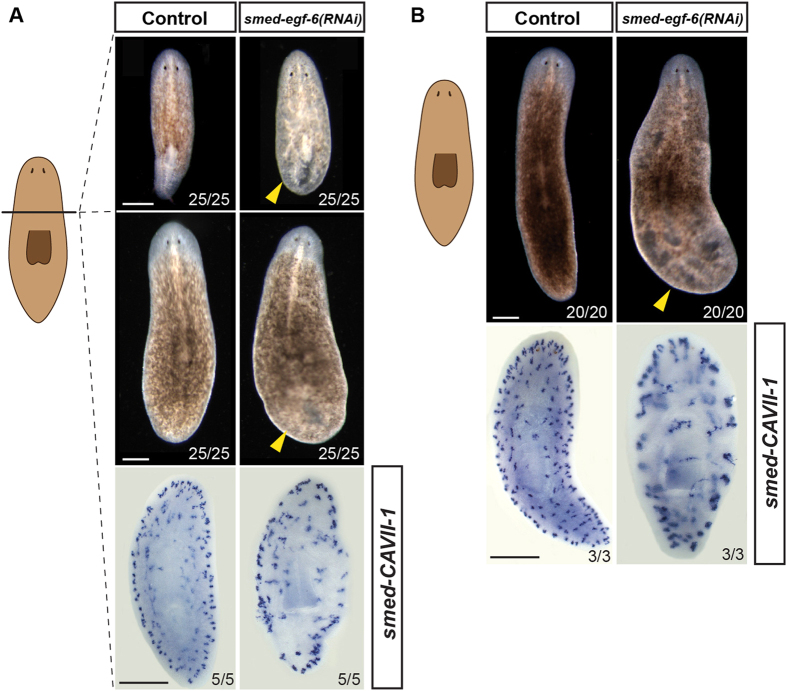
Role of *Smed-egf-6* during adult planarian regeneration and homeostasis. Regenerating (**A**) and intact (**B**) animals were injected on three consecutive days, and fixed two weeks after the last injection. Treated animals form edemas (yellow arrowheads) during both regeneration **(A)** and homeostasis **(B)**. Whole-mount *in situ* hybridization of the protonephridial marker *Smed-CAVII-1* in *Smed-egf-6(RNAi)* animals demonstrates a decrease in the number of protonephridial tubules compared to control animals. Numbers in each panel refer to the frequency of the phenotype. In all panels, anterior is to the top. Scale bars: 500 μm in all panels.

To gain a better understanding of the function of these candidate ligands, and their potential relationships with the described EGFRs, we performed single RNAi gene silencing by double stranded RNA (dsRNA) injections and assessed the consequences during anterior and posterior regeneration. Out of the eight putative EGF-type ligands, we only observed an apparent phenotype after *Smed-egf-6* RNAi, which could indicate some level of functional redundancy between these ligands. However, we cannot discard the possibility that those for which we did not observe any phenotype could be involved in more cell-specific roles that we could not discriminate at the gross morphological level. Silencing of *Smed-egf-6* did not affect the regeneration of missing structures, but rather, produced edemas (Fig. 7A), a phenotype remarkably similar to the one observed after *Smed-egfr-5* RNAi^25^. To determine if the swollen phenotype was caused by defects in the excretory system, as described for *Smed-egfr-5(RNAi)* animals, we analyzed the expression of the specific protonephridial marker carbonic anhydrase (*CAVII-1*)^25^. As expected, *Smed-egf-6(RNAi)* regenerating animals showed a reduced number of, and aberrant, protonephridial tubules (Fig. 7A), suggesting that the edemas were caused by an abnormal regeneration of this organ system. We further confirmed these results during adult homeostasis in intact animals (Fig. 7B). These findings suggest that *Smed-egf-6* could act through *Smed-egfr-5* to regulate planarian excretory system regeneration and homeostasis. We have also recently reported that silencing *Smed-nrg-1* causes an abnormal regeneration of the eyes, gut and pharynx^31^, suggesting that this putative ligand signals via the *Smed-egfr-1* receptor. Although we have only observed an apparent phenotype for two of the nine putative ligands, their roles during regeneration and homeostasis are consistent with those previously assigned to planarian EGFRs. We propose that this concurrence validates our bioinformatic approach for ligand identification.

**Figure 8:**
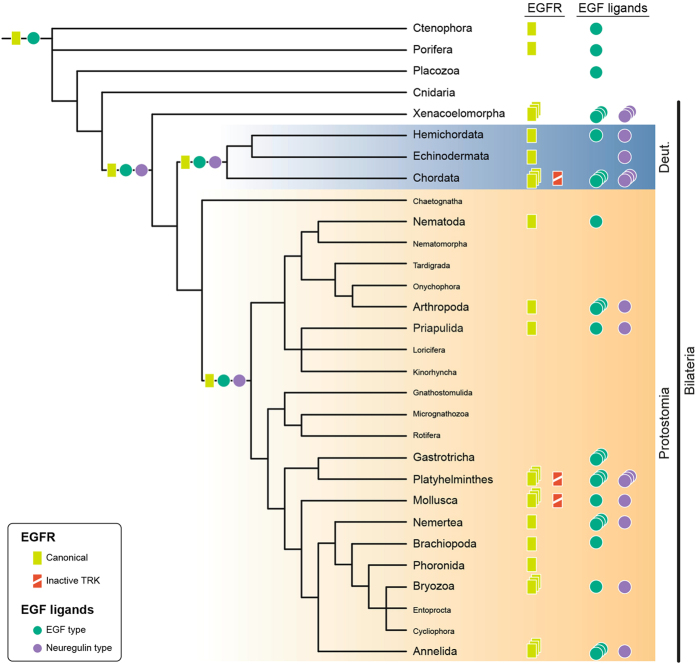
Summary scenario for the evolution of the EGFR signaling pathway in Metazoa. On the right, distribution of the identified EGFRs (yellow rectangles) and putative EGF ligands (EGF-type, green dots; NRG-type, purple dots) in the analyzed animal lineages is shown. A triplicate symbol indicates that this particular type of protein (EGFR, EGF-type and NRG-type of ligands) appears expanded in this lineage. Our findings indicate that the last common metazoan ancestor had one EGFR and one putative EGF-type ligand. The NRG-type of EGF ligand is Bilateria-specific. The ancestral bilaterian set of one EGFR, one EGF-type and one NRG-type of ligands seems to be retained in the last common ancestors of both Deuterostomia and Protostomia. Subsequently, particular bilaterian lineages have experienced expansions of one or more of these basic EGFR signaling components. Additionally, vertebrates, the limpet *L. gigantea* and the planarian *S. mediterranea* have an EGFR with an inactive tyrosine kinase domain, which suggests the independent evolution of alternative regulatory mechanisms of this signaling pathway. Tree topology based on^26^,^59^,^60^.

In summary, the demonstrable relevance of the EGFR pathway in the fields of biomedicine, cancer and developmental biology^3^ contrasts with our limited understanding of the evolution and functional diversification of this pathway in the animal tree of life. Our broad genomic survey of representatives of 19 different major animal clades suggests that the EGFR pathway probably evolved within the metazoan stem lineage (Fig. 8)^32^ and comprised an EGF receptor and a single putative EGF-like ligand, as observed in extant ctenophores and sponges. The EGFR pathway appears to be missing from placozoan and cnidarian lineages, while the presence of a neuregulin-like putative ligand in the vast majority of bilaterian species analyzed indicate that this new type of EGFR ligand appeared together with the Bilateria (Fig. 8). Subsequently, the EGFR and/or the EGF ligands have frequently become expanded in many bilaterian lineages, and new modes of signaling modulation (e.g. via inactive Tyr kinase domains) have appeared (Fig. 8). The planarian *S. mediterranea* (Platyhelminthes) is a prototype of these evolutionary events, because it exhibits an EGFR pathway with 6 EGF receptors –including one putative inactive EGFR–, and 9 EGF ligands –1 neuregulin-like and 8 EGF-like. Gene expression and functional analyses demonstrate that these components are detected in most of the differentiated tissues of the planarian (Figs 3 and 6)^23^,^25^, and control the regeneration and homeostasis of different organs, tissues, and body regions of the animal (Fig. 7), probably by regulating cell differentiation^23^,^24^,^25^,^31^. Altogether, our findings deliver an expanded and detailed evolutionary framework that makes a significant contribution towards our understanding of the molecular and functional diversification of the EGFR pathway in animals, and which will improve future comparisons between emerging biomedical systems, such as planarians, and better-established classical model organisms.

## Methods

### Database searching and phylogenetic analyses

All potential EGFRs and EGF ligands were identified by BLAST and HMMER searches against completed genome/transcriptome databases publicly available or that are being generated in our laboratories (Supplementary Material). Searches were conducted with default parameters and an inclusive E-value of 0.05. For the EGFRs, only sequences including the tyrosine kinase domain (complete or partial) were considered. Regarding the EGF ligands, only full-length sequences with coherent domain architectures were used in subsequent analyses. The only exceptions for this rule were the previously described planarian ligand (*Smed-epiregulin-1*)^30^, which lacks the N-terminal region, as well as the newly identified NRG-type ligand of *X. bockii* and EGF-type ligands of *P. caudatus*, *H. spinulosus* and *S. kowalevskii*, which all lack the very C-terminal end of the protein but give high BLAST and HMMER similarities to other described EGF ligands. The retrieved sequences were aligned using MAFFT v7^48^. Poorly aligned regions of the multiple protein alignment of EGFRs were removed with Gblocks^49^ using the least stringent parameters. Only the EGF domain of the candidate EGF ligands was used for phylogenetic analyses. In the case of the NRG-type ligands, also the immunoglobulin domain was included in the analyses. Maximum likelihood (ML) phylogenetic analyses were conducted with RAxML v8^50^ with the autoMRE option on to calculate the bootstrap support values. Bayesian inference analyses were performed with MrBayes v3^51^ using two parallel runs, sampling every 100 generations. Bayesian posterior probabilities were used for assessing the statistical support of each bipartition.The domain architecture of each identified sequence was analyzed using InterProScan 5^52^ and SignalP 4.1 53, and manually verified when automatic predictions where dubious. The domain information was used to assess the reliability of each sequence of the initial dataset and to help define protein families according to their architectural coherence.

### Clustering analysis of the potential EGF ligands

A sequence-similarity-based (PSI-BLAST *P*-values) clustering approach to analyze the global phylogenetic relationships of the potential EGF ligands was performed with CLANS^54^. A cutting *P*-value of 1e-15 was applied.

### Planarian culture

Asexual *S. mediterranea* from the BCN-10 clonal line were maintained in artificial water^55^. Animals were fed with veal liver and starved for at least 1 week before conducting any experiment.

### Gene cloning and whole-mount *in situ* hybridization

The newly identified planarian EGFRs and putative EGF ligands were isolated by gene-specific PCR and cloned into the pGEM-T vector (Promega). Whole-mount *in situ* hybridization experiments were performed as previously described^56^,^57^. All samples were observed through a Leica MZ16F stereomicroscope and images were captured with a ProgResC3 camera (Jenoptik). Images were processed with Photoshop CS6 (Adobe) and figures were mounted in Illustrator CS6 (Adobe). Brightness/contrast and color balance adjustments were applied to the whole image, not parts.

### RNA interference

Silencing by RNAi was performed as described elsewhere^58^. Control animals were injected with double-stranded RNA for green fluorescent protein (GFP). In *Smed-egf-6(RNAi)* experiments, all animals received one round of injection (each consisting of three injections on consecutive days). For experiments on regenerating animals, planarians were amputated 1 day after the last injection and allowed to regenerate 14 days before fixation. For homeostatic experiments dsRNA-injected animals were kept in starvation for 2 weeks before fixation. In the RNAi experiments of the other components of the EGFR pathway in *S. mediterranea* identified in this study, all animals received two rounds of injections (each consisting of three injections on consecutive days) separated by 3–4 days and were amputated 1 day after both rounds of injection. Since no phenotype was observed during regeneration with these genes, we did not proceed with homeostatic RNAi experiments.

## Additional Information

**How to cite this article**: Barberán, S. *et al.* Evolution of the EGFR pathway in Metazoa and its diversification in the planarian *Schmidtea mediterranea.* *Sci. Rep.* **6**, 28071; doi: 10.1038/srep28071 (2016).

## Supplementary Material

Supplementary Information

## Figures and Tables

**Table 1:** Distribution and diversity of EGFRs in Metazoa.

Clade	Species	No. EGFR	TM^1^	SP	Cys rich	Inactive TK
Ctenophora	*M. leidyi*	1	1	Yes	3	No
Porifera	*A. queenslandica*	1	1	?	1	No
Placozoa	*T. adhaerens*	0	–	–	–	–
Cnidaria	*N. vectensis*	0	–	–	–	–
	*H. magnipapillata*	0	–	–	–	–
Xenacoelomorpha	*X. bocki*	2	1(2)^2^	Yes	2(1), 3(1)	No
	*M. stichopi*	3	1(3)	Yes	2(1), 3(1), 4(1)	No
	*I. pulchra*	5	1(4), ?(1)	Yes(3), ?(2)	1(2), 3(2), ?(1)	No
	*C. macropyga*	3	1(3)	Yes	4(2), 1(1)	No
Priapulida	*P. caudatus*	1	1	?	3	No
	*H. spinulosus*	1	1	Yes	3	No
Nematoda	*C. elegans*	1	1	Yes	3	No
Arthropoda	*T. castaneum*	1	1	Yes	2	No
	*D. melanogaster*	1	1	Yes	3	No
Gastrotricha	*L. squamata*	0	–	–	–	–
Platyhelminthes	*M. lignano*	8	1(6), ?(2)	Yes(5), ?(3)	1(1), 2(2), 3(1), 4(1), ?(3)	No
	*P. vittatus*	6	1(6)	Yes(5), ?(1)	2(2), 3(4)	No
	*S. mediterranea*	6	1(6)	Yes(5), ?(1)	3(4), 4(2)	No(5), Yes(1)
	*S. mansoni*	3	1(3)	Yes(1), ?(2)	3(1), 5(2)	No
	*E. multilocularis*	3	1(3)	Yes(1), ?(2)	3(2), 4(1)	No
Mollusca	*L. gigantea*	2	1(1), ?(1)	Yes(1), ?(1)	2(1), 3(1)	No(1), Yes(1)
	*C. gigas*	2	1(1), ?(1)	?(2)	?	No
Annelida	*O. fusiformis*	1	1	Yes	3	No
	*C. teleta*	1	1	?	3	No
	*H. robusta*	6	1(4), ?(2)	?(6)	2(1), 3(1), 4(2), ?(2)	No
Nemertea	*L. ruber*	1	1	Yes	3	No
Bryozoa	*M. membranacea*	3	1(2), 2(1)	Yes(2), ?(1)	3	No
Brachiopoda	*T. transversa*	1	1	Yes	3	No
	*N. anomala*	1	1	Yes	3	No
Phoronida	*P. harmeri*	1	?	?	?	No
Hemichordata	*S. kowalevskii*	1	1	?	4	No
Echinodermata	*S. purpuratus*	1	1	?	4	No
Chordata	*B. floridae*	1	?	?	?	No
	*C. intestinalis*	2	1	Yes(1), ?(1)	1(1), 3(1)	No
	*H. sapiens*	4	1	Yes	2	No(3), Yes(1)

^1^TM means trans-membrane domains and SP signal peptide.

^2^in parenthesis, number of identified genes exhibiting that number of trans-membrane domains, signal peptide, cysteine-rich domains and inactive/active TK domains.

**Table 2:** Distribution and diversity of EGF ligands in Metazoa.

Clade	Species	No. EGF ligands	EGF-like	Neuregulin-like
Ctenophora	*M. leidyi*	1	1	0
Porifera	*A. queenslandica*	1	1	0
Placozoa	*T. adhaerens*	1	1	0
Cnidaria	*N. vectensis*	0	–	–
	*H. magnipapillata*	0	–	–
Xenacoelomorpha	*X. bocki*	2	1	1
	*M. stichopi*	5	4	1
	*I. pulchra*	5	2	3
	*C. macropyga*	2	0	2
Priapulida	*P. caudatus*	1	1	0
	*H. spinulosus*	2	1	1
Nematoda	*C. elegans*	1	1	0
Arthropoda	*T. castaneum*	1	1	0
	*D. melanogaster*	4	3	1
Gastrotricha	*L. squamata*	2	2	0
Platyhelminthes	*M. lignano*	2	1	1
	*P. vittatus*	9	7	2
	*S. mediterranea*	9	8	1
	*S. mansoni*	1	0	1
	*E. multilocularis*	2	1	1
Mollusca	*L. gigantea*	1	1	0
	*C. gigas*	1	0	1
Annelida	*O. fusiformis*	3	2	1
	*C. teleta*	4	4	0
	*H. robusta*	4	3	1
Nemertea	*L. ruber*	4	3	1
Bryozoa	*M. membranacea*	2	1	1
Brachiopoda	*T. transversa*	1	1	0
	*N. anomala*	1	1	0
Hemichordata	*S. kowalevskii*	2	1	1
Echinodermata	*S. purpuratus*	1	0	1
Chordata	*B. floridae*	5	4	1
	*C. intestinalis*	2	2	0
	*H. sapiens*	11	7	4
