# Supplementary material for: Evolution of the EGFR pathway in Metazoa and its diversification in the planarian Schmidtea mediterranea
Source: Sci Rep. 2016 Jun 21;6:28071. doi: 10.1038/srep28071 (PMC4914847; doi:10.1038/srep28071)
Supplement: Supplementary Information [file srep28071-s1.pdf]

**Supplementary Information**

# Evolution of the EGFR pathway in Metazoa and its diversification in the planarian *Schmidtea mediterranea*

Sara Barberán, José M. Martín-Durán, Francesc Cebrià

SUPPLEMENTARY INFORMATION INDEX


- Supplementary Figure 1–6
- Supplementary Table 1
- Supplementary sequences

**Supplementary Figure Legends**

**Supplementary Figure 1 – Maximum likelihood (ML) phylogenetic tree of all identified EGFRs as obtained by RAxML.** The tree was rooted with the tyrosine kinase (TK) proteins of the amoebozoan *Acanthamoeba castellanii*. The model of protein evolution used was LG + G. Nodal support was obtained by RAxML 300 replicates. This value is only shown for certain branches. Sequences of the same TK subfamily are collapsed. A blue dot at the node indicates bootstrap values above 95%. EGFR sequences are highlighted in orange.

**Supplementary Figure 2 – Multiple sequence alignment of the tyrosine kinase domain of inactive EGFRs.** Alignment of the tyrosine kinase (TK) domain of *C. elegans*, *D. melanogaster*, *H. sapiens*, *S. mediterranea*, *S. mansoni*, *E. multilocularis* and *L. gigantea* to indicate the key amino acid changes (in red) in *H. sapiens* ERBB3, *S. mediterranea* EGFR6 and *L. gigantea* EGFR2 that suggest that their TK domain is inactive. In the case of *L. gigantea*, the ATP binding site domain and part of the TKR domain are missing. This can be a miss-prediction during genome annotation, or alternatively a true inactivation of the TK domain. The identified EGFR2 of *L. gigantea* is not a truncated protein, and includes the extracellular region of the EGFR [Additional files supplementary fasta sequences]. Red asterisks indicate affected positions. The alignment is color-coded based on sequence similarity (dark blue means high conservation, and non-conserved regions are in white).

**Supplementary Figure 3 – Domain architectures of EGF ligands in Metazoa.** Schematic representation of domain architectures for the different EGF ligands retrieved in this study. We only represent each type of architecture, which in some

species is observed in multiple paralogs. As observed, the loss of transmembrane domains has occurred in several lineages (architectures highlighted in red). In the case of NRG-type ligands, the presence of a clear neuregulin domain is only observed in the Deuterostomia and in the priapulid *H. spinulosus*. The asterisk indicates the presence of at least one gene member with a poorly detected signal peptide. In the planarian S. mediterranea, the signal peptide is not well predicted in the ligands *egf-1* to *egf-5*, and with low support in *egf-8*. Drawings are not to scale.

**Supplementary Figure 4 – PSI-BLAST cluster map of putative metazoan EGF ligands.** Nodes are colored based on the type of ligand (EGF-type and NRG-type). *S. mediterranea* ligands are indicated by a double blue circle (EGF-type) and a double green circle (NRG-type). The line between two dots correspond to PSI-BLAST connections with a *P* value < 1e-15.

**Supplementary Figure 5 – Maximum likelihood (ML) phylogenetic tree of all identified EGF-type ligands as obtained by RAxML.** Unrooted tree of the EGF-type of EGF ligands, using the Whelan and Goldman (WAG) + G model of protein evolution. Nodal support was obtained by RAxML 450 replicates.

**Supplementary Figure 6 – Maximum likelihood (ML) phylogenetic tree of all identified NRG-type ligands as obtained by RAxML.** Unrooted tree of the NRG-type of EGF ligands, using the LG + G model of protein evolution. Nodal support was obtained by RAxML 1000 replicates.

**Supplementary Table S1 –ID reference numbers of all proteins used for the phylogenetic analyses carried out in this work.**

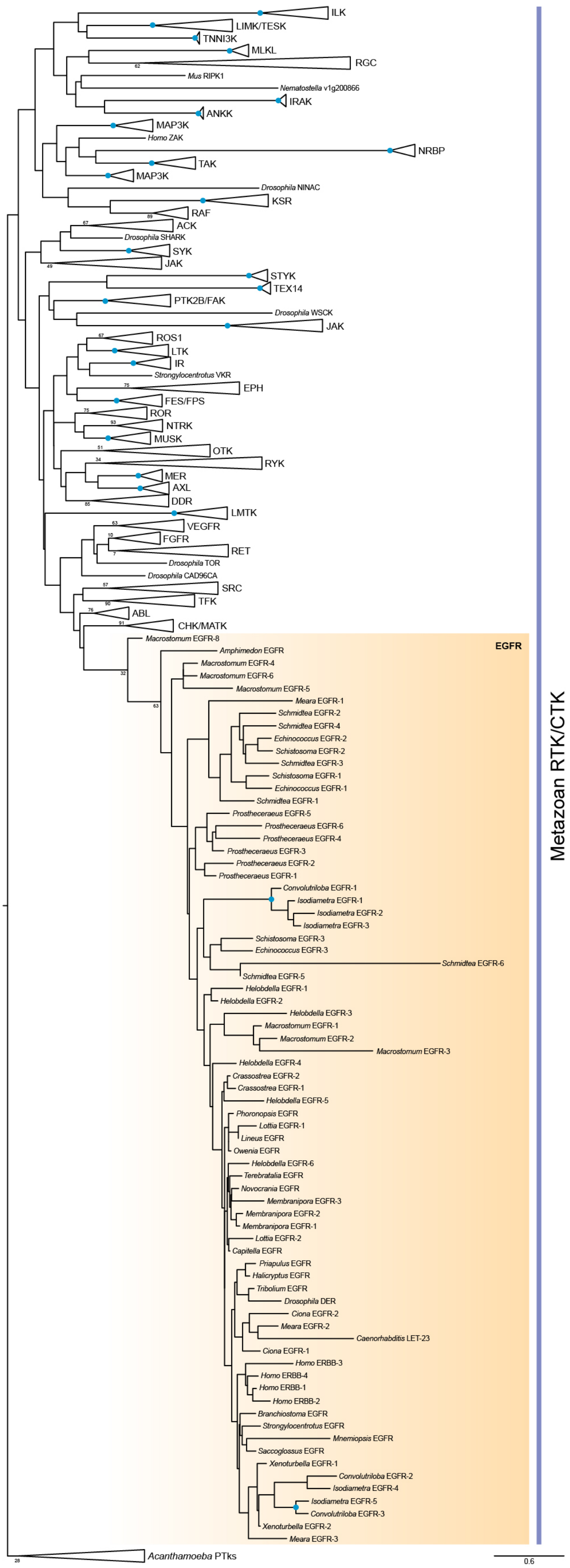
ILK
LIMK/TESK
TNNI3K
MLKL
62
RGC
Mus RIPK1
Nematostella v1g200866
IRAK
ANKK
MAP3K
Homo ZAK
NRBP
TAK
MAP3K
Drosophila NINAC
KSR
89
RAF
67
ACK
Drosophila SHARK
SYK
49
JAK
STYK
TEX14
PTK2B/FAK
Drosophila WSCK
JAK
67
ROS1
LTK
IR
Strongylocentrotus VKR
75
EPH
FES/FPS
75
ROR
93
NTRK
MUSK
51
OTK
34
RYK
MER
AXL
85
DDR
LMTK
63
VEGFR
10
FGFR
7
RET
Drosophila TOR
Drosophila CAD96CA
57
SRC
90
TFK
76
ABL
91
CHK/MATK
EGFR
Macrostomum EGFR-8
32
Amphimedon EGFR
Macrostomum EGFR-4
Macrostomum EGFR-6
63
Macrostomum EGFR-5
Meara EGFR-1
Schmidtea EGFR-2
Schmidtea EGFR-4
Echinococcus EGFR-2
Schistosoma EGFR-2
Schmidtea EGFR-3
Schistosoma EGFR-1
Echinococcus EGFR-1
Schmidtea EGFR-1
Prostheceraeus EGFR-5
Prostheceraeus EGFR-6
Prostheceraeus EGFR-4
Prostheceraeus EGFR-3
Prostheceraeus EGFR-2
Prostheceraeus EGFR-1
Convolutriloba EGFR-1
Isodiametra EGFR-1
Isodiametra EGFR-2
Isodiametra EGFR-3
Schistosoma EGFR-3
Echinococcus EGFR-3
Schmidtea EGFR-6
Schmidtea EGFR-5
Helobdella EGFR-1
Helobdella EGFR-2
Helobdella EGFR-3
Macrostomum EGFR-1
Macrostomum EGFR-2
Macrostomum EGFR-3
Helobdella EGFR-4
Crassostrea EGFR-2
Crassostrea EGFR-1
Helobdella EGFR-5
Phoronopsis EGFR
Lottia EGFR-1
Lineus EGFR
Owenia EGFR
Helobdella EGFR-6
Terebratalia EGFR
Novocrania EGFR
Membranipora EGFR-3
Membranipora EGFR-2
Membranipora EGFR-1
Lottia EGFR-2
Capitella EGFR
Priapulus EGFR
Halicryptus EGFR
Tribolium EGFR
Drosophila DER
Ciona EGFR-2
Meara EGFR-2
Caenorhabditis LET-23
Ciona EGFR-1
Homo ERBB-3
Homo ERBB-4
Homo ERBB-1
Homo ERBB-2
Branchiostoma EGFR
Strongylocentrotus EGFR
Mnemiopsis EGFR
Saccoglossus EGFR
Xenoturbella EGFR-1
Convolutriloba EGFR-2
Isodiametra EGFR-4
Isodiametra EGFR-5
Convolutriloba EGFR-3
Xenoturbella EGFR-2
Meara EGFR-3
28
Acanthamoeba PTKs
0.6
Metazoan RTK/CTK

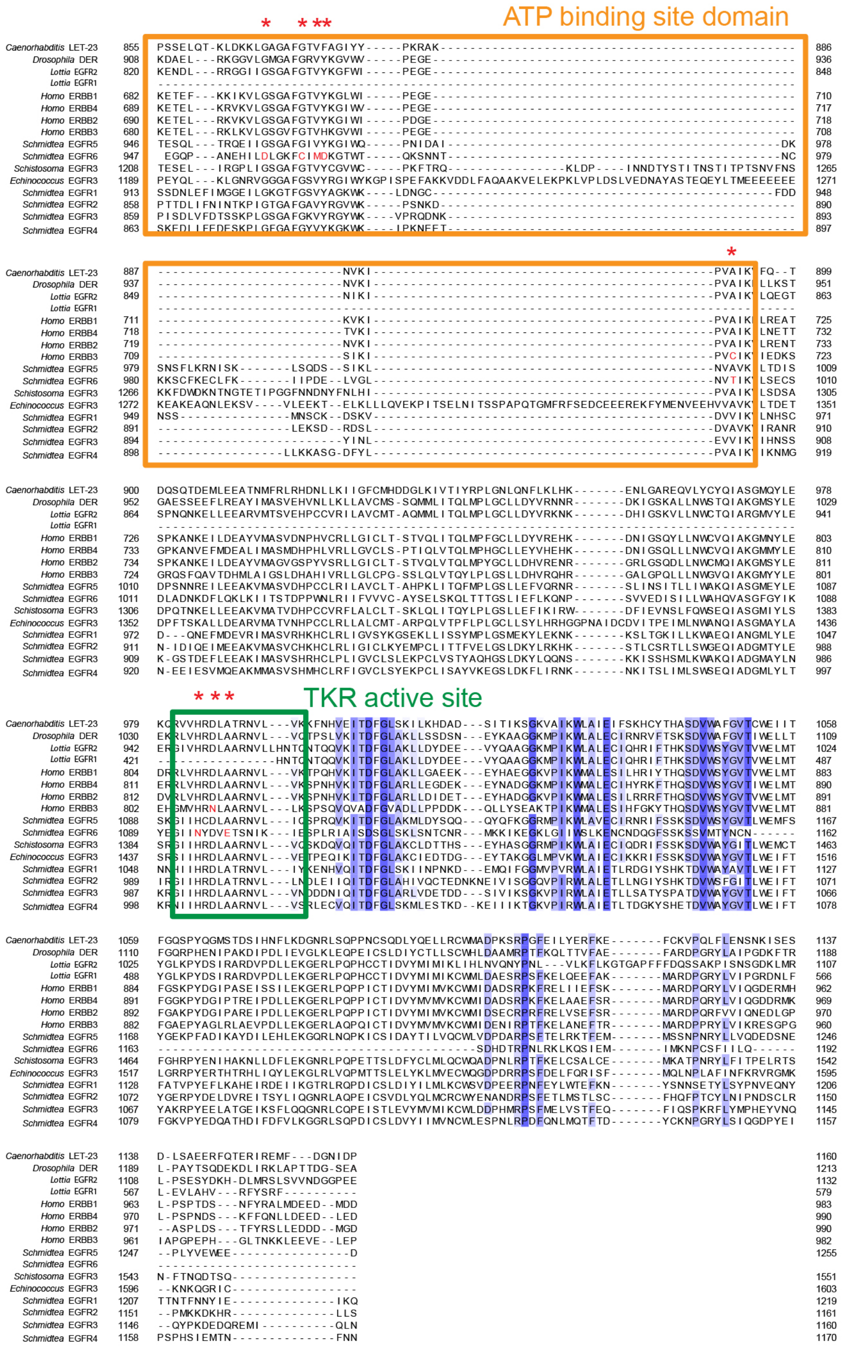
ATP binding site domain
TKR active site

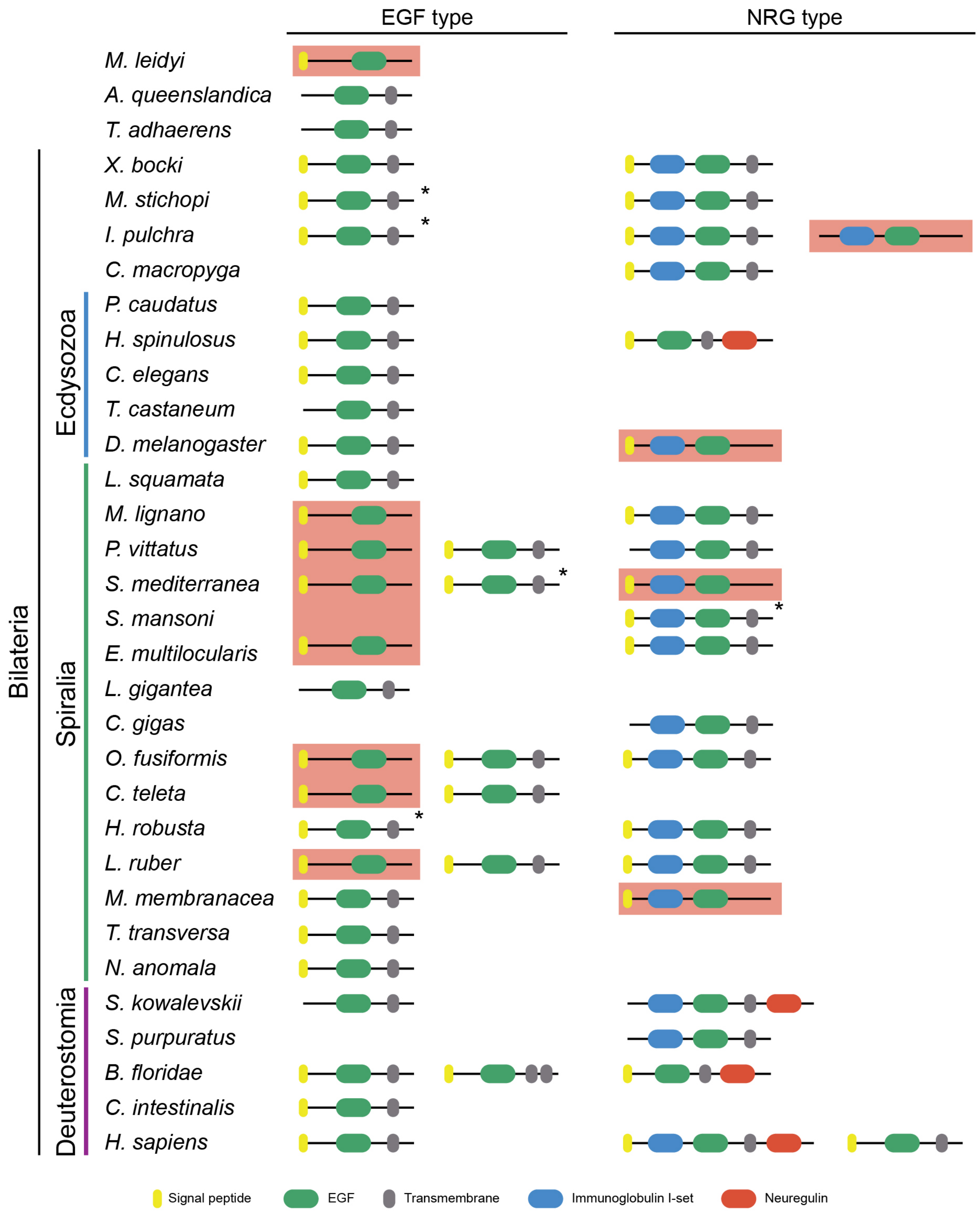
EGF type
NRG type
M. leidyi
A. queenslandica
T. adhaerens
X. bocki
M. stichopi
I. pulchra
C. macropyga
P. caudatus
H. spinulosus
C. elegans
T. castaneum
D. melanogaster
L. squamata
M. lignano
P. vittatus
S. mediterranea
S. mansoni
E. multilocularis
L. gigantea
C. gigas
O. fusiformis
C. teleta
H. robusta
L. ruber
M. membranacea
T. transversa
N. anomala
S. kowalevskii
S. purpuratus
B. floridae
C. intestinalis
H. sapiens
Bilateria
Ecdysozoa
Spiralia
Deuterostomia
Signal peptide
EGF
Transmembrane
Immunoglobulin I-set
Neuregulin

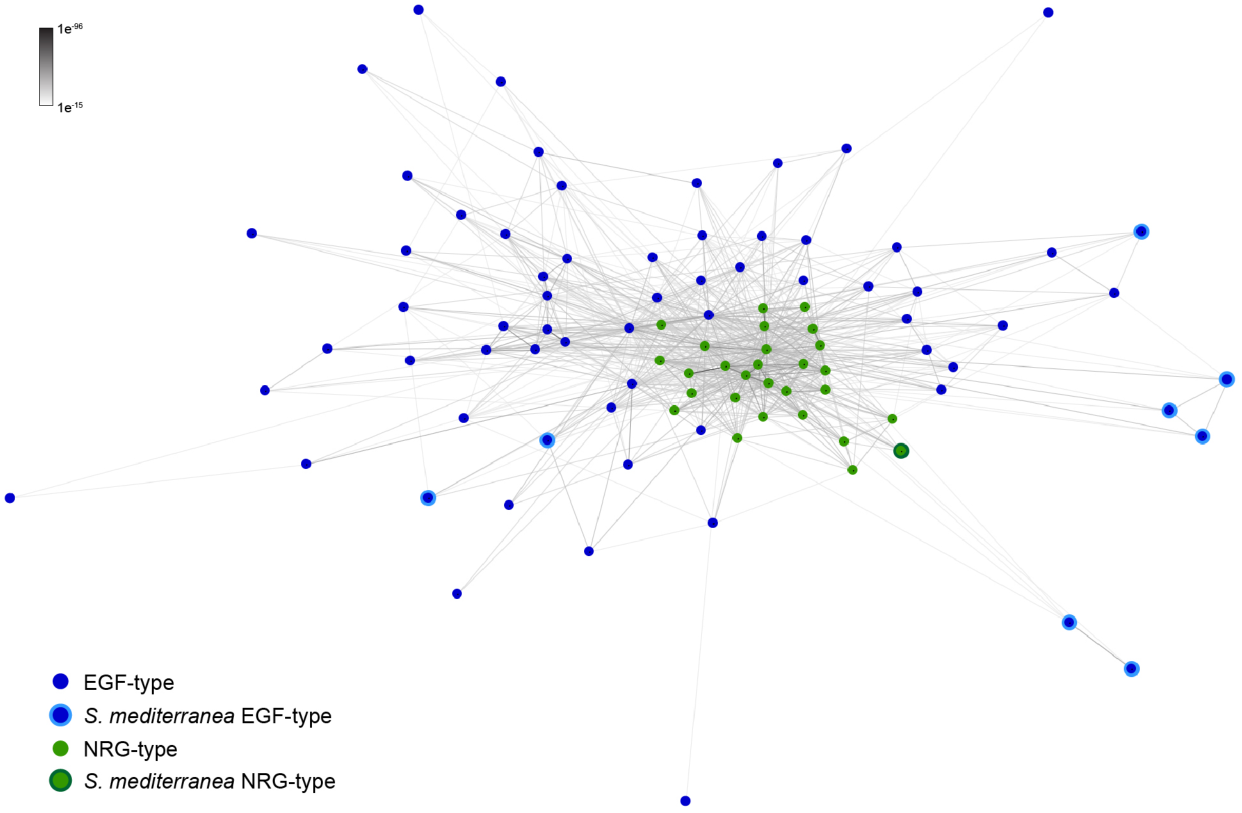
1e-96
1e-15
EGF-type
S. mediterranea EGF-type
NRG-type
S. mediterranea NRG-type

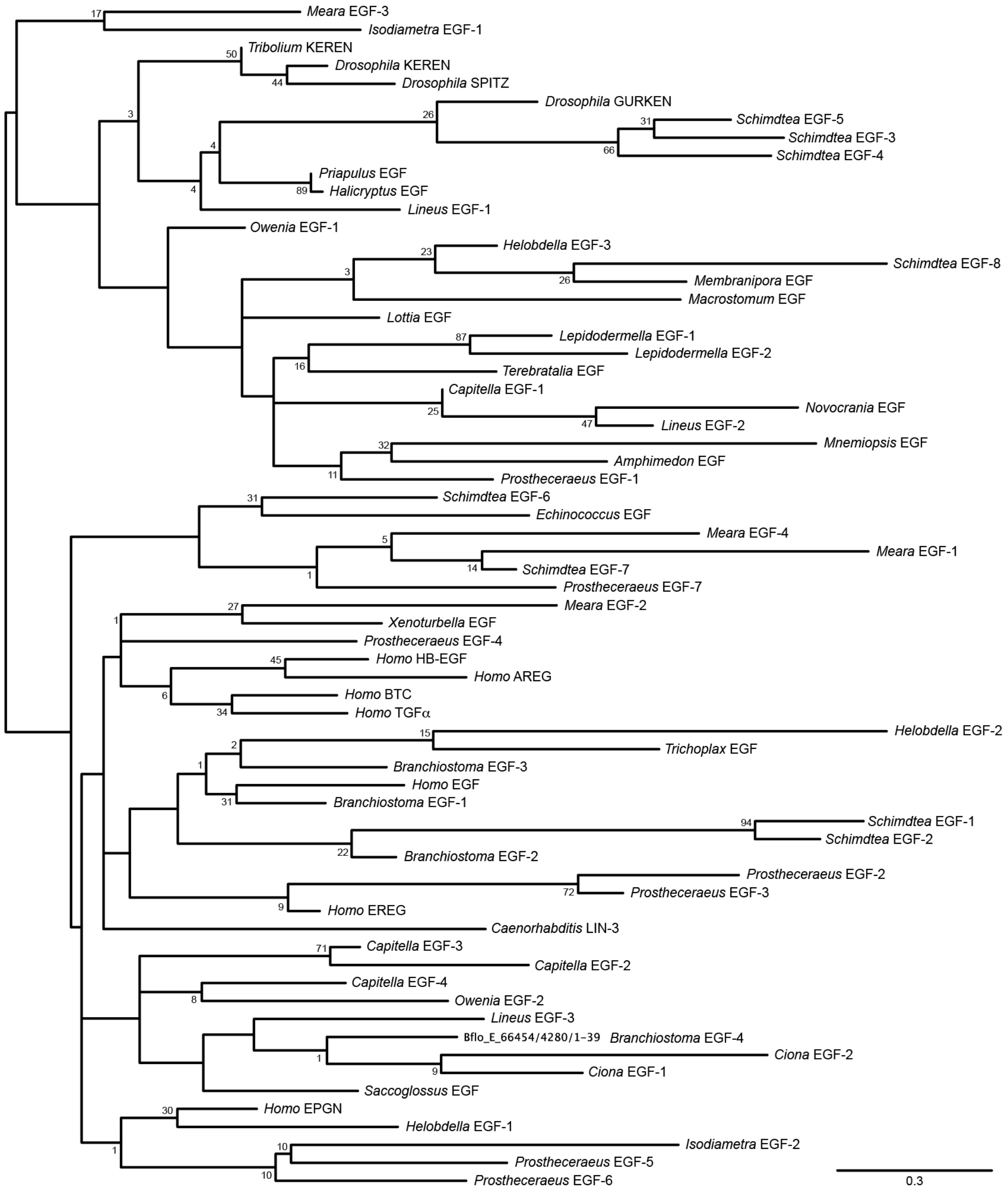
Meara EGF-3
Isodiametra EGF-1
Tribolium KEREN
Drosophila KEREN
Drosophila SPITZ
Drosophila GURKEN
Schimdtea EGF-5
Schimdtea EGF-3
Schimdtea EGF-4
Priapulus EGF
Halicryptus EGF
Lineus EGF-1
Owenia EGF-1
Helobdella EGF-3
Schimdtea EGF-8
Membranipora EGF
Macrostomum EGF
Lottia EGF
Lepidodermella EGF-1
Lepidodermella EGF-2
Terebratalia EGF
Capitella EGF-1
Novocrania EGF
Lineus EGF-2
Mnemiopsis EGF
Amphimedon EGF
Prostheceraeus EGF-1
Schimdtea EGF-6
Echinococcus EGF
Meara EGF-4
Meara EGF-1
Schimdtea EGF-7
Prostheceraeus EGF-7
Meara EGF-2
Xenoturbella EGF
Prostheceraeus EGF-4
Homo HB-EGF
Homo AREG
Homo BTC
Homo TGFα
Helobdella EGF-2
Trichoplax EGF
Branchiostoma EGF-3
Homo EGF
Branchiostoma EGF-1
Schimdtea EGF-1
Schimdtea EGF-2
Branchiostoma EGF-2
Prostheceraeus EGF-2
Prostheceraeus EGF-3
Homo EREG
Caenorhabditis LIN-3
Capitella EGF-3
Capitella EGF-2
Capitella EGF-4
Owenia EGF-2
Lineus EGF-3
Bflo_E_66454/4280/1-39 Branchiostoma EGF-4
Ciona EGF-2
Ciona EGF-1
Saccoglossus EGF
Homo EPGN
Helobdella EGF-1
Isodiametra EGF-2
Prostheceraeus EGF-5
Prostheceraeus EGF-6
0.3

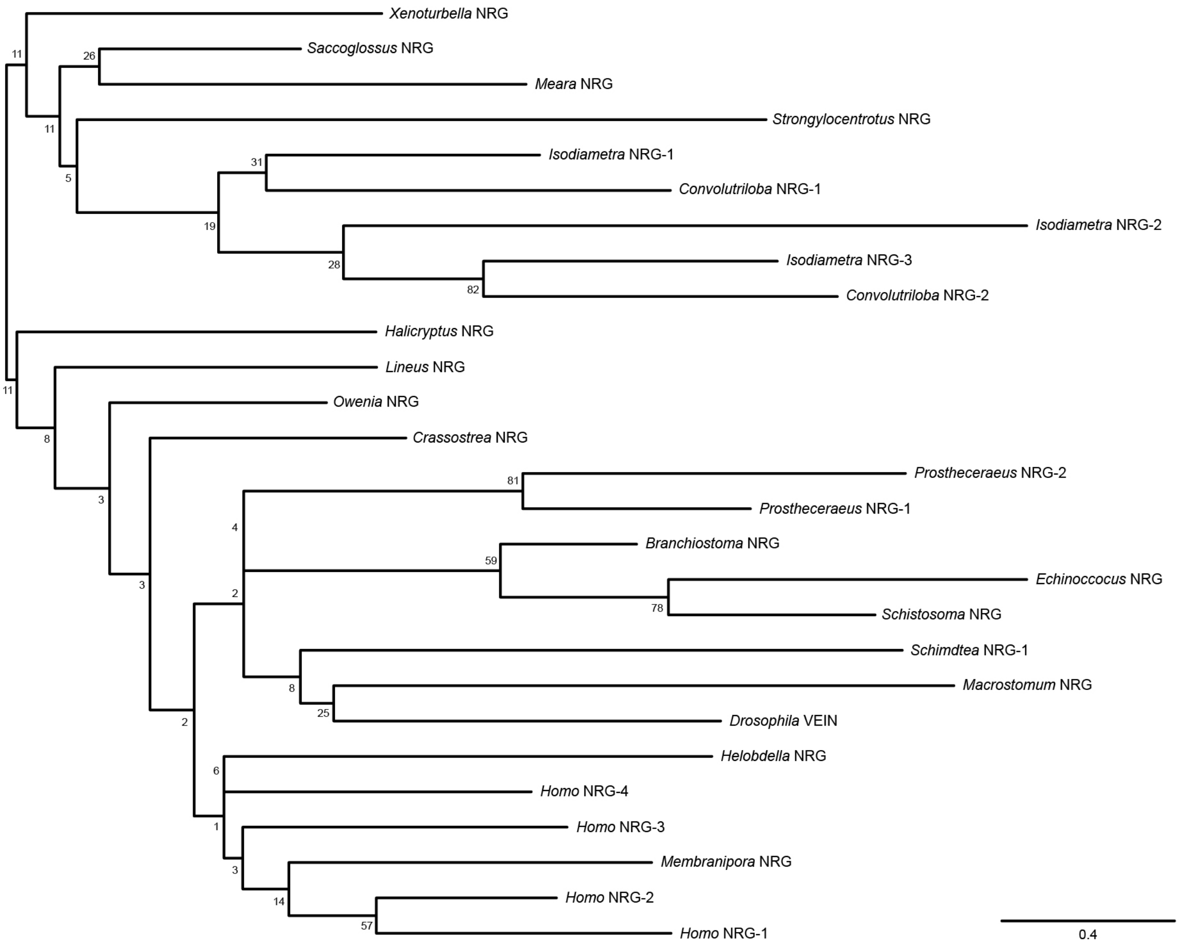
Xenoturbella NRG
Saccoglossus NRG
Meara NRG
Strongylocentrotus NRG
Isodiametra NRG-1
Convolutriloba NRG-1
Isodiametra NRG-2
Isodiametra NRG-3
Convolutriloba NRG-2
Halicryptus NRG
Lineus NRG
Owenia NRG
Crassostrea NRG
Prostheceraeus NRG-2
Prostheceraeus NRG-1
Branchiostoma NRG
Echinoccocus NRG
Schistosoma NRG
Schimdtea NRG-1
Macrostomum NRG
Drosophila VEIN
Helobdella NRG
Homo NRG-4
Homo NRG-3
Membranipora NRG
Homo NRG-2
Homo NRG-1
0.4
11
26
11
5
31
19
28
82
11
8
3
81
4
59
2
78
3
8
25
2
6
1
3
14
57

| Group | Species | Genome/Transcriptome | Source | Gene ID/SRA ID | Tree ID |
|---|---|---|---|---|---|
| Ctenophora | *Mnemiopsis leiydi* | Genome | NHGRI | ML056938a | |
| Porifera | *Amphimedon queenslandica* | Genome | EnsemblMetazoa | Aqu1.218998 | |
| Placozoa | *Trichoplax adhaerens* | Genome | EnsemblMetazoa | | – |
| Cnidaria | *Nematostella vectensis* | Genome | EnsemblMetazoa | | – |
| | *Hydra magna* | Genome | Metazome | | – |
| Xenacoelomorpha | *Xenoturbella bocki* | Transcriptome | SRA | SRX1343818 | EGFR-2 |
| | | | | | EGFR-1 |
| | *Meara stichopi* | Transcriptome | SRA | SRX1343814 | EGFR-2 |
| | | | | | EGFR-3 |
| | | | | | EGFR-1 |
| | *Isodiametra pulchra* | Transcriptome | SRA | SRX1343817 | EGFR-5 |
| | | | | | EGFR-4 |
| | | | | | EGFR-1 |
| | | | | | EGFR-3 |
| | | | | | EGFR-2 |
| | *Convolutriloba macropyga* | Transcriptome | SRA | SRX1343815 | EGFR-3 |
| | | | | | EGFR-2 |
| | | | | | EGFR-1 |
| Priapulida | *Priapulus caudatus* | Transcriptome | SRA | SRX507009 | |
| | *Halicryptus spinulosus* | Transcriptome | SRA | SRX1343820 | |
| Nematoda | *Caenorhabditis elegans* | Genome | Uniprot | P24348 | LET-23 |
| Arthropoda | *Drosophila melanogaster* | Genome | Uniprot | P04412 | DER |
| | *Tribolium castaneum* | Genome | GenBank | XM_008193272.1 | |
| Phoronida | *Phoronopsis harmeri* | Transcriptome | BioProject | PRJNA289349 | |
| Brachiopoda | *Terebratalia transversa* | Transcriptome | SRA | SRX1307070 | |
| | *Novocrania anomala* | Transcriptome | SRA | SRX1343816 | |
| Gastrotricha | *Lepidodermella squamata* | Transcriptome | SRA | SRX1000997 | – |
| Bryozoa | *Membranipora membranacea* | Transcriptome | SRA | SRX1121923 | EGFR-1 |
| | | | | | EGFR-2 |
| | | | | | EGFR-3 |
| Nemertea | *Lineus ruber* | Transcriptome | in-house | | |
| Annelida | *Owenia fusiformis* | Transcriptome | in-house | | |
| | *Capitella teleta* | Genome | JGI | e_gw1.241.3.1 | |
| | *Helobdella robusta* | Genome | JGI | 119222 | EGFR-5 |
| | | | | 109845 | EGFR-2 |
| | | | | 189246 | EGFR-3 |
| | | | | 190996 | EGFR-4 |
| | | | | 81484 | EGFR-6 |
| | | | | 90657 | EGFR-1 |
| Mollusca | *Lottia gigantea* | Genome | JGI | 176100 | EGFR-1 |
| | | | | 139618 | EGFR-2 |
| | *Crassostrea gigas* | Genome | EnsemblMetazoa | CGI_10022473 | EGFR-2 |
| | | | | CGI_10000953 | EGFR-1 |
| Platyhelminthes | *Macrostomum lignano* | Transcriptome | SRA | SRX1343824 | EGFR-4 |
| | | | | | EGFR-1 |
| | | | | | EGFR-2 |
| | | | | | EGFR-3 |
| | | | | | EGFR-5 |
| | | | | | EGFR-6 |
| | | | | | EGFR-7 |
| | | | | | EGFR-8 |
| | *Schistosoma mansoni* | Genome | EnsemblMetazoa | Smp_165470 | EGFR-1 |
| | | | | Smp_093930 | EGFR-2 |
| | | | | Smp_152680 | EGFR-3 |
| | *Echinococcus multilocularis* | Genome | Sanger Institute | EmuJ_000617300.1 | EGFR-1 |
| | | | | EmuJ_000075800.1 | EGFR-2 |
| | | | | EmuJ_000969600.1 | EGFR-3 |
| | *Prostheceraeus vitattus* | Transcriptome | SRA | SRX999630 | EGFR-1 |
| | | | | | EGFR-5 |
| | | | | | EGFR-3 |
| | | | | | EGFR-2 |
| | | | | | EGFR-4 |
| | | | | | EGFR-6 |
| | *Schmidtea mediterranea* | Transcriptome/Genome | SmedGD 2.0 | SMU15040483 | EGFR-1 |
| | | | | SMU15002279 | EGFR-2 |
| | | | | SMU15001020 | EGFR-3 |
| | | | | SMU15031449 | EGFR-4 |
| | | | | SMU15016442 | EGFR-5 |
| | | | | SMU15034627 | EGFR-6 |
| Chordata | *Branchiostoma floridae* | Genome | D'aniello S. et al. 2008. Mol Biol Evol 25:1841-1854 | | |
| | *Ciona intestinalis* | Genome | GenBank | XM_002122823 | EGFR-1 |
| | | | | NM_001078516 | EGFR-2 |
| | *Homo sapiens* | Genome | Uniprot | P00533 | ERBB1 |
| | | | | P04626 | ERBB2 |
| | | | | P21860 | ERBB3 |
| | | | | Q15303 | ERBB4 |
| Hemichordata | *Saccoglossus kowalevskii* | Genome | Metazome | Sakowv30039288m | |
| Echinodermata | *Strongylocentrotus purpuratus* | Genome | Uniprot | W4XYS6 | |

| Group | Species | Genome/Transcriptome | Source | Gene ID/SRA ID | Tree ID |
|---|---|---|---|---|---|
| Ctenophora | *Mnemiopsis leydi* | Genome | NHGRI | ML238312a | |
| Porifera | *Amphimedon queenslandica* | Genome | EnsemblMetazoa | 205916 | |
| Placozoa | *Trichoplax adhaerens* | Genome | EnsemblMetazoa | T53737 | |
| Cnidaria | *Nematostella vectensis* | Genome | EnsemblMetazoa | | – |
| | *Hydra magna* | Genome | Metazome | | – |
| Xenacoelomorpha | *Xenoturbella bocki* | Transcriptome | SRA | SRX1343818 | |
| | *Meara stichopi* | Transcriptome | SRA | SRX1343814 | EGF-2 |
| | | | | | EGF-3 |
| | | | | | EGF-1 |
| | | | | | EGF-4 |
| | *Isodiametra pulchra* | Transcriptome | SRA | SRX1343817 | EGF-1 |
| | | | | | EGF-2 |
| | *Convolutriloba macropiga* | Transcriptome | SRA | SRX1343815 | – |
| Priapulida | *Priapulus caudatus* | Transcriptome | SRA | SRX507009 | |
| | *Halicryptus spinulosus* | Transcriptome | SRA | SRX1343820 | |
| Nematoda | *Caenorhabditis elegans* | Genome | Uniprot | Q03345 | LIN-3 |
| Arthropoda | *Drosophila melanogaster* | Genome | Uniprot | P42287 | GURKEN |
| | | | | Q01083 | SPITZ |
| | | | | Q9VVJ6 | KEREN |
| | *Tribolium castaneum* | Genome | GenBank | D6WGH5 | KEREN |
| Phoronida | *Phoronopsis harmeri* | Transcriptome | BioProject | PRJNA289349 | – |
| Brachiopoda | *Terebratalia transversa* | Transcriptome | SRA | SRX1307070 | |
| | *Novocrania anomala* | Transcriptome | SRA | SRX1343816 | |
| Gastrotricha | *Lepidodermella squamata* | Transcriptome | SRA | SRX1000997 | EGF-1 |
| | | | | | EGF-2 |
| Bryozoa | *Membranipora membranacea* | Transcriptome | SRA | SRX1121923 | |
| Nemertea | *Lineus ruber* | Transcriptome | in-house | | EGF-2 |
| | | | | | EGF-1 |
| | | | | | EGF-3 |
| Annelida | *Owenia fusiformis* | Transcriptome | in-house | | EGF-1 |
| | | | | | EGF-2 |
| | *Capitella teleta* | Genome | JGI | 212613 | EGF-1 |
| | | | | 200359 | EGF-2 |
| | | | | 215694 | EGF-3 |
| | | | | 190880 | EGF-4 |
| | *Helobdella robusta* | Genome | JGI | 183201 | EGF-2 |
| | | | | 190013 | EGF-3 |
| | | | | 163186 | EGF-1 |
| Mollusca | *Lottia gigantea* | Genome | JGI | 154532 | |
| | *Crassostrea gigas* | Genome | EnsemblMetazoa | | – |
| Platyhelminthes | *Macrostomum lignano* | Transcriptome | SRA | SRX1343824 | |
| | *Schistosoma mansoni* | Genome | EnsemblMetazoa | | – |
| | *Echinococcus multilocularis* | Genome | Sanger Institute | EmuJ_000753300.1 | |
| | *Prostheceraeus vitattus* | Transcriptome | SRA | SRX999630 | EGF-3 |
| | | | | | EGF-6 |
| | | | | | EGF-4 |
| | | | | | EGF-7 |
| | | | | | EGF-1 |
| | | | | | EGF-2 |
| | | | | | EGF-5 |
| | *Schmidtea mediterranea* | Transcriptome/Genome | SmedGD 2.0 | SMU15012052 | EGF-1 |
| | | | | SMU15031576 | EGF-2 |
| | | | | SMU15029762 | EGF-3 |
| | | | | SMU15026774 | EGF-6 |
| | | | | SMU15008518 | EGF-7 |
| | | | | SMU15007841 | EGF-4 |
| | | | | SMU15013751 | EGF-8 |
| | | | | SMU15014334 | EGF-5 |
| Chordata | *Branchiostoma floridae* | Genome | JGI | 107093 | EGF-2 |
| | | | | 107092 | EGF-1 |
| | | | | 66454 | EGF-4 |
| | | | | 118182 | EGF-3 |
| | *Ciona intestinalis* | Genome | GenBank | 215217 | EGF-1 |
| | | | | 220015 | EGF-2 |
| | *Homo sapiens* | Genome | Uniprot | P01133 | EGF |
| | | | | P01135 | TGF-alpha |
| | | | | Q99075 | HB-EGF |
| | | | | P15514 | Amphiregulin |
| | | | | P35070 | Betacellulin |
| | | | | O14944 | Epiregulin |
| | | | | Q6UW88 | Epigen |
| Hemichordata | *Saccoglossus kowalevskii* | Genome | Metazome | Sakowv30036701m | |
| Echinodermata | *Strongylocentrotus purpuratus* | Genome | EnsemblMetazoa | | – |

| Group | Species | Genome/Transcriptome | Source | Gene ID/SRA ID | Tree ID |
|---|---|---|---|---|---|
| Ctenophora | *Mnemiopsis leydi* | Genome | NHGRI | | |
| Porifera | *Amphimedon queenslandica* | Genome | EnsemblMetazoa | | |
| Placozoa | *Trichoplax adhaerens* | Genome | EnsemblMetazoa | | |
| Cnidaria | *Nematostella vectensis* | Genome | EnsemblMetazoa | | |
| | *Hydra magna* | Genome | Metazome | | |
| Xenacoelomorpha | *Xenoturbella bocki* | Transcriptome | SRA | SRX1343818 | |
| | *Meara stichopi* | Transcriptome | SRA | SRX1343814 | |
| | *Isodiametra pulchra* | Transcriptome | SRA | SRX1343817 | NRG-1 |
| | | | | | NRG-3 |
| | | | | | NRG-2 |
| | *Convolutriloba macropyga* | Transcriptome | SRA | SRX1343815 | NRG-1 |
| | | | | | NRG-2 |
| Priapulida | *Priapulus caudatus* | Transcriptome | SRA | | |
| | *Halicryptus spinulosus* | Transcriptome | SRA | SRX1343820 | |
| Nematoda | *Caenorhabditis elegans* | Genome | Uniprot | | |
| Arthropoda | *Drosophila melanogaster* | Genome | Uniprot | Q94918 | VEIN |
| | *Tribolium castaneum* | Genome | GenBank | | |
| Phoronida | *Phoronopsis harmeri* | Transcriptome | BioProject | PRJNA289349 | |
| Brachiopoda | *Terebratalia transversa* | Transcriptome | SRA | SRX1307070 | |
| | *Novocrania anomala* | Transcriptome | SRA | SRX1343816 | |
| Gastrotricha | *Lepidodermella squamata* | Transcriptome | SRA | SRX1000997 | |
| Bryozoa | *Membranipora membranacea* | Transcriptome | SRA | SRX1121923 | |
| Nemertea | *Lineus ruber* | Transcriptome | in-house | | |
| Annelida | *Owenia fusiformis* | Transcriptome | in-house | | |
| | *Capitella teleta* | Genome | JGI | | |
| | *Helobdella robusta* | Genome | JGI | 167059 | |
| Mollusca | *Lottia gigantea* | Genome | JGI | | |
| | *Crassostrea gigas* | Genome | EnsemblMetazoa | EKC24032 | |
| Platyhelminthes | *Macrostomum lignano* | Transcriptome | SRA | SRX1343824 | |
| | *Schistosoma mansoni* | Genome | GeneDB | Smp_136660.1..pep | |
| | *Echinococcus multilocularis* | Genome | Sanger Institute | EmuJ_000090400.1 | |
| | *Prostheceraeus vitattus* | Transcriptome | SRA | SRX999630 | NRG-1 |
| | | | | | NRG-2 |
| | *Schmidtea mediterranea* | Transcriptome/Genome | SmedGD 2.0 | SMU15028233 | NRG-1 |
| Chordata | *Branchiostoma floridae* | Genome | JGI | 123722 | |
| | *Ciona intestinalis* | Genome | JGI | | |
| | *Homo sapiens* | Genome | Uniprot | Q02297 | NRG-1 |
| | | | | O14511 | NRG-2 |
| | | | | P56975 | NRG-3 |
| | | | | Q8WWG1 | NRG-4 |
| Hemichordata | *Saccoglossus kowalevskii* | Genome | Metazome | 30025066m | |
| Echinodermata | *Strongylocentrotus purpuratus* | Genome | Uniprot | XM_001194757.1 | |

EGF RECEPTORS
>Mnemiopsis leiydi EGFR
MHFCSACNTFSSLLLLLLLTFTEAVFYSDRTGTTCFSAYDENKFYAITLEHRKTIKELEKFAGCEIIIGNLILRNITDPDGTK
LQFFSSVKKITGYIAVFDCEMPYLPFPNLQIISGQNLIPEKKKPKIALFVSNVRNTLHTGLNSLREVSNGHVLMENNSHYLTG
YSNKIMWDDILLDNATATVKTNSTDFDLNDLSMQVCQDQCNGGRCYFAEDSSYSPICCHKECSGGCTGMTNADCIGCKNFNAT
GQCVTNCPPPKRYDKALFKMVDNPDVLYGLGNECLEECPIPFLVEEGNCVKKCSDHLMTEPDTNKCVPCPKEGCPKRCDGFDS
VSNLALGRCNEFKAKSKKCSKLFQEKYTNCTIIEGNIDIDSKFTDKFGTNTADALEALSTVEEITGYLSIHTNNLFTNFRFLR
NLKIIRGRNLKASYSNSCDDANVGCRVTFQAAITVQAKVCMECHDKCTMDRGCTGPNSFIGPGGCNFCEVVILMEETFTENNT
RRLWLSRVLTAVQALTTPCTTSSNQRVEPLARDSRGPIRTLACRPVTLVGQSAGRPGADCISSDTVPLDRKGIVGFANCNLVE
GSIIINYQSWTGPNNITTADLEALDEIVTITGRLIIRDSPDPNFTSLDMFRRLAEVKGESTQRKRRRRRAADDFVIEIDNNTA
LETAELVNLKKIGGPMKLSGSNLCYVDRVDWGSRLGDQQVTVDISESSCENKPCHSECSETDGCLGPDRDMCITCKNKEFEGV
CTPTCQGESYDDGTVLCKPCHENCNTCSGPGNHLCLSCKHVFDGAACVTRCSDPHSYVQNKQCVQCSSACEGGCDGPGSHKGV
GGCTTCKLFDNTVDVEKQICADKCPAKSYKDTIQGEDGEYITVCEKCHEECAECDHWSDSSCSKCVHVRQGDRCQAECDQGFY
QGPDKTCLRCDKECAVCIGPGDRNCTACENFELPSSEIKLTRTCVEECPDEYSKTQKVILNEHQEHKRCVMECEDGLDPHTMR
DEDYCKCIPGRACPGTAPRENSTAVAVAVGVAIVFIIAVVSFICWKRAKQNQKKRQNLLKHAQQMHDNINPIYEPVMQEDTVY
PLNDGQLLIVTEDSLEIGEVLGSGAFGKVYEGRWHPAENLNTTDNKGFRVAIKILKDSGDPESTREFFDEAVVMGKLDHKHLV
RILCLCAGKMMLVTQLMPLGSLLDYIKPYNFKHKEIFSVQTPTFVKITDFGLAKCLSSKKQMFTSEGGKLPIKWLAIESLKYR
QFTSHSDVWSYGVTIWEVLEFGQLPWRTKPPAELIRSLEMGERLPKPKTCTLEVYALMLRCWVLEPTARPNFESLECSMDEFL
KEPNRFVHTPHNNRASTEGSFLSPHASVTSEYRDSGIVDVDRNYENDDRAMAMQRALYDATSSLRPHENSSSNYDPALTADQD
DVFSESPSTARNSVIENRHDYVQDYLVPDQYSVDEGNGTDSSQDQGYVKMNGSDYADLEQQPEYSNADALLDSELTQSLLEHS
KQSNKNNKNKFPYNSVTSSILSTVFYLAQLDNTSWLVYSIISRILQGFAVGIIEVRFLDMMTQCFPDNLALISSAYELPFCFC
MIVGFEMGGWVYEMFGFWAPLNLTTVLVVVTCIAAYFCSYWLPENEEDDVSATISDILYWPILVISLLVMGSISIADSIANSF
YSNYMLNRFGLSETKSGGMLTIASIIYTITTFISGCIGSRQKNLKYLLLGGLILMGTSACLLGEDIPIFNTMGVYFPATMLCL
LQAGSGLAQVAALPLMVMQYEKRTGLPENLASSHMGGIYSSYFFLGSFLGPLAGGYVIDITSYGFICTICGILLLVSFQIMMG
LQLAKKQFLNP
>Amphimedon queenslandica EGFR
MLFELGTQCVEDCPSNYMPVVNNGIMRCLSCDGPCPQECTGGVFDYNDPGKLTGCTVITTELRISSVPPNVNLSILNDLNGIR
EIRGSLDISGFNKNTFPYLSNLKTVGNDSTQVLSQSCNGSSDSTKFSVIIADTDLISIDLSSLETVINGGIRLQNNPSLCYLG
NLSYYLANASSSSCVLDNHRRRIDECVEMSMTCHPQCSSASGWCWGPNDTQCVTCTNFIFNGQCVPDCHDFDTYGIHLFNHNA
NNTCLPCHSFCNGSCVGEGPANCSSCNTYFFVNVSIGVGKLCLESCVGDTYLPDDCTSGECLPCFDGCTLSNGCAGPGKTFNN
SNGCIACDTILLDRNGEQIECQRDVTCPSGYYREILSEDRGIFFTGTVLCHSCHELCATCSGPSVSDCVLCSYIRGTNGSCVY
MCDPIRETVPMNGSSQCILVTGTMGTLDPTGAIVSIEAVIGSIFGGLTLITLTVLVILILVLFLLRKNRKSIYIAELELCNEK
RQSKVPPDATKLILIPETELEFKKELGSGAFGTVYEGYWKPDNDEAEYHVAIKILKNTSADASKELLQEGVVMATXXXXHRNS
ISAQTVINFSLQIAKGMAYLESKSLVHRDLAARNVLVQSHLKVLITDFGLTRFIEPDESHYEAPGSKLPVRWLPPESIQQRIF
THKTDVWSFGVTVWEILTFGESPFKGKSVFELLKLLEKGDRLKQPKTCTLEFYKTLQNCWELEPERRPSFEDLVYQFNTMMMS
PKKYEDSNDKDGYVDCLQENFNDKDGYVDCLQEDFNDKDGYVDCLQEDSNDGYVDCFNEGPNDAIVTKTLATSIA
>Xenoturbella bocki EGFR-2
MIFVIHSLWKETMGKPTSHPTLNLLITVIVSLLYTSQAQVNYFKECTGTFSYTSSSVNSEEQYLKLKNRYEGCTYVEGNLEIV
SVADPNYDLSFLSNITEVTGYVLISFIYVDLMPLDNLRIIHGRTLYEDEFGLFIKYNDMPGNRMIGLKQINLHNLREIVNGVV
QIKNNRFLCYVHTIEFEDIVNAGAVINNQINITSDCGECHPECETMRCWGPELEDCQILTRTICSEQCDHRCSGLSPRDCCHR
ECAGGCTGPTEYDCIGCLRFNNEGVCLSRCPPKYVYNDETYSNEPNSDFRYSLGVRCVAECPDPYLAEDEGCVKMCGSGNMAV
GGECVDCEGPCPKTCIVTGVVNSENIDDLNNCTIIEGNIDISYYGLNGDTFYPNLTPLAVSQLEVLSTVQEISGYLNIGAAHT
VNLTNLNFLRNLRSIWGQQQYNDFFTLTVAPNTYLESLGLVSLEVVKSGSIQVVANPKLCYVNSNTLQTLVSSSDQSVMFIGS
KSSEECDGEGFVCDELCDITGDTGCWGPGPDMCLNCANYTLANSCVERCHPDDGYYESAPATCEYCDPQCSGGCVGPGPSNCT
SCLFYSDGPFCVSQCPATKYGSEDGVCYTCNDVCREGECTGPGAFKGEGGCRNCYVLVDYENGTNVCLMEDDVCPDHFYYTFE
KDEYELFTKTKVCHECDVECLQCTSDGPFRCDGCVHYMSDGECVSECQYDRYPTENNVCEKCDYECSTGCTGGNSSSCDSCKN
YYIILDDVKECVQQCPDEHPYRHYSDMECLDACPQSYRKNSNGICIAVTTNGAVGGIIGGVVVFLLILFIIGAVIYIRRKKKR
KPDTLDGLPLDYIGGEAQPLTPTGAAPNEATLRIIMVTQLEKGGILGSGAFGTVYKGIWLPEETEDEVRIPVAIKVLHEGSAQ
AKKELLEEAYIMATVDHPCLVRLLAVCMCDEMMLVTQLMPHGSLLDYIRGNKVRIGSQALLNWCRQIAMGMSYLEEKRLVHRD
LAARNVLVQTPTQVKITDFGLARILDIHEDEYTAAGGKMPIKWLALESITYRKFTHQSDVWSFGVTVWELMTFGAKPYEGVTA
RDVPDLLQKGERLPQPPICTIDIYLLMLKCWMSDPDCRPCFTELIEEFGKMARDPTRYLVIEKRSIMVRKSRSGEAHVSPPPP
YPAVTDAYAKLTHDNKLKIPEAAYYSESLTGKQKPPLPPTNADGLVDAEDYLQPELRPKEQYASMDGSYSQEPNPGTRYCDDP
TTPNVFDEMDAPNEVFDYSNGDYNNQYQSLDNSPSAEYINSPQEDKVEYLPPSPPQPAVTNPEYLTYGDDAESEPDPDHEYIN
NPSGGGRGVQLGGVDADDRESMV
>Xenoturbella bocki EGFR-1
MSSVAHHTGLVVLLALFWAAEHGTSHCMKVCEGTGHSIQKFAPQDQDQHYIDLKNRYTNCTYVDGNLEVTGIRDPNLDLSFLL
DITEVSGYVSILDVYTDTVPLDNLKIIHGRTLFDGYALYVWLNHQQVNSGRKIGLKDLRLQSLKEIVKGNVEFINNPYLCHIH
TVIWADILSSGYNASVDGAVCGECSAVCPVASCWGAGDDQCQTFTKINCSLQCDHRCMGPSPSDCCHPECAGGCTGPRPTDCV
GCRNFDDDGSCKADCPRSQIYISSEYKYVYNPDFKYTFGTRCVRECPVEFVAEDDGCVKWCSHGKEAKDGLCLPCEGPCQKTC
PGIIHANERVEIEAVNANNINQFMNCTSIVGNLHILEQTFTGDDFYGIPPLDISALSILNDVREITGYIYIHSGLDVPLESIS
FLQNLEVIRGRELLRNRALVIVRTTVSYIGLTSLTTIEHGDVVIENNNNLCYTTSELFHNTVDNSQMIRISDNKDISQCEREG
KECSRLCIADNDPIGCWGPAPEQCLECAYFDLQGECVRRCDTRHGLLYISAPGICEHCHPECADSCHGPGSDNCTMCADYKDG
PYCVPECPLMKYPDAYNECQDCARLCREDGCTGSGNFVGLGGCNSCDKVVLYPNQTQSCVEPEIICTDDFFLDRYPLNSKSEL
AGHLVCEACDRECIGCTNRGPFNCVKCAHYLQSGMCVSQCSAYKYISEESECLECHYECRQGCTGGTEYHCDSCRNYAVLNST

NNATQLVKCVSSCPADLPYIIDDVDMSLCVSECEGLTYPNNDTMKCTSCHTECLDGCTNDLRSGCNMCVNVRHNGDCVDRCPD
DEYTVDEFGVCLAIQKQGEQSADNIFKDNIALVVSIMGVCLVVGVVLCVCSVWRMKSTTIKKNSIRLDTLDCVTEPLTPSGAS
PNQAQLRIVKETELRKAGILGSGAFGTVYKGVWKPADTSDSVVVAIKVLHEGSTQAKKELLEEAYVMATVEDPCLVRLLAVCM
ADEMMLVTQLMPLGSLLEYIRKHRTKVGAQPILNWCRQIAKGMCYLEEKRLVHRDLAARNVLVYSPENVKITDFGLARILDID
KDEYTAEGGKMPIKWLALECIIYRRFTHQSDVWSFGVTVWELMTFGSKPYEGLGAREVPDALQKGERLPQPPICTIDVYLLML
KCWMNDPTCRPTFIELAEEFGKMARDPPRYLVIESNMNMSDLPNLSPTSREQFYKTLLSAEGGADCLTDAEEYLKPQSQATLN
YATMQHAPGFVWPSTSPTNSYNRGLASNYHPLSLMSSRRGDQSQNSYTSDSSSSGYVRHDKPPKKKSFRYCEDPTNSPTSERR
RGIIGSQRSLTSYMGLSPATSADRMSPPKSPNIPSPNAYPMLETVHRQRPFLDDAPQARGGACSSGRKPHPLAIPPHPLTIHP
AHNANIPVTNPDYVDSFKPSYKDTSVEEDSEEYLSDHDYYNDTSITVPTILPETCALMNGRCKQTDIAT
>Meara stichopi EGFR-2
MGGTPSNIIYLVYFLIWLTISSIFAVDGTYISLNGLKECPGTNNGISMGSAFVSVNITKLKNRYNECQRVQGNLEITHINPKN
PAAEDLQFLSSIIEVTGYVLFYNTSVDISALKNLRFIRGLSQFKKGNVFYVAKTNIRYLGFRNLTQINGGVYIVENVNLCYVD
TIDWNDIFGGKNKFDLDVDKAATCTACSPTCDGGACWGPGPDNCQIKTFQHERCEADCSRRSRCMPGGLCCDANCASGCYEKK
DQCLACNDFEQDGECDNKCSSQKYDPSTGDMKNVPHYKYFVTCVKHCPAQYSIFVEDSACVRHCPSGFTEATDNNVKVCKECE
GPCPKTCNVAKFIGKDGTLSADNLPSFVNCTTIQGNLAIGGTASHGLTLIGLSVLSNVKIITGKLSVTIDKALDIANLDFLSN
LEEIRGIAGQDSMQVVVNADSVNFKYLGFKSLHFLGVKKTVNMLFINGICYLSEPALKSAFHSVDFSIRHVVAVSDLSTCNSS
VIGCDSECGPAASSSVDAGCWGPKASHCFRCKNFLKKNACVDSCEPNSGYYINGSNCSKCHAECESSCNGRTAKNCIGGSDDP
EGHGCRHVSHEGKCLAKCPSLMYPDSQQICQKCSNKCDGNYECFGPSNLIGANNDGCGYCKYGKIVKLKGREEVACRLDDEQC
TASQYKDPRSMANAVSDNHIEVCTDCHDQCEGCKGRDDDDCKNCKHFKVVKSLGNFQSGFTCSSKCPSGTYLLRKNNPDVGAA
GICVPCHEMCGVDVDGQSCVGSAETDCTVCKVFKEYRSNVTYHNVTSDTPFACVASCPEMKITSTSTCVNVCPPLFFNVSREC
ESCDQECDGGCVGPRKDQCNRCRNLEYLGACVAECPRGSEISPDDDTECIRKMLSGGAIAGIVSGCLVLLVAGFIFFCFYYKR
GVKKSEEKVKRQFSDFDVCLQPLNPKLPPNKAYLRMFTENELKLHGELGSGAFGTVYKGTWIPEGTESVKKALVAIKVLRETS
REAANDILEEAKMMASVVHPNLVRLLGICVTRQIMLITQLMENGNLLDYVQKHKLKVNAKHLLDWCLQIGKGMEYLEAKRVVH
RDLAARNVLVHTSKTVKISDFGLSKVLESGEEEYIEAGGKLPIKWLAIEVFEKKAFTHQSDVWAFGVTMWEIITLGARPYEGV
RSSDMYELLEKGERLPQPPICTLDVYMMMLRCWMLESHVRPAFHDLVTELAVFVRDPLRYLVIQSNYGDDCLDSPYNDLSDLF
SPDDDYVDPEMYLPFYCSVEPDGLVNIDDLDDASEWQRKKKIQMMYPDENPDDLVDADDYLMPQSGNMVGGSQPGHKSQTMGQ
LAYGANNDCYDITNGSKTAPSGVLSNPINLMSPTDDYFANSLPRDYQVRGMDDIPEGLYSGTRDNSTYQNSESIA
>Meara stichopi EGFR-3
MQRDKTSDRAGHSRRRTTTHVTIVLLLSLSCQVKGDLNGGQIKARKECNWSNNAKSSGGVRKFYQAMEKEMTNCTILVGNLRI
EVFEGESDLYSFSFLEHIEEITGYLVVISSEMTIFNMKRLRVIRGDSLYEKTYSMVISHNRRLLELSMPNLKEIITGNVRIWQ
NEKLCYINKIHWNDMFNPEEELRNRTQKAILPDPRNHCKDVKCHDSCRNCWGPRKSDCQNQTLLICSRQCDYRCKGPLVEDCC
HKECAGGCYGYTNRECFACKGFENNGECLAMCPPLQTYDTSTGEMIDNKNGKVSYKRKCLHECPLNYPRNPNGFCVNHCDEGM
MAVDGICVKCTTICPKICDGIFTGKDQLDAPVGYINGDNIDSFKGCTTVRHNIVINHLSFEENDFPDITKTNSVSIKSLAALN
DVRVVQGYVVIQTGNTDLYNLKFLQNLEEILGQNLFQNVYSLSIEGTKLESLDFTSLKLVRNGNIVIRENSELCYITPRLLES
VLKDKSRQKLIYTQRVTASKCERNDRICHKECARFDGDGNAVPLECWGSGDTNCFRCENMKMRGTCVNTCSPSISFTEPDEKT
CTMCHPECNGGCRGPSNMDCIACKNAIDGKSKRCVAQCAEMKYRDKHNQCQMCESVCNQCTGPNTNLGPDGCAFCDNAYKYRN
GSLQCTGSITSCADGFFLDHHLHNDTTVRVCSECHDACAKCQTQLNFKCEVCKSKLRDKGTCVDFCFNNTFQGEDLAGDHFEC
LPCDSECDSGCYGEGPTNCKRCRHFSVPEESAIPLNRIAHINDTTTVGLTSPVLFTPRSKLKRFCVKHCPSNKPFALKTINGD
LCIAKCPGDFYATKLNWCVHCHLECKGGCRDDTRSNCTECSNVNYMGECRASCPDYTILNVSTMSCDNANLVTLSGQEDKRAF
PVYAILLIILSILVTVIFCSVFGYTRYKKRKDQDRDSLMLSVPESLFPFGAPPNKAQLKIIKETDLRKLELLGGGAFGTVHKA
IWTLPTDPGYPKKQHYVAVKVLHSQTAEKSQEFLDEAYVMASVHHPCIVSLLGICMSQQVQIVTQMMPKGSLLTYVRKQKKKI
GSWQLLTWCEQIANGMAYLEEKRFVHRDLAARNVLVESTKQVRITDFGLAKVLEANIEEYTADGGSMPMKWLALESIQHQSYS
HQSDVWSFGVTVWEMMTFGARPFEDISTRQLFAHIESGERLSQPSICTIEVYMLMIKCWMKDPVARPKFTYLAAEYAKMAEDP
ARYIVIENDGNLVPLSPVEDNRFFSLLSPEESAMYDKEQYLKPQMVGHYSCLEFSPSTLSVNTSIPPDVENGEICETEETSFI
KSGVSKHDPSLRSKGIRMTPQAASMSPERENPLNAAQYKRIDSCERNRNARRQFSNTNLEASQSSTASNAVQNMEYIECGGLD
DSDSCGPTYNSISLNSPLINRCHGTASFNADSDEDYSPDYFNTYEDCFSDPNPGYELMQSPNAVKSHFIPRFKIHPDSQNHTY
INSQVSPIERTSLQSSLDLRPRFPTPARQPRSISESIGRTQFVNNINCPKDTSTPKSKLFPSREDVFDDGSSLSGHINTFGGP
HKLQTFNITDKRTPPDGSAMDSGGVKEDNPLSDDIVALI
>Meara stichopi EGFR-1
MLLYTPIIILAFVVRMVCPVATGHEEVSRTCTGTSSGQSISVNQTQHLRQLRHHFENCTYVIGNVELSSIVDSEEDLSFLGNI
KEITGYLLVIGCRVSVLALRKLEVLRGEQTHKTHAFYLESSTFTDIKMPRLRAIVGSIYIRNIKGFSCFIRTVDWEDILSGEK
KYISRSNFQSVPDSSNCTCSKYCNESCWNVNECQEKTHTICHDNCKFRCLGPNSKDCCDPQCLGGCTAALNNEKCTNCKNYVN
DETGECIELCPPPKVHDPVSNLIVDNVDMMYALGNHCVKKCPPSFSKHIISETTAACVDSCGEGYYKFYENVTDVSSSVCRKC
FNESRCPKYCNFKSTELESDTHPIMAYAGCTTIIGNLNIIWESLQELNTSRDLEALTTITEVTGFVRFQGRMPSTVITTDTVA
DNVTNMSFLRNLRVIGGNDLIGTDYALAVLFTNFEYLGLTSLKTVSKGSIFLLKNRFLCYINETGMKETVNVDGYIKNIENSD
QCEENGFICNNNCKPIGCWGPGPSSCFDCEKWTVMKKPHVCVQECASKGMYVKSEKDMSCGSCHEQCNSRCSGGSAANCTDGC
KYCRDDDNGRICVSDCSGKQRFCGIDGICRSPAIEKFYQNQYFIAGMTIVAIVIVISIILSVFCWRRSISRTQLRLKMEFAQL
DETLELLPTGNIKPNTSKLNMIPRSCLQFRGKLGSGAFGDVSKAIWIIEDKLDEEGKPLKYVVAAKTLNQHSGPMHQKELMEE
AYTMASLQHRNLVRLLGVCMSERIILVSELLMLGSLDVYLKKQKKTIGANHMLLWSKQIAQGMSFLEENHFVHRDLATRNVLL
STPEFVKVADFGLSKMLDDGKPYLNTEAERMPLKWLAIECFAKKVYTHKTDVWAFGVTVWEILTFGQVPYGGVESRSLYPKLK
DGLRLKQPAIATLELYRTLVNCWNFDPEARHNFSSLYQEFNRMYKDPKRYIVIDMKPCEEFNRDNLLMRLLPDDVGISTLPEL
STPVSPISDQDCFFKSGIEREQQPQQPSTFDKSQIWSRNTPRSRSQNKYEDPKPDGDIKENIEDASLNGRHQRSIKNRGYNLP
LEKFSNANSQMYENTDVPSLTYANIPPEN
>Isodiametra pulchra EGFR-5

MKCLIPLVLLAFIFASSDGLPERFEECDGSSMKYGLTDVDPYGSLKQRYGNGKNCTFIDGNLEIRYIIEDTPGQYSFDFLQHI
TEISGYLFIFNVKAQRIALPSLTIIRGKALIGDASLMIKTGAQTLNNRISSPGVTELLMPNLKEITKGNVELANNEAACYLSR
VNFEDMLTDSSSQSIHIGKNGVGSDCGDTECYSGGDGGGNGSCPYPDHCWGPDRDHCQHLTRTVCSDSCGGSRCYGTGGGECC
HEVCAAGCSNHLSTQCAACRTLQLGDACVSHCPTGYIYDPLRHTNIETEKMYSHGFRCVSSCPSGTYIDGQSCVAVCPHDKRV
EGNQCIPCDEKCDKECSVPHIEKEEDADGHTRARYSTSWDVIKGCTTIKGNVFLDWQFWNGDPHTGLPAKGLEVISYFKDVKK
ILGMLHVLQSPLRNLSVFENLQEVRGKLESGKSQVTTLMIAANNNLQYLGLKSLMTVDPRDGTKAATTIVHNPNLCYINNEMI
FSLQSNVGEVARADLEKNIEENKNDDACRAEDPPKVCDKECADIGGEAACWGPGPELCKECRNFHFNNVSCVETCDPATSFQY
ETKTGTGSHVLATARNCDSCHPECLGGCSGHGAAHCFECRNFVENGECVADCPLEKYPIREEKRCGACHEVCEADNGCTGGLP
LLGPGGCNTCSGLVVIQRDGSNRCLPTNVTECDQQHFWDDISIHESGGEYGLYAGHRVCKLCDEQCAGCVGLGPGKCRECLHY
MQNGECVGQCNSPQYAPNDHTGECERCSDQCRANGCFGKEPYECNSCPVYFYTNQANQTVCTDECPAHLGFKRELECVAACAE
MEFSDGGRDNNCRACHSECAQGCTGELRFHCLACKNVVSEGACLATCPAHSTPDANNQCIPGDPKKRAPAISKGLVFALLGAG
VLVVLVLGCICMIFVARYRKLKSRHTHHELQGRSRLYTDSPDELPNTPTGVEPNRAKMRIVQKHELRIMGTLGSGAFGTVYKG
IWCPEDDPKVQVPVAIKVLNDSGSESNKNFLDEAHFMASVAHPCLVRLLAICMTEQLQLITQLMPHGNLHDYIRQYGPAMSSK
NLFLWAKQIAEGMEYLQTKHIVHRDLAARNVLVHSTKRVRITDFGLASMLKYGEEEVKFEGGKLPLKWLALESIEHRVFTHAS
DVWSLGVTLWELFTFGEKPYNEVKATDMIRVLKEGERLPQPAGATLDLFVIMLKCWLVDPKSRLTFTEIKDELARMLEDADRY
FEVKNDGTIKPPSPIDDKELMSGFFESQLSTGRTLVPDAEEYIKLPTSSPQVSLAGTPTSNGPLGPDNTYVNTPPPRCTSRGS
NRSLGGGSAGGGSAAAPLTGPNGQPSSVFNYEHIPDHRTAAGTPAAVNNLYTLAPSPAPQIVPQGLEPTRKFNRDYYNSPPSS
AISPLQPNPAYRTSKPSPLSSDVRNSNSNYSPGSNPGTPVAANGNFFPITAFPNNNNASSVV
>Isodiametra pulchra EGFR-4
TGYLFIFSNVVPEVRLDNLEIIRGNTLFQDKYSVYIAKNSVSSSKVNLKRVILPKLREVSRGDIWVLENFGLCYMDKVNWTDT
FLKPQNLAEAIRDNNQTCGEEQCSPSCESGYCYSNDADACQTLDRVTCHPNCNGRRCTGPGAGDCCHEQCAAGCSAPNDQTKC
NFCQSFINGDECVQHCPPLSVYDPLTQQYRPNPEGKYSLGYTCVDRCPSYMLIEGNACVKHCGEGKFNNSNKCEPCPEEPCPK
VCVTYSETSENEMRLGMFNYSSLVNCTVIEGNLLINDQARLAIGEAGFHNFLHQLSTIKEVTGKLELLDLPQVANFSFFSSLH
TVGSRTQDGKPVLLIANNPDLEFLGLTALRQIKHSDAATDPVVLKNNPNLCYINYPMFRNITVNSANMHDQESFFKNRETADH
SDEPVYTLLPDQQIKCTNDGNDCDDECAAVGGEQACWGPGPDMCLYCRHFQLNNRTCVPTCDQQTSFVWGNNQCEHCHPQCAG
PCTGRGADQCEECRNFVLGSECVAECPANMYPDPATRTCLPCHRMCRNNTEGVAQWGCSGPGAFVGEGGCAGCDGVVVFADGS
NRCEAISANQCAPGFFYDKIPTGAVDNIFSGSQMCKECHEECDECTGTGPSKCIACKHVKFDEDCVSQCPSDTVLLRDECVKC
HSQCDATRSCGGPLDTDCNQCMRFFIRLDNQPDTVRCVGSCPKELPLRNETQCVAKCPKMTFKSSEATCKPCDEQCADGCTGD
YRHHCFACRNFQLGRDCVEKCPGGFKIKDKICLQPAHQEEGVTENAWFLTGVIGVAGVVVFGLLGTVLWCWIGRQRNRKAAMY
MSTFDDIPDRSFNVGDQPEGPFRSEHILRVIKKDELVLANPIGSGAFGTVYRGVWLPENDNLKQCPVAIKILNELTPGSQQSF
IEEAKNMAMVRHRCLVSVVAVCMAENPLLITPLMHHGCLIDYMKRTSFIKGSTQLLWCAQIAEGMTYLESRQIVHRDLAARNV
LVENKNQVKISDFGLSQIMRDGTEIQHSGGKMPTKWLAIESLKDLVFSHKSDVWSFGVTCWEIFTQGEKPYADIPNQDMKALL
LSGERLPQPKNCTIELYVLVLKCWMLEPTARPSFEKLQEEFTRMYKDGGYIADYRRDTSAEPGTPGVVLTSQPSFIDGNRDVV
PIEQYLHQEAEKERAIDHMETKYQPLEADSGVGCSPINYENMSPSPYPAMTETQFMGPNTVGSVDYSEAVQSEDEGYPPNHPV
HSTYPRATTQASNDPHNYMNTGYSPLKNGAEKRVDGHYARMKPNPDNDPSKPRATLPMNSKKAKHSRANSDPDRTLLANGRTP
PNGSGLYGGSLDRPSHRKKQKSFNFDRIEESIS
>Isodiametra pulchra EGFR-1
LLLNRYMMIILFTLLCSLRGIAGQSAEVFASTGDLLESFDISDCNTELQPSNWPGRELNVGQSGFHFRVEGLSMSYSIECKLR
FTSQVLRRSNVHGFIKLELPPMHFFDQVAQCDREGVRQFVKVFVDGGEEVVAEQTFQEQDLICYALFHFDSNNGEVDVVRYKN
PQTDDGLLLMEPLYKERFEFLAVQDCYSINNPKSKTEDDVVTHPRSFMETDEIFSGSSCYINIDMRDEDCATVLDFNLPPFKG
KVQCESFSFTAWSGDELISTYDCSTVTDSYAPAWVIDTDKAVVKVTLGDFEQGTIARPYTRDSMQIAVKCMHHVPDYVVCSEE
CADDPSGKYLTPCWSLRATDCYQCRYARDEDMCVSTCTSGKFENNKVCMECHPNCQPRTTCKGTDEADCNKLCKTGQARVSLE
DGRVGCDLDCPIEKPYSIFNTTGTEYEPDDEFLEDVLDPLCVSACPANQIPVPLQGTNFTFCRPQGNKDDGVNYLLVILLPAA
LSAMFVIFVVLILWGIKKHKKQIQRKKEHQKDFYIGDVKASKGVANRGKLRILERDEILLDQVVGQGAFGKVYSAIWRPKEEQ
GQVQLPVAVKQLHSAHTAAEVELLEEAALMASVDCENLVRLVAICMSEPMMLITKFMVHGCAIPFIKKHKQEMTSYQFMEWSR
QLANGMAHLEARGMIHRDLAGRNVLVESPRLIRISDFGLSKILDAEKDQETFTPDDDKLPIRWLAKECLVNKTFSHKSDVWAY
GVTLWEFYTYGMRPYNDQTLQQVREIVIKGRRLVQPPTCSLDLYTILLKCWMGVPENRPSFVELEEWFAQNRSHASKYFFTEK
KDEPVGADGKKMQQSNKVRLGNTGSSSLGSNEKATVEADRMPRDRVARAEHTGDTSIPNPETNSDDYLVPQSNFAVHIPDEEY
MRPIPRQNTNLTSVESEDIPNISLESSAARAELQKHFPQMNPDDFHAMNKRIDYQTPASEKEDYDDNVTSNYLGMDGGDRGST
EYMDRKDKVRVSKRWKQKPNVADNPAAHRGDEPTELAPKPNPRRQGGGQQQQQGRTPSGQTSATIAPADSMRRHMPTESTV
>Isodiametra pulchra EGFR-3
ASVDCENLLRLVAVCLGEPMMLITKFMVYGAALPFIKQHRDDMTSYQFMEWSRQLANGMAHLESKGIVHRDLAARNVLVESPR
LIRISDFGLSKLLDIDKNETSIRTQNTKLPVRWLAKECLTEQLFTHKSDVWAYGVTLWEFYTYGARPYGSHTLEEVRELVIRG
RRLVQPPTCSLDLYTVMLKCWMGQAENRISFTELQEWFAANRCHASKYFYPEKRDVPLEADDVRTAAKISLGGASSSGSAAPM
LPSSSYNGTPTTMENSLGKPQPSKPQYIECRQSGLVVDLNDYCPTPKQRLSKCVEPDDEDYMMPISQTNYPDYPEDEDDIRDM
SFNDSFTREQLQRAFPQFKPDNFAQASNVVDGGRLARPDRIVGSSSNNSLQSLQSNGMPKSVADSEV
>Isodiametra pulchra EGFR-2
MSAAAAVRLAAACVLLVPTMPLLSHNSPTMDFISVTDDTGRKFPVPLGSLAEPYDLSVGTPYRFWVTNMRYMHSKRFVFNLRP
PPFGTDRSYSVFLRATLPLAFFHNSYQTVDVMESCRAQAHLLFQVNASSGAEYDTPVTPKVYALQSGYCTAIFHFWTSGPYRF
VRDFLPAPTSQLPFHLGPLDSTMDVTLVANCPPESAPRRIDTTPGAVNSPSTVFPGGKYPVGLQCHYVIDYTRAGDCWIAVEQ
NARIVHHCDVWFKEMWHWGGRGLALDSQVDRMESMVVREDNGYLVVQFGMDWLEPSVSGEVRQCGSKIDLLIYCIHTEPEAER
RMDGHGHEYVCDEECTAPPVSNRSSRAQPLCWGPLATQCVACRHVRDQGTCIAQCGHAKYSDGGGQCRDCHPRCVSRSCVAPR
EQDCDRVCEVGKVRTDKNMTTHTSSTEGIVYIRCADQCPDDRPFAKLHPRSTGMQCHENATLADVEARYDLFCAESCPRGWTQ
TRRQGYILCEEDCNRQAHAYDHSFELVLFLILFGSAVALIMTAIVIVIVIVRNKMRKARKMRLKRIHDYYLNVNPQYKGEVHA

GHLRDIDIDELEMVRELGRGAFGTVREAIWRPKGDIDVEIRVAVKQLHSRGTSDEELLTEAASMASVDCEHLLRLVAVCMTEP
MLMVTAFMPHGGALDFIRHHQETMNAYQFIDWSRQLANGMAHLEAHGMLHRDLAARNVLIESPRHIRISDFGLSKFLDVENSE
THITGDERLDMTRLPVRWLAKECLLSRTFSHKSDVWAYGVTLWEFYTYGKKPY
>Convolutriloba macropyga EGFR-3
MNSFKITLYQITCNLLLHWAVIQLVLCAVQASSSVPYLECTGTNNKFSNLEADLSGSDGTHYMRLRHLYTKNVTESSSSRRTK
VRKCTHINGNLEITNIHEGTDRALDGLVYSFDFLEEVREVSGYLMIYDVELTRLQLPNLVIIRGNTLLTNMQQKQWGFYVQSL
TEHTQNEKGLQELSMPKLKEISKGSVYIANNPELCYINLVNFSDILQKEDNSDPLYSIDIVDSGDSDSCNKLREEGKLICGAH
VSSPTTLSPASAPHALSPTPNNRNITDSPPLVNTTVYISSVVDGVTEVLEPVCKYGHCWGPREEDCQNLTRLVCSPKCQKKGS
STSLRCFGPQDSECCDEDCAAGCWGDFRYECHACRVLENENECVTMCPGISYDPNYKLNIDSKKIRYAYHYKCLSECPDGTYI
NDLDSFGKTCTSKCSADQYLDGNRCVQCEKGRCERGCEIPNLEGKCDKSNPIVPFEHELSEGDLCTKLVGDLILGHSFWIGDP
WCGIRPFDLSKLHLFKSVREITGQLKVTASPLVNLSFLSSLEVIGGQGLTGGGSFVIGDNRKMTHLGLASLKQVKRMPQGETM
SITGDRLCYVNEQMLRDLIVPRPGTSEVSTIMNIGEVASAEDCDNLPKCDKQCAKVGGKQACWGPGPDMCFKCRNYDFNNKTC
VETCDPEKAFVDGKMCESCHEECSGGCQGRGPENCSKCVNFENDGMCVKQCPVGMFALSDTKMCRPCPPTCELDRGCTGNKSI
LGDGGCNRCDGMVMIHNVNLDAPVHSMLWGRNNTCEPPPKVEYECPENYFYDRLGVLETSIFAQQMLCIACDPECIGGCSGSG
PTKCKSCKNFLQGNVCVPDCGYSYASVSSTGKCERCNEQCDPREGCFGGEAYQCNACKHSFYYIQSTTTSENKSAERVKNCVI
QCPDRYPFLIPNGNECVSQCKDNQYNDSANVCKPCHSECLANCTGDLRSECVGGCLHFEYEGGCYRECPINTRQSPKNNKMCV
AVAPTGLLLSALFSGKRRVPVLIGMGVVTCLLIVSLVFYCKYRARKRNAIAEKLQREQKYLGMDMIPKDEPTPNTPTGIEPSK
GKMYIIQKHELKRIGPLGSGAFGTVYKGVWCPENDAKVQLPVAIKVLQDPVPGAEQTFIDEAQFMASVSHPCLLRLLAICITE
TPQLVTRLMPHGNLLDYIRKYGNTQSSRTFFLWGRQIAEGMEYLQSKRIVHRDLAARNVLVQSSRQVKITDFGLAQMLKHGEE
SVKFQGGKVPLKWLALESLTEKLFTYQSDVWSMGVTLWEIFTLGSRPYDQITGGVAEMVKVLQDGERLPQPPGATLDLFVVML
KCWLVEPKSRLTFTQIREEMTKMYEDADRYFEVQTDAMNKVDSPRDDQEFMRDFLLDQQNIMDPGHFPTERPFSKGSLDLARS
LVDPEDYLGLNNSTIPRGTLNSTTGRNSIHQQHLNQSYNHAPSSGSGATMDDTPPYINSPSASTTSTVIDTNRKNSRSKPFIP
PPRLPLHSPIENYPGSAGGPNRPVMPYYANSAGIHPGGSMGSGGSGGGGGMMQGQHSSTNSTGCSRGSDAENVFNYEHVPDQ
RFRQGYDPLNYSQLSSAITTPMSAVPPEYYNSPGGPEDPNVLSRSFNAGYHQPLTHAPNTVPPYYSSKSAGVPQLPHKLVLNH
NPGYNSNPQYPNFVPANSNYSPATDSNPATPINLNNNSSPFSGYSSVNPTKQNPNQVVSNIASHAGKNNSQDRNKNSNKNRNS
KNYQSMDNNASSFV
>Convolutriloba macropyga EGFR-2
MQSNRILAEASFCVTISIIFFSFWTTGASDATQKRVECEGTRNELSITGNHEEQYKSIVKRYSKNCTVVLGNLEITFVPEGRT
LNFLNTIREVTGYLLIYNNGVDKIKLDNLEIVRGKELYTIGGASYSVAMLQNKKTRLLVLPKLKEVTQGEIRIGYNENLRFQK
KVNWADIDSREHPEGQTPVYTDSNSKDDKDSKCHSSCASGYCWGPDASDCQELTKSPCDESCDGGRCFGPGPNQCCNDQCSAG
CTGKDADQCNLCKGFVDRDKCVESCPPLERYDPVNNRMEPNPNVRYSLGFECVAKCPHQMLIEGNACVKHCGDKRYNVSGTCE
QCPSDFCPKGCSTLKFAHQMRTGEYDYSQLINCTVIEGPLIISDRAKGWNNLNVFLKYFEAIEEITMELQVLDTPLTNLTFLS
RLKRIGSRMEDEMANFVVMNNYQLEFLGLKSFEELKHPKAKENPVTLTTNPKLCYVNFEMFRNITVHSEKMTDKVKFMPNLEE
RTAECKINGKQCDSECITMPANDEPACWGPGPQMCFKCRNFLMDNSTCVNDCDLDRAFVVSDGSRGGGKKCERCHEQCAGPCW
GRTAFECEMCKHFESVTGECVAQCSPDEYADPLTNKCKKCSPLCSTDPNLVGCSGPGEFVDDPGRKLTGGCFQCPGVTKFRDG
THRCDVPPGESSCPKDFFYDRISASVRGDIFSGSSICEQCNEECEGCDGRGPGNCIKCKHFEFRDTCVSLCPDHSFHRDESLV
CESCDRNCKSFQDGQEICTGTSPFECVECNWKYIMHNATSRECVESCDDAHPFVSEYRECVASCPDLTFPLNTTTSGPVCRRC
HSECLFGCKGDLDTDCLAGPPGVKGRRRCVHYELEEGGRCLEECDGDFEVTSQFTCKSIETNFIMVNAHIFFGGPLVALLVLS
MVAMCCYCRRKSISERKKQNQYEAELHMRELGDADSEMNSPMSTPDHVQEFPKLKVIKLEELLEFEEIGSGAFGTVTRGVWLP
PDDKKRQCPVAVKTLHDSSGLQQQNFIEEAKNMAMMRHRCLVSLVAICMTDKPRLITPLMPHGSLIDYMNQHHHMLATSMLLW
CKQIAEGMEYLQSKNVVHRDLAARNVLVETVRQVKISDFGLAKILKNGQATVEGGKFATKWLAIESLTCFEFSHATDVWSLGI
TVWEILTHGEKPYDDTPTVHMAEFLKNGGRLPQPPGCSLELYVNIMKCWLAEPQVRPSFTELKESFDNMLQDPSAYLSDYYDP
HPDDEYNGPMLVDTNIVYPTNGHANGHDSSSHPVPFVETSVLNSGDIQERTNVHVERYLDEHRRVEEVQSRYDDYSSSRIHHV
PGQSVPYENHYPQPTLDVEYGHFEGNGIYDPRLQRTYSQPHPVNHFQFPPTQPIEYSQATTLDHFEAKNRQFFAHPDMINRQN
STSSTNPMIISQPTQTFLPPPVVLRRMHSQPPLPPRLDIRRGSSFSGPMYDNMSSRNRNSGYSSINRNLNSPQKQNFKKDKDK
SSKTLSNLYKLISKSGSSSKSSQRGSKSSQKKPRLDDAQHQRARSEPMESLTIVNHQPNFGLNMNNFDINQASSMRNDPSFAV
GSKQMLTFRSPGQVRTVASGALNNGNGNSPLSSPSKMSAPKSPPHIMLPNSGDSFEAGGGGVPREYHV
>Convolutriloba macropyga EGFR-1
MAVMDILRFLLLTCMASISTQQSELAKVYHTNVKGTATQFKCSSTPKQLTYSPYLMVSNVIGVTTDCVFQADSFFITLWTEDK
PYTGILKVSMQLIVAMAIGEPTSTPQQLCENLFDIAVMVQISSEKEKEYDVTIRSQTVFLDGESSNCVGYYQFYSRSGKFRVT
KKGIPPSDVKPGKEMVLANDNYIISNFEYYELKFCSSEFKPMRLNHKQWVQNPIAVLDGNYPSGTECYYQVEPLDDDCHTVLH
FQQNPFELKEECETWAIEATFMHKNGSQVTERFDCSKIVDNAHVNPPWIIHHPSPLIRLRFGKPTGISVGSSLSSRANREFYR
LDRFELSADCLHTETISDDQLICSSECDNKVGEASLYIKPCWGRSSRECVSCQYAIDESQCVSQCPADKFRNSYGVCEACNAA
CMGDQSCLSPFASGCYRLCHKETPFRTNYVPGRRNIYMGYLPADPFTIKDCATRCDKGLAAMFYVNGTRFDMDEKYSTPTAAL
TNDLMNHMDITCTDKCPEGFKFADCVKDGPYQDMEWCCPVEEEGFATWELVLLFVLMPAAVGAFLVLGVVMIIYCWKKHKKTI
QRKEQALKDFYVGEGKSNIRGIANRGQLLIVEPEAIELKQVIGQGAFGCVYSALWRPPNENIEIPVAVKQLKNDPKAANQNTE
LLDEAKLMASVNHDNLVRLIAISMNDPMMLITKFMTHGGSLQFIVRHKDIMTSYQFMEWARQLAAGMEHLESLGMIHRDLAAR
NVLVEDPKRIRISDFGLSKLLDADKDEVDYQADDSKLPIRWLAKECLVGRLFSHKSDVWAYGVTLWEFFVYGLEKPYGKVDLQ
TVKDLVIRGRRLVQPQTCSLELYTQMLKCWMTVPDSRPSFTYLENWFAESRECASKYFFTKYRDNPLEQPAKLQSNIVNLSST
SNNSNEFATRGTAEADMKPRERVSSGGARGGVGVPNRAGAVGPVASGRRAQEQHQGDNSIPRRDESGSDEEYLVPQKPPSRPP
PPSQLPSSPPHDTSHFRQVEEDEYLRPVPMPRNANPAAPVPPHTSSVENDNSLGGIRDFSIETRENELQKYFPGMDVRNFDKA
HNMMEKDYDSSEPASNYKDINSENGLRNSSEDLEKNGADNMDRKEKVRISQRWKGKQKPRLAHQDSVTPQGDYTDVNTRQPYT
GAQGSRQPPPQAPSGGAGNSGTGQKIATADSMRRNLPAESSV
>Priapulus caudatus EGFR

ERERDRQTHIHIISISMSGVSGCVTLWLLWITALLSMTVLAEEWEPTFTRLPNEQVCPGTNLKLTRKSSDDEHYRSLRKQFEN
CTFINGNLEITFIEDPSRDLSFLQHIREVTGYVLIAFVYTERIPLHNLQVIRGQSLYGHPDQGDEEEKYSLYVALNYDKANFN
NKHEVGLKSIGFEKLGEILTGDVSFIDNPYLCYIDTIEWKDIVAGSVTMINNSVHQSCPACHPDCELRCWGPERNDCQTLTKT
TCAEQCSRGRCFGPDPHDCCHQSCAAGCRGPLDTDCFACKMFNNSGTCVDKCPSLEIYDLSEFQLKDNPDGKYTFGSTCVNEC
PSHLLKQKSHGACVRQCSEGYRADDNNFCVACVGPCPKACDGGTVNSMNIASFEGCTIIKGNINILDSAFVEYTEFIQNASYP
AGTMIVTVPPLHPRQLLAFQAVREITGYLNIQGSHVDLRNLSAFSNLEYIKGRELYGDKIALNIQTIEHLESLDLKKLRSVQN
GGIFINGNTKLCYVGTVNFSLLIGDTSKQELSIKNNKPTAQCMLDGRMCHPECSSDGCWGSEDDACLKCAHYKLEPEGLCMHS
CDVRTGLYNSSETICSYCHPECSGTCSGPGADKCEQCKNVKDGPYCLDECPLVKYLNSTSGVCMPCADCKLGCTGPETWLGEG
GCNACHLAIETTGRETEWECLPRAMSHSCPAGTYYTLVRYDRHLLLQDDNTEMTACRKCHPMCESCDGPGQICTCKYYRDEAT
GLCESSCPEMTYLRNDSNVCSLCSFECDRACRGPTSLDCLSCRNMKVYLDEEESQEEEAKDRAFNCTAECPPDRPYETRRPEE
GNVCSARRSTGPSVPIIVGSVLGIILVIGIILVCFVYQCRKKAQSEEATMKMTIRMTGIDDSVPLTPTNATPNMSRLQIIKEA
DLRKGGILGYGAFGTVYQGVWIPERDTVKIPVAIKVLREGTSADSNKEMIEEAQVMASVEHPNLVRLLGICLAAQMTLVTPLI
PLGCLLVYYVRSNKDKIGSRPLLNWCMQIAKGMQYLEERRLVHRDLAARNVLVQSPGLVKITDFGLAKLLDINEDEYKAAGGKM
PIKWMALECIQHRIFTHKSDVWSYGVAIWELFTYGERPYESVSARDVPDLLEKGERLPQPNICTIDIYMIMIKCWMLDSESRP
SFKELGADFAKMARDPARYLVVQGDDLMRLPSFTPQDNHEFMRGLSVMEDGATGGGQRRLVDAEEYLQPTPAAAIAAATMPPE
QQSPPAASPPTSPEHEPFESPSKKKLLATYEEEAEGEDPHRRAREKKYAHLEKRSPRPREREDSIAGRYSSDPLEARLGEADI
AEEPSTSAGHKLPNSIAMKYGGGGGHPHLRLQVDEDDYLLPNPPDQPPRSNMADAYLDLMNPEDSPIDYADVTPAVKNAKERP
QFKPVPVAVENPEYMDSATNQEEAERLRATGGSTSSEASAADHAYYNDFDSMQKRDRRLIKPLTKNETSV
>Halicryptus spinulosus EGFR
MYASAKVGCIIVIIAVVNLLFIEETDAENVFENGVEGDIVCPGTNKKLSRKAGERDHYELLRKQFENCTFVNGNLELTFVENA
NWDLSFLQNIREVTGYVLIVFVFTEHIPLNNLQIIRGQTLYSHPDDGVGKYSLYVAKNGDVGSNVGLRSLGLEKLGEILNGEV
AFINNPYLCHVDTIAWDDLITGPMRGPPQIDIAAPGSCAACHPDCNGHCWGPRKTQCQSLTKLNCAEQCRRGRCYGPNALDCC
HQSCAAGCSGPLDTDCYACRNFNNSGTCVDKCPPLEIYIPNEFKLSSNPYGKYTYGSTCINECPAHLLKDRSHGACVRECSPG
YMADDGNFCVSCIGPCPKACDGGEVTAANIEQFRDCTVINGVINILDKAFKGYVEFIQNNSYPDGIHLLNIPPLHPDELQIFK
TVKEITGYLNVQGSHEQLENLSAFSNLETIRGRELYADKIALNVQGITELQSLGLKKLTTVQNGGIFMHLNTKLCYASNINFD
LLLRDKEKQKISISRNRAAADCETEGKVCHPQCSADGCWGPEDSDCLSCNNYKLGNTCLENCTVQDGIYVLGPMKCDYCHPQC
EGTCFGPGPEHCEKCRHAKDGPFCIAECPEIKYLNKTSGECEHCGKDCVGGCTGPENFPGKGGCKSCNMAVEAPPSSSKRWDY
CLPAYSKCPQGYFYTRVILDAENPMAVCRRCHEQCTICEGTGPSCIKCRNFRDQATRMCTQECPYMTYLETNTSTLCKSCSSQ
CAEGCQGPTALDCVTCRYMKVYLNKEETEFNCTEECPDYKPHEIREKGHGNVCREKLPARRLTVVWTVVIVIAVVAVLTAMII
VLVWLCLRRQKSKQATAEMTMKMTGMDDSLPLTPTNATPNMSRLRVIKEADLRKGGILGYGAFGTVYKGVWFPEGEEIKIPVA
IKVLREGSSTEGNKEMVEEAHVMASVDHPNLVRLLGVCMSAQMTLVAPLIPLGCLLDYVRMNKPKIGSRPLLNWCTQIAKGME
YLEQRRLVHRDLAARNVLVQTPGKVKITDFGLAKLLDINEEEYKAAGGKMPIKWMALECIQHRRFTHKSDVWSYGVTIWELFT
YGERPYDQVSARDVPELLEKGERLYQPHICTIDVYMILIKCWMLDADSRPSFKDLNAEFSKMARDPARYLVIEGDNLMRLPQY
TQEDNREFLNSISVVDEAMPKRMVEAEEYLQPMGTDVPDYPPPSSPPTSPEHGAFDSPSKKKLLPVQEEATGGLKIREKKYAN
LEKRSPRPRDRESSAGRYSSDPLKALAGDSEDNIVPEKLPKQVAMKYGDLKVNLPVDEDDYLLPNSPNQPPRSQVAYLDLLSV
ADQPGRLDQLEEKSPPNYLSKLPAVDNPEYINSTTSEGKAAEADRLRMSGGSTSSEYSVSEHPYYNDCDVGRREKDSHLLKPL
KSAKSETAV
>Caenorhabditis elegans LET-23
MRYPPSIGSILLIIPIFLTFFGNSNAQLWKRCVSPQDCLCSGTTNGISRYGTGNILEDLETMYRGCRRVYGNLEITWIEANEI
KKWRESTNSTVDPKNEDSPLKSINFFDNLEEIRGSLIIYRANIQKISFPRLRVIYGDEVFHDNALYIHKNDKVHEVVMRELRV
IRNGSVTIQDNPKMCYIGDKIDWKELLYDPDVQKVETTNSHQHCYQNGKSMAKCHESCNDKCWGSGDNDCQRVYRSVCPKSCS
QCFYSNSTSSYECCDSACLGGCTGHGPKNCIACSKYELDGICIETCPSRKIFNHKTGRLVFNPDGRYQNGNHCVKECPPELLI
ENDVCVRHCSDGHHYDATKDVRECEKCRSSSCPKICTVDGHLTNETLKNLEGCEQIDGHLIIEHAFTYEQLKVLETVKIVSEY
ITIVQQNFYDLKFLKNLQIIEGRKLHNVRWALAIYQCDDLEELSLNSLKLIKTGAVLIMKNHRLCYVSKIDWSSIITSKGKDN
KPSLAIAENRDSKLCETEQRVCDKNCNKRGCWGKEPEDCLECKTWKSVGTCVEKCDTKGFLRNQTSMKCERCSPECETCNGLG
ELDCLTCRHKTLYNSDFGNRMECVHDCPVSHFPTQKNVCEKCHPTCYDNGCTGPDSNLGYGGCKQCKYAVKYENDTIFCLQSS
GMNNVCVENDLPNYYISTYDTEGVIETHCEKCSISCKTCSSAGRNVVQNKCVCKHVEYQPNPSERICMDQCPVNSFMVPDTNN
TVCKKCHHECDQNYHCANGQSTGCQKCKNFTVFKGDIAQCVSECPKNLPFSNPANGECLDYDIASRQRKTRMVIIGSVLFGFA
VMFLFILLVYWRCQRIGKKLKIAEMVDMPELTPIDASVRPNMSRICLIPSSELQTKLDKKLGAGAFGTVFAGIYYPKRAKNVK
IPVAIKVFQTDQSQTDEMLEEATNMFRLRHDNLLKIIGFCMHDDGLKIVTIYRPLGNLQNFLKLHKENLGAREQVLYCYQIAS
GMQYLEKQRVVHRDLATRNVLVKKFNHVEITDFGLSKILKHDADSITIKSGKVAIKWLAIEIFSKHCYTHASDVWAFGVTCWE
IITFGQSPYQGMSTDSIHNFLKDGNRLSQPPNCSQDLYQELLRCWMADPKSRPGFEILYERFKEFCKVPQLFLENSNKISESD
LSAEERFQTERIREMFDGNIDPQMYFDQGSLPSMPSSPTSMATFTIPHGDLMNRMQSVNSSRYKTEPFDYGSTAQEDNSYLIP
KTKEVQQSAVLYTAVTNEDGQTELSPSNGDYYNQPNTPSSSSGYYNEPHLKTKKPETSEEAEAVQYENEEVSQKETCL
>Drosophila melanogaster DER
MLLRRRNGPCPFPLLLLLLAHCICIWPASAARDRYARQNNRQRHQDIDRDRDRDRFLYRSSSAQNRQRGGANFALGLGANGVT
IPTSLEDKNKNEFVKGKICIGTKSRLSVPSNKEHHYRNLRDRYTNCTYVDGNLELTWLPNENLDLSFLDNIREVTGYILISHV
DVKKVVFPKLQIIRGRTLFSLSVEEEKYALFVTYSKMYTLEIPDLRDVLNGQVGFHNNYNLCHMRTIQWSEIVSNGTDAYYNY
DFTAPERECPKCHESCTHGCWGEGPKNCQKFSKLTCSPQCAGGRCYGPKPRECCHLFCAGGCTGPTQKDCIACKNFFDEGVCK
EECPPMRKYNPTTYVLETNPEGKYAYGATCVKECPGHLLRDNGACVRSCPQDKMDKGGECVPCNGPCPKTCPGVTVLHAGNID
SFRNCTVIDGNIRILDQTFSGFQDVYANYTMGPRYIPLDPERLEVFSTVKEITGYLNIEGTHPQFRNLSYFRNLETIHGRQLM
ESMFAALAIVKSSLYSLEMRNLKQISSGSVVIQHNRDLCYVSNIRWPAIQKEPEQKVWVNENLRADLCEKNGTICSDQCNEDG
CWGAGTDQCLTCKNFNFNGTCIADCGYISNAYKFDNRTCKICHPECRTCNGAGADHCQECVHVRDGQHCVSECPKNKYNDRGV
CRECHATCDGCTGPKDTIGIGACTTCNLAIINNDATVKRCLLKDDKCPDGYFWEYVHPQEQGSLKPLAGRAVCRKCHPLCELC

TNYGYHEQVCSKCTHYKRREQCETECPADHYTDEEQRECFQCHPECNGCTGPGADDCKSCRNFKLFDANETGPYVNSTMFNCT
SKCPLEMRHVNYQYTAIGPYCAASPPRSSKITANLDVNMIFIITGAVLVPTICILCVVTYICRQKQKAKKETVKMTMALSGCE
DSEPLRPSNIGANLCKLRIVKDAELRKGGVLGMGAFGRVYKGVWVPEGENVKIPVAIKELLKSTGAESSEEFLREAYIMASVE
HVNLLKLLAVCMSSQMMLITQLMPLGCLLDYVRNNRDKIGSKALLNWSTQIAKGMSYLEEKRLVHRDLAARNVLVQTPSLVKI
TDFGLAKLLSSDSNEYKAAGGKMPIKWLALECIRNRVFTSKSDVWAFGVTIWELLTFGQRPHENIPAKDIPDLIEVGLKLEQP
EICSLDIYCTLLSCWHLDAAMRPTFKQLTTVFAEFARDPGRYLAIPGDKFTRLPAYTSQDEKDLIRKLAPTTDGSEAIAEPDD
YLQPKAAPGPSHRTDCTDEIPKLNRYCKDPSNKNSSTGDDETDSSAREVGVGNLRLDLPVDEDDYLMPTCQPGPNNNNNINNP
NQNNMAAVGVAAGYMDLIGVPVSVDNPEYLLNAQTLGVGESPIPTQTIGIPVMGVPGTMEVKVPMPGSEPTSSDHEYYNDTQR
ELQPLHRNRNTETRV
>Tribolium castaneum EGFR
MGIRHLLVLFVVVLLVLVVCDCRQHYNRNRRRGGAKDKALALHHQENEFHNKGKTQLSLPGVRPVPYSRPGKFSEKKGQQRAT
STDNNNKPIIAQQPVLLRHLPAVICINGYRFCNGTVSRDPGNGLRPKDLIANVNTVYLLCIFVVNRLIPIFLPVCIGTNGRMS
VPSNREHHYRNLKDRYTNCTYVDGNLELTWLQDENLDLSFLQYIREVTGYVLISHVDIKRIVLPRLQIIRGRTLFKMNVRNEE
FALLVILSKMYTLELPALRDVLIGNVGVFNNYNLCHFKTINWKEIITDPKSKYVFVYNFTSPERDCPPCHKNCEKGCWGEGEE
NCQKFSKENCSPQCYQGRCFGPNPRECCHLFCAGGCTGPKQSDCIACRNFYDDGVCTQECPPMKIYSPITYSWQDNPNGKYAY
GATCVKNCPEHLLKDNGACVRSCPPDKKAHEGACVPCNGPCPKTCRVDTFIHSGNIDTFKGCTVIEGNILILQNTFEGYQHFY
PNYTFGARYPRMHPDRLEVFSTLKEVTGHINVQAYHSDFTNLSYFRNLEVIGGRSLSDYFTSLYIVKSSLKSLELRSLKRLNA
GTVAILENTHLCFADNINWDKIRRSHEHVVMISNNSDPRLCKGNNLVCDSECSRDGCWGAGPDQCLSCAHFQLENESRCIANC
SVLPGIYQDGPNICKRCHEECGSSCTGPGADKCVTCKRFQDGPYCVPSCPTNKYHENGGCNSCDRAIMNETDSMVYCLGSNES
CPDGYFNEYLGPQEKESLKSLAGSALCRKCHPRCKICSGFGFHELVCQKCTHYKKDEQCEDECRSGDYADEEVQECKPCDPEC
KGCTGPGPENCISCENFKVFDDGYVSENSSFRCVSSCPPEFPHRVFPDNAFSYCSDKPLNLPISGFVDNNYTVVIATGITSAI
IIIIIIIILVTWFFRKKVKAEESTLKMTMALTGLEDNEPLRQTNVSPNCKKLRTIKETEIRRGDILGYGAFGTVYKGVWILED
ENNSKIPVAIKVLRDGSTSSKEFLSEAFIMASVEHPNLLQLLGVCMTSEIMLITQLMPLGCLLDFVRKNRDRIGAKALLNWST
QIAKGMAYLEEKRLVHRDLAARNVLVQTHSCVKITDFGLAKLLDVDEDEYKAAGGKMPIKWLALECIQHRIFTHKSDVWAFGV
TIWELLTFGERPYDNVPARDVPELLEKGERLPQPEGCSIDVYMILVKCWILDAESRPSFKELAEDFTKMARDPCRYLAITADR
FARNRNNAQDDRKLVHTLASTLETSASQSECDEYLQPKSRAPLRPVGWMSSVSPSPTPDKYWKSASQTNSDGSCNPQYQNHLN
RKLLKYPPSTASDTLKMRDDMSSDEYDSGPSKAQLGSLKLDLPVDEDDYLMPSPQQTQGASAYVDLIDSKNSDTSNQSVFRSL
DFYKSNIDNPEYLMNNEAAPSQTVGLPQVPRSSEEESDHEYYNDFDRLKRELQPLQRNKDGAIV
>Phoronopsis harmeri EGFR
GSGAFGTVYKGVWIPDGENVKIPVAIKVLQEGTSPNQNKELLEEARVMASVEHIYCVRILAVCMTAQMMLVTQLMPLGCLLDY
VRKHRENIGSKTLLNWCTQIAKGMSYLEGRGIVHRDLAARNVLVQNSNMVKITDFGLAKLLDFEENEYHAAGGKMPIKWLALE
CIQHRVFTHKTDVWSFGVTVWELFTYGQKPYENVRAKDVPELLEKGERLVQPTICTIDVYMIMIKCWMLDADSRPSFLELADE
FAKMARDPGRYLVIQGDRLMRLPSQSYDAHDVMRSLSVAVDGPEEILDADEYLQPSEYQRDAEPTSPVSPFSPPYGYGRNSLS
KKPL
>Terebratalia transversa EGFR
MISRRLPWTSHMVSWNFILCVFILVIFRSTVAVNIPRKVCRGTNLQLSYNAESLSVRHMNYKRRYPNCTYIDGNLEIVQLTGD
LSGYDWSFLSTIKEVTGYVLIVNFHGDTLPLTSLTVIRGNFRFENKGNEYSLYVANNNLRELGFKNLREIMNGDVMIGNNDNL
CFQQKVNWNKILTNTGYKQKISFGTGASNCVECHTSCPYCWGAGQDMCQTIIPCAEQCNGHCYGPGVHDCCNNECAAGCDGPR
RTDCVACKNFRNDNECVEQCPLPEIYDPKTFEFVRNKNAKFSYGSICVKKCPDNYLQYKAACVKVCPPEYEVKDGKCQKCITC
VTKKCNGTKLHNDPNANVDYWLTNKNIKNFQNCSVVQGSLIMNLFTFNDDTAKNTTAITRDQLEMLSSITEITGYLQIHDEPK
DKPFIDLSFLRNLKTISGRTTEKTAGTETIALEVRLGDEKTGLSFTSLGLTSLKKVEGGIVLISNNPKLCYANTVSWNHIAPH
QKNIIQNNAPVNECVNAGEVCDRQCENGCWGKGSNKCVNCRNKEIYLENQCVDMCHRDAKLYEPENDIKTCKRCDPECTNSCT
GEGNSKCDSCLNVLDGFRCKAECPKMKYQDANNTCQECHSTCRDEHGCTGPSEFVGPGGCNACELALYRKRKGVESLECLPLQ
TTECPRGWYIEAVKTRINDTSITQRMAGKSICKKCDEECLACTGPGPTQCTVCGHYEQHGTNIKLCVYQCPPLYYEDSKNCFP
CNSQCRKGCNGGKSSDCFNCLNVKNYLPPEDPVEIDGVTVEAKARFNCTEECPDEQMSKILEEETEDKYCGIKAEASIGVTKD
EYIGIGVGSGVAMFMLVVFVAVICYRRTKSKEKQLKLTSKMYGTDEGEPLTPTDAKPDLTTMRLIKESEIRRGGIIGSGAFGT
VYKGVWIPEGENVKIPVAIKVLQEATSNQNKELLEEARVMFSVVHPCCVRVLAVCLTAQMMLVTQLMPLGCLLDYVRKNKNNI
GSKALLNWCTQIAKGMDYLEGRGIVHRDMAARNVLVQSPGQVKITDFGLAKLLDYNEAEYHAAGGKMPIKWLALESIQHRLFT
HKTDVWSFGITVWELFTYGKKPYENVRARDIPDLLEKGERLPQPGICTIDVYMIMIKCWMLDAESRPRFKELAEEFAKMARDP
GRYLVIEGDKLMRLPSHTYDTKDLIRTMSIANEGPEEVMDAEDYLQPQSTTDDGDDIDGIDGAAGVSPRDRMVKKPLLGRERY
YSDRLRSDNSDENEYPSADRRRRDRKYGHLETAGNSRERGNSVNSRYSSDPCKLVDLKEREDGFGSEEEGVHFRDRHHIMYKA
PRFQYPITDEDDYLQPRSTLPATYIDVTDGPPVTGEHEYTNSPPSSKTQHCAVNFENPEYFDSISDAKTYRQPYSDHLKQNRD
NRNSSGNSDRISAASSGSEHNNNGELTPPPTYNEYYNDLNKIIANKNKLHERQNSIEKPPLNDVVFIESGSATQV
>Novocrania anomala EGFR
MNPRAPMCLLLRLLAGMCIYSISQVCSEQICTGTSTRLSITGDSFHRYTAAKERYTNCTYVDGNVELVFLDDPETRYDLRFLK
DIKEVTGYVLIVAVYAKYLPMENLQVIRGRTLYPHNGNNYSLYVALNSPRQTASIGLRELHFFKLNEIMKGRVYFKDNHLLCH
ETTINWVAIISGKNGGVDLEESPKQRTCGHCHSSCNNGKCWGPGPDMCQKVISCANQCDRSCYGKGPSECCHAQCAAGCTGPT
NRNCTVCRNFLNEGACVPFCPQPEVYDENEYKFVPNPHFKYTYGSLCVKKCPVHLLEDRRGCVKSCPPGSTAQDNKCVKCDGP
CQKECVGGKGTEFVNSRNIDLYKGCTVISGNLKILSPTFEGDEFNNISGMPVSALEVLSDVQQVNGFVQIQASESVNLTSLRF
LRNLQIIRGRNLHQQSTALDIINTQSLESLDLSSLKSVANGIVWIQDNRNLCYAHTIAWKSIVNHAEVGNNNNNTRCEKLNKT
CSDQCTVSDGCWGPGRDSCLKCRYKRLGKTCIPTCDPALRLYEVGNNECGQCHDECANNCTGPGADQCDSCKRYKDGPYCTDT
CPADKKYGDNKTKTCEKCHEFCTEGCTGPGNFIGPGGCIMCANSIWNETTRRPEPCQSPLSEMCPESYFALIIYKSHWLAKTL
NMAGKLMCKACDDVCLSCYGQSYTECLPGKCRYFRDAQLDFCVPKCPTDQYNTTNSTLCQTCHENCRNGCFGPNSTHCHNCRN
RKVFLDEEEKQFNCVTKCPAEKSVPSREGGDDVCRVEEVVLTAGSIAGISVGAVTVLGAALALFACMCYRRTKSKENALRLTS

KMFGMEENEPLTPTEAKPDLSKLRLIKESEIRKGGIIGSGAFGTVYKGVWIPEGENVKIPVAIKVLQEGTPNQNKELLEEARV
MCSVEHPCCVRILAVCMTAQMMLITQLMPLGALLDYVRKNKANIGSKALLNWCAQIAKGMTYLEDKGIVHRDLAARNVLVQNP
GQVKITDFGLAKLLDYNEAEYHAAGGKMPIKWLALECIQHRIFTHKTDVWSYGVTIWELFTYGQRPYENVRARDVPDLLEKGE
RLPQPTVCTIDVYMIMIKCWMLDAESRPGFSELAEEFSKMARDPGRYLVIQGDRLMRLPSHSYDTRDLIRSISGSTEGPEEIV
EAEDYLMPRTMTEDEVIVSDNELPQSGNSFKKPLLNQPSPYDERIRGANGEAGGDRNYSKREKKYGHLEAAKTGRGRGDSVSS
RYSSDPCKVLVDMKEREDEVDGDGLPKYPQLPIDENDYLQPKSSSRSPYMDLIDGVTGQFAVADYENMMANNKPYLKEMVAPQ
HLDNPEYFA
>Membranipora membranacea EGFR-1
MDIFTVYSIIISGIIYYSHGFRVMDTCQGTNAGMSRSNDDSENKLRFLQNRYGGCQYVDGNLEIVGLDSEAFYNADLSFLNSI
REVTGYVLIANVYVQNLTLENLTIIRGDKLFRPPSNGAVVPADQGSQESQRKEQGYALFVSLNYKKSSDSIGMRLLGLRSLTE
ISKGDVFFENNNLLCYATSDIYWSDIFTNTSIQKVSFIHDSVRNAQPCKPECHESCEKTLPQKNPLTGVTTEITRRFCWGPGP
KNCQNMNKVTCAKQCNGRCFGSSLTECCNPQCAGGCNGPLKTDCWGCKMFYNEGGCVPFCPYAQIYDGTTFKMVSNPDKKFAF
GRLCLPSCPDNFLEHQGGCVKSCPENFIAKPKVDVDGKKMGDILKVCEKCDGPCPKPCKIDWTGSGADYLNSANIHELEGCTS
IDGHLRILDYTLSGDHWKNISALSLNDLDKLKSIRQISEYLMVQHLKNVTNLGFLSNLETILGREGDTYGATLNVMANQDLEY
LGLSSLKKVYGGHIYLHLNPKLCYVQNMRWKKLFLSKTLVSQRIFMDGNANDTNICVRQNKTCHEECRNGCWGPGPHMCISCM
NYRYSNECLSDCSRPMIYGKPDSSKCFDCDPECKSSCTGKTADMCDDCRNLKDGPFCVAGCPIAKFADDNKLCQPCHANCVGG
CNGSANTVGPNGCNSCHVSIANEAEDGIVKCLNETESCPSGYFASRNPPTALSHLKGSTICVKCHPLCEECDSEGSIYCKKCK
FYKYEDRCVDACPAGFFSNNDTKTCVPCHTSCRAGCSGTTNWDCTHCVNYKVYNDTEHDFALSPNITQRTTAVNNITITTEST
GGVFLESSVASSTTTSAPEYINYTIISNETGEVIVVVEKVTKYEIKPAFYCTLSKESCPAHKPNWTNDGDSDTVCIVAVPVPT
PLTSFVLSDQELGGIIAAAVIFVAVVILTAVLCKQRATAKENTAKLTLKLTGMDGDQEPVTPTDAIPDMSKLRLIKELELRKG
SILGSGAFGTVYRGVWIPEGENVKIPVAIKVLQEGTSPHQNKELLEEARVMASVEHPCCVRIVAVCMTEQMMLITQLMPLGCL
LDYVRNNKSNISSKVLLNWCTQISKGMHYLEGRGIVHRDLAARNVLVQTPNQVKITDFGLAKLLAYNEGEYHAAGGKMPIKWL
ALESIQHRKFTHKTDVWSYGVTLWELFTYGQRPYENVRARDIPDLLEKGERLPQPSICTIDVYMIMIKCWMIDAESRPSFAEL
SEEFSKMARDPGRYLVIEGDTLMRLPSYNIDRRQMVSETTDGPEEIILDADEYLNPRDGYEDYPDEGDNLLEEEPTTPGSVMS
YQSQNSMRYPSQHSCNGYPNHLPFQPLTVDTHIPTHQAPPPGSQRRLIREPKYAHLEARSQSMQRQRSSDGSMTGRYTSDPCH
EEDPLLVGELEFSKTLPTRLNHNQSRYKPSALPFVEEDYLQPTDNPKDPTAMELVILRQGSCENADLEAPTPTPNRDPMIAHF
TFDLQHPHNAPHELTHNVNPNTVGNFENPEYYDTDSNGSDSLNKELELHRPKKSNQGSHQNKSRHQDYYNDLNNGLYSDGGST
RGSTAESTSSI
>Membranipora membranacea EGFR-2
MRLFFVSFIRCLLINLAVIISSSLPLILFAPVAASSWMYDVKEVCQGTNNGITVVEDLPENRIASLRNRYDGCKYVDGNLEIT
GLDHDDFYDKDLSFLNSIQEVTGYVLIANVYVKNVTLASLTLIRGDTLFKPPVQVHINGTANSTQGAQQRQSKGYALFVSLNF
KASSNAIGMRLLGLRSLTEISKGDILFENNNLLCYATTDIYWPDILANRTLQHVRVSNDIISSMKPCEPGCHHTCSVKIPEVN
PAPVSEYCWGPGPYNCQKMNKVSCSSDCKNRCYGSGVGQCCNEHCAGGCDGPMSTDCWGCKDFYNDGGCVKFCPPSKIYDAVN
YAWVPNPNRKNTFGKLCVTHCPRGFLEHNSGCVRTCPEGYEPKDDKTCQPCDGICAKRCRLKWPQDIDYVHANNIDSLQGCTI
IDGHIRILQTTIDGDTYKDIPPMPLEKLQILKSIKQISDYLLVQFVNHTNVTSLDFLSNLEKIQGQDPETNGGSLNVMRNPYL
VSLGLTSLQAINDGLVIIKHNPKLCFVSSFKWRKLKLENTNFIGENANQTTYCEPLGLVCDSECKNGCWGAGNTQCQTCRNAE
YRDHCIPDCRRELLFRQPGGEKQCQDCHPQCKTSCTGPTAADCDSCKKYVDGLNCVDKCPNDPETGVYKYNATNGTCQQCHSN
CIEGCTGPGNFIGDGGCSKCEVSVADDDTTKIIECLQPHEECPPGYYPDTNPREPLSFLKYYKVCTKCHEECAECDSRGNYRC
PKCKHFRNEDSTCVANCSTVTYKDTETNTCRACHYTCRRECTGPTEWDCDNCAHFKVFNDSRPVVLPTTQVYTSKVNISYSAG
FEDDSATVNVTSEENIMITSTESHIQPFYCTAQKSCPADKPVPEQDGMDTVCREMKETTSDANSTPSLVNDIIIGAVCGSVGL
IVLLVCTVCCYLKRKDRIENRKLKWTRILTGDLEPPTSTAAPDMSKLRLVKEGELRRGGILGSGAFGTVYRGVWLPEGENVKI
PVAIKVLQDGNSPNHNKELLEEARVMASVEHPCCVRIVAVCMTEQIMLVTQLMYLGCLLDYVKKFRDSICSKVMLNWCTQIAR
GMQYLESKGVVHRDLAARNVLVQKPNQVKITDFGLAKLLDYGEGEYTSAGGKMPIKWLALESIQHRKFTHKTDVWSYGVTLWE
MFTYGQKPYENNRAMEVPELLEKGERLPQPTICTIDVYMIMIKCWMIDADSRPGFSELTDEFAKMARDPGRYLVIKGDTLMRL
PSSSIDARDMLRNSSVAEDGPEEFMEADDYLHTGDSLDLSTADMAAEGQLDENGSTVIDMGDNLGSAYRLRNGSLNSRPRDNQ
NGLRSSTYPRRLGHVYMNGFVPSNGSNPRIADDSVNPSRESLSGTQDRRRPNHILNGGSHTSMNSIKPLKYTSSTRIEELEQE
NQQGVIEPPDYEDITQGNNPVTKSNSMNNRKHPAVYEDDYLQPRSQANRHLQNSHQYINVDLPDHQAPPTPTSAPPMKDKAAA
VFTYELSDLGAGGDKNNFGNPEYFDTNEEADDGISSDELEDLETNLCDPLLPHSNKDYYNSVAAFGSDSSARDSFVST
>Membranipora membranacea EGFR-3
MSLYRRCAVLCLIIALLINLSVAFDELLEAKVCLGEVANNAKVGNTDVDYYNSLKQMYTNCTYVHNNVIIRSMDSLDSGQYPL
DFLKDIKEVSGHVHVKGPLPKGLNKLPFESLMIIRGDQLARYSDNSEEKYSLFIYNNQEITNLGLKSLREISRGGAYIGYNYK
NCFGNTDTIDWNDIFANTTTQTVNYTKNCKTSYECDSNCTGLCWGPGPDDCQERNKVLCADQCDHRCYGTGANECCKPGCLAG
CNGPLDADCWGCRDFYNDKKCEKSCPAASIYNHTSYEFDVNPKAKVSFGKLCLKECPSGYLRFKEGCLLHKCPDGYTDVGSNC
VSCNGTCPSTCDWLHVKSDNNWITSSFLRNESNHNCTKIIGDVHLVSAAFENWAKPEGDPVTGELAITLDDMKVFSTVEEITG
SLIFEPYSYAPSITTTDVFKNLRIINGAAAADYSLVIESNFLTELGGFVNFEGILSQNIYVAAPIMCYPDAATFVYWARSGIA
QIDVLGTDCEDAKCHEECAGSCVGPDDLDCLPCRPDQCDHKCKNFEFEGKCVPDCPGPELYENSTSGTCLRCHEQCAGGCFGP
RDTDCNECKNYKNDKRCVPQCPTPTYSDEESNCHPCHDLCNMEFGCSGPENTIGWNGCRACDLALLKKDGSVDQCLNSTAKSQ
TCDSGFLSITQMKWINRSPILSKLITSDTLGTLCLKCNEKCGECESYPDTCTQCSDTLLELVKQSVAVFTQKECLYDCNPRKH
YKNETTRQCIECHDTCDGCYGPSDMECTQCIGFKLDMNGTDTFQCVHECPAYANYTWLSQATKERLCKSEPEPKQGTVGTSIS
PSNDTAVIAGAVVSSFVVIVIAIIIITYCCRQRTKNIAKAKEYELQLVGPGTIEPVTPTGADPDLSRLRLVKESELKRTVIIG
SGAFGTVFKGFLIPEQENVKVPVAIKVLIEGNSPSQNTELLDEARVMASVEHPCCIKIIAVCMTDPMMLITPLMPHGCLLNYV
KENPGSLGSKTLINWCAQIAKGMEHLQRCGIVHRDLAARNVLVQSENQVKITDFGLAKLLDVGEDYYRAESGKVPVKWMALES
IQHRIFNSQTDVWSYGVTIWELLTFGQKPYDGVKAKDIASLLERGERLSQPTISTIDVYMIMIKCWMLDENSRPTFTELAQEF
SKMARDPGRYLVIEGDQLKRTPTVKMNVQEWMFDDMNMTGNDDDYSEKHLVAPDDYICSTSTGASYPGYVNENAFPPPFEKPD

CAPGHTTDNLHSPLRYTSSPTLGGQAGFPTDKLTVDTEDYLEPVSVRGSQSTPAVDDDGYLHPQSASGYIDVLSPDEKSTVPA
LKRQSYSSSSDGEEDEASREPLLGAAENSVYLRTGSTAKPSHNPTTSKKPSKHKNDKQLPNGTNPIYMSNNNGSPVAKQAPVS
AHDYNNIDYVNQSRFNPV
>Lineus ruber EGFR
MHLRDLLGIFALLAVFRRGWSIDIEYLGKVCAGTHEGLQSRPAKFRYDFLRERYTNCTYVNGNLEVTFLDDADITYDLGFLEN
IREVTGYVLIAYVYADYVPLKKLEIIRGQTLFSARRGEPKKYSLYVVANVKPNDKNVGLKELRFINLREILRGEIMFHNNNLL
CYENQINWQDILDGDVNFKFDSTYYRRPCTAPCDKACEVDGRRLCWGSGPDYCQTLFKKMCSPQCDGRCFGPNPNQCCHAQCA
AGCTGPRKSECRACQNFFNDGQCEPFCPPLEIYDQSEFKNVPNPNARYTYGSICVKKCPDNMLTDRSACVKSCPPDKLATKDL
KCVPCDGPCPKKCPGMESEYLDELNIHNFTNCTIIEGNLRILKTTFDNDPYKNLTALDPAKLFKLSTVKEITGYLTIQHLPLE
VKNLSFLGNLEKVSGRDVLSTHLSFSILYTSITSLGLKSLRSIMAGGVMIFDNRKLCYAETVKWEKIIKDPKLLQLPNKYLFL
KENRKKEECDQNAQNCSVECDGCWGPGNSQCQACNNYTFNGLCIGSCKSQPRLYDAGNKTCKKCDDECSSGCLGPGPRNCTGG
CKNFFDGPVCVSTCPIQKYPDTKKRCQPCHDNCVGGCTGPENNIGEGACNNCGFLKMNEDGTTIDQCLDKNPSEYFCPDGYYL
QPGQVGKDKVTKKLWKGLPKCFKCHKLCKTCLGYGTYSCPLCHFYRFQHSCFSECPTQDTYPTRKEGFDIECGRCHVQCKRGC
DGASSTQCTYCANVKIVTEYQDEITGEIRTTHNCTQACPEELPYVESDPETGEDLCVGELNSAKMNIIIGASVACILIVIIGI
IIIAYWCRQRMLAKENMLKLTARMSGYDESVPLTPTDAKPDLAKLRLIKESELRKGGIIGSGAFGTVYKGVWVPEGENVKIPV
AIKVLSEGTCPSQNKELLEEARIMTSVDNTYCIRILAVCMTAQMMLVTQLMPLGCLLDYVRKNKANIGSKALLNWCTQIAKGM
TYLEDRAIVHRDLAARNVLVQSPTLVKITDFGLAKLLDYDEEEYHAAGGKMPIKWLALECIQHRVFTHKSDVWSYGVTVWELF
TYGQRPYESVRARDVPDLLEKGERLPQPSICTIDVYMIMIKCWMLDAESRPSFRELADEFEKMARDPGRYLVITGDKLMRLPS
HNVDTQDLVRNMSIAVDGPEEVIDAEEYLMPKSTETTEEIDSPVLVNGINGMGNGKVHIPPEYDERTPANSPARENQFLITPT
KRDKKYAHLEGNFNRDRGSNGGSRYSSDPCKILSTTKDPRDDHNDFLGRDPMKDYALHLPVDEDYYLQPKSSKPAHYMDIIDT
DMYCKPRSMEPNSVDNPEYFQNGHHDNLPASPTSETKLRLDSSSPDSEGPGHEYYNEYNRLQQCDPLVLHPGETTV
>Owenia fusiformis EGFR
MYSHWILAVLVMASVVVEAIADPQDFDQDMDQVCQGTSTGMGFQGSASRRYDMYRQRYTNCTFIDGNVEIVFLDDDDVKYDLS
FLKDIREVTGYVLIVSVYADVPFLTNLRIIRGRKLFGHEGKEYSLYVALNYQKSAISKQVGLKELQFKSLYEIMKGNVFFYNN
PFLCHANDILWDDILEEDVYFEETDDVPRRVCGSCHASCETYCLGNNDDPDKCRKYCWGEGPDMCQKLSRTICSPQCDGRCYG
KEPNKCCHSQCAGGCTGPTSRECWSCKHFKDGNKCVAQCPPEMIYDDAEYSMVKNPNAKYAYGTLCVPKCPNHLMQNNGYCIK
NCPPGKMEGKDKDAGKCVPCIGPCPKKCPGTTADEFVNSNNIQRFKDCTIIEGNLRILESSFTGDVHRNIVGIEPHELSVFES
VREITEYLMVQSRHENLTTLSFLKNLEVIHGRATDQQQSAALSIVGTKNIMELGLTSLKRVVNGNIIIYKNDQLCYVNTFEWS
RIKESSIGTVVVRQNKENAACTAAGEICDPQCTSDGCWGRGNNMCQACSNKQYKDICIEECLPSLGIFHAGDNTCEDCHEECA
PVTRRNTFMCTGPNSNNCKRCKNVKDGPYCKAECPTMKYPNASNHCMKCHENCLEGAGCTGPNNTIGLGACNDCEISQAEDDN
PNKIARCLAPDTENCAHGYFMKPRGERRICQKCHPQCDHCNGDGLGLCIGCKHFEQEGQCVSQCRKDYYPDLVEKACYRCSDE
CFGGCTGPNSTQCHVCKLLKVYIDEENNIFNCTSECPPDLLYDIQEEETDEKVCVNAQNPIIAAKLEQERKNDQQKIGIIVGS
SVGSLVLLIAGVIILAYCCNERAKHKEQTTRITARMTGIHDENEPLTPTDAKPDLSQMRLAKESELRKGAIIGSGAFGTVYKG
VWIPENEGVKIPVAIKVLQESTPNQNKELLDEARVMASVEHTYCVRILAVCMTAQMMLVTQLMPLGCLLDYVRKNKSNIGSKV
LLNWCAQIAKGMSYLEGRGIVHRDLAARNVLVQGPNQVKITDFGLAKLLDYQEEEYHAAGGKMPIKWLALECIQHRIFTHKSD
IWSFGVTVWELFTYGQRPYDNVRARDVPDLLEKGERLPQPSICTIDVYMIMIKCWMLDADSRPSFRELAEEFSKMARDPGRYL
VIEGDKLMRLPSETYNNRDLVRSISDTNGPEEIVDAEEYLQPQNVISEEGTYDRQPILQSPRRPMDYSDGRQQRSPRTPGAFP
YNKGANVNLPKIRDKKYGHLEAAADNRGRGDSINSRYTSEPCQVFSMNDGRLREDDQPPVFSPPPPGTPSYHHHPSPLVDKYP
RSNGQPLRLPVDEDDYLTPQSTSPSNYIDIIDGNVPSKDNKNYVNLQDQQEPSQENYLQMDSPDSPVNMDNPEYFQTGAPELQ
RGNPKSEPQNRTRTSSNQDQEHDYYNDFDKIKQMERQPLTIGNTGSESHV
>Capitella teleta EGFR
MIPVCHGTSDGLTHRGKAHHRYNDYKDRYTNCTYVAGNLEIIFLEGDFDLSFLKDIQEVTGYVLLVSNYMSLIPLTSLRIIRG
RSLFPHKDQYFSLFVALNFEKPDNSTIGNGAVSMPVHGLRELSLTSLYEIMAGSVLFFNNNLLCWENTINWDDILTGEGAQVT
HSIDSPHGRADFCHDNCTVTLPGQEPDKHCWGPGPEKCQKMTRVVCSRQCDGGCFGPLPADCCHPECAGGCLGPLKTQCWACK
NFNNGDECVEMCPPLLIYDKDKFARVKNPDAKYAYGTLCVEKCPDHLLADDGGCVRDCGSGRMQGKGSEEGTCVDCDGPCPKT
CDGTSEMDSGSFVDANNIASFDGCTIIKGGLKILTNTFTGDVHNNISAMHPKRLSVFETVQEITGYLQVEGRHPDFKSLSFFK
NLHTISGRILTTEQVIYILDTGIEFLGLESLKELPNGNIFVHKNNHLCYVQTLSWLHLTASGKQRTTISQNANQSLCEAENKV
CDPECSSNGCWGPGNDQCLDCKSYSYGKSCLSSCNERKDVYEAENQVCMDCHEECLNGCTGPNSDNCNECKRVKDGPFCVASC
PEGSKYSDPAGICRPCHDNCKGGCTGPLNIVGENGCNDCHLRVMKDDEGDTVEKCLPENSECEIGYHRDHQKLPDAFGMGNMQ
RSVCRPCHSLCKTCTRPGIYCDSCTYYKQMNLCVSNCSSDHFPDNSEATCVSCHNQCLKCRGPTEADCLECKSYKLYLDTEHT
DGDSRFNCTDVCPEWAANMVVVESGEEGQTKKETICMNDERAEKILGQPRFKIPMILGIAVGGFVFLAVVIALVFLFRRQMAR
SEENKARLTARISGLEESEPLTPTDAKPDMAMLRLIKESELRRGNIIGSGAFGTVYKGAWIPSGENVKIPVAIKVLQEGTAPN
QNKELLEEARIMTSVEHPCCIRILAVCMTAQMMLITQLMPLGCLLDYVRKNKMNIGSKVLLNWCTQIARGMAYLEERGIVHRD
LAARNVLVQNATQVKITDFGLAKLLDYNEEEFQAGGGKMPIKWLALECIQHRVFTHKSDVWSFGVTIWELFTFGQRPYESVRA
RDIPDLLEKGERLPQPTICTIDVYMIMIKCWMLDAESRPSFKELMDEFAKMARDPGRYLVIQGDKLLRLPSHSYDTRDLIRNM
SGGMEGVEGPEEIMEADEYLQPQVQIDPRMLNGKVRVISIDC
>Helobdella robusta EGFR-5
CPGTTYRHAYEAHHKNYYEFLKQRYTNCNIVDGNLEINFLVKRQDGYQEDEEAGFDLGFLKDIKEVTGYVMIQYNEIFHLNLS
SLLLIRGQQLCNCFVVSVSSFVNNCSLFIPVIIMAHLSFSNCPEISRGDVMIYNNGALRFVDTVNWVDVLSDGSSRVFMSRNN
AICELGCHPSCECHPSCADLTGQPYCWGPGPEMCHRLTKVHCECDNRCEPNSTVRCCSKECAAGCSGSSRKCLACKNFYNDGV
CEENCPVQHIYDSPQYKTLENPKHKYAYGSTCVKPCPNHLLMDKQACVKHCAANSMIVGDSCVECGSDGQKPCLVEGVAVNAS
SFIGCQVVDGNLMFDDFTWNGDVYLNLCHKLFLSHVSEEWKYLESVEEVTGFVRMHFRTSNYESLSFLKNLKTIRGVFLDELG
HSLGIAATNFTSLGLDSLTAIKYGNVKISRNKKMCYVQSINWSAILKSPDDQRVIIGNNNNNCESEGKLCHPECSDDGCWGPG
DHQCISCRNFKFQNKCVLSCQSIPLAYASSNTTCSLCHEQCLDTCDGPGAENCTTCRFVRDRDTCTTSCPKTKYTDKNNHCQN

CHKNCGEYGCTGPLNNIGANACNSCDLVSFEKVGDSLNATSCMPITKMCATGLYRTILIDYPSNHSLHRKQGCKPCHPECLAC
WDYGTSTSYCHPCKHFKEEDKCVSSSIKRKNVEDYDERSCLPCHTSCLTCYGPDPHQCYTCKHYIVYHDYQNKELGFNCSENC
PANLPHKLARDVNGMKITECVEDDYASKTGRILAIVLPICILTVIAIVAISLGVVAYKKSKAKTKIAMAAVNLLNGIEDMPLN
PSGLRPNMSRLNIISESELRLGEKIGQGAFGSVFKGVWTPSKKNVRVYVAIKILHETWSNTDVRQPDYLSEVEIMCNVKNEYC
VQILAVCLASKTMIVTQLMPYGCMLDFIRDNQNNLGSKVLLNWARQIASGMAYLEEVRIVHRDLAARNVLVQNDNHVRITDFG
LSKLLDAGTNIYYSKGGKIPIKWLALESLRHKKFTHSSDVWSFGVTLWEMFTFGDSPYENVKSENMVEVLEKGDRLAQPTICT
IDVYMVMIKCWLVDADSRPTFSSLVETFSHMAEDPTRFLFI
>Helobdella robusta EGFR-2
MHTHKQTYTFTHAYSHTNKQTYTHTHKQTAGECSSECQPFEGKKYCWGPEARQCQNFTRVICSDVCADRCFGPGPDKCCSKAC
AGGCTGLKDTECFACARFDNEGKCVDNCPPPEEYDPVQYIHKPNPFGKYSYGHVCLARCPASMYELMGACVKRCQPGTTPKTV
NGRNKCLPCDGPCPKACQPPSILDSDNIDNLKDCTIIEGPIILHQSMAVGDQWTKPLTADQWKIFETVTTIEDFLYVDIRAAN
FTDLSFLKNLKLINGKKTKKDCSLFIYNSLLESIGLTSLEGIYGGKLCVISNENLCYMTQEPEDWNFLMADGDKVRFDGNKLP
ELCVKDNETCSDKCLSKYACFGASDEDCPACKQLKEGRKCVDGCSPGWFEQQRAEMTSVGQPIRECAKCHAQCKDSCNGTGAN
HCFSCLNYKDGPYCVEKCPERMKGMKKLKWPMKDLSIVECHRCHSECRECYGPGASNCKLETYGKQDSKVRMMTMMMVTIMVM
MMMVTIMVMMMMVVVMVMMMMVVMMTMMVVPLVITGAAPDKSSLKIINESELRKDAEIGSGAFGTVYKGVWVPSNENVRIPVA
IKVLKEGTTSVQNNEFLDEARIMASVNHPCCVRLSAVCLTNQVQLITPLMPFGSLLEYLRKNKSQIGSKVLLNWATQIARGMK
YLEERGIVHRDLAARNVLVKNANSVKITDFGLSKLLDANQESVKAVDEKLPIKWMAIESIRLRIFTHKSDVWSYGVTLWELFT
YGQRPYEYLSATDVLEMLEKGERLSQPEICTIDIYMLMIRCWLLDADSRPSFQEMVEEFSRMSRDPGRFLVITGDEFMKLLEN
NPDARDLVSDKGAEKWMLADEYYDQKSIDALKNKTPEEDNYLKPKKDGFELPPKKAKSNKYVDVNEGSGNKYTVDPTESQGQL
DDGQLDVPIDSEYVSGGPLVSEPAGLEKKPFFKGSQSSLSNLARMAVEGLTRSSEYLKMSTNKKPKEEKKAILAEFEDDAIDD
VIIPPITTTTTTPKTSKTTPKITKQTASSSVEDDDSDDYLRHVVSIKTLHNNTNIINNNGNNNSSNNKNYGHNYYNV
>Helobdella robusta EGFR-3
MKTTQVFYFYVCKGTQNEAITRYESLAQRYTNCTYVDGNLELVFLTNSSLNLSFLSGVREVTGYVRIFGTYLDYIPLDSLRII
RGRSQFVEKGVGYSLHVAYNFDPKDPVNVGLKELRLTSLHEIQLGNVYVQHNNRMCYSDHVRWDDIVTGANSLVIVNETFDPL
YAHRQCSDCDLSCEIMGRRYCWGNTSNSPNECCDAECIGGCVGPLKTQCWACKNFDNNGSCLTFCPPEFIYSHTLYKSVPNPA
FKYSYGTLCVDKCPEHLVSRQGACVQECGPGMKADEERKCVECNGPCPKSCNFEVAYLDSENIKSLEGCTKLIGNIVLLELSF
EGDAWMKIPPMNVSSLRYLKDINEITGYLKVQATLDQFKNLSFLSNLHTIQGRILDGQGASVSIFQTSLQSLGLNSLKSINTG
GIFIANNSQLCFAENINWQRFIKNQSRRAVVRFNRNEQDCQRDLEVCHAECSNDGCWGPGNDQCLSCRHFFYNFTDLHGNKAT
KCVTSCSSQPGLYLISGRECGLCHDQCKSNCSGPGAEKCDSCKLVKDGPFCQASCPPSKYPDEHFLCQTCNEHCGPEGCTGPE
AYEKVGGCKACSLPIYGWSEQQATIPQSTPSTSSPLLPSPSSPPSLVDVPRPSSPASLPSFFASSIKCLPVDSKCPDGYFKRH
IKTACIKCHSLCRTCNGPSSDECIVCAFFLEAGRCVRSCQSNYYVSIEYDHQSQSVLPFSYTGLDLSKSTSIKSVVGTLTCKR
CDESCLRCVGPSNRNCTACKHYKVLIDEDDGAAAEEYDGESDVSDGGSRINTNGYDVKFYCTERCPEDKPDAHVNNTETICLP
IDYVAKRRRDVSVALGVLISFIITCTILGVIFIRRKEIIKKFNKYQAEMRFLNAKSDVKPAKAEPDLANLRLINNSELRTGAV
IGYGAYGTVYKGYWFPTDGNPEGMPVAIKILKEGSQELLAEARIMASVRHVSCINILCLCLTENLMLVTPLMPQGCMLDYIRK
NTKSISADVILRWSTQIAEVEFANLKYVTVESLDQVKITDFGLAKLIDRDDIKIGGESEGAMKLPVKWLAYECLVNKIFDHKS
DVWSYGVTIWELCTFGDRPYKHVSNVKLIPEMLREGIRLEKPQICSNHFYYILYSCWNLNPNDRPTFSELVKDFITMQQSSGR
YLTIPPDGRLPLERHHKIPASHQQSLEFKQKPFRFGYESKSSGSDVRQSLLSDKPVDYLMPGNSPEPPILYNNLMYLKQQNAV
SNGSAKKSPLYVNSVKPSSSSDILEPTYVPTLQPSSEYYNDFSPGFLNRPIRLRNKGDSSYQHYKAPNTSNTTGQQQTEDCGS
IPTNIRENSMTDDYISVLSAKSLYRNSTLSNGALAQQNQQSSEFSLMPESKQRLSSSSASFNPKRTSLSSSHSIPSPGLPFSV
APPTEQLTNQMRDSFYGSENARNSQEDHGYYNTLSF
>Helobdella robusta EGFR-4
MTKEPPETQLQRLHNYFTNCTHIQGNLELTNIRNHNDDLSFLKYVEEISGYLLIFNVDIKEILLPKLKIIRGQTLLSYKHHKD
NSLVVLLTNPNKVPNLVFQNLREISYGNILVHTAYKCLFEENLSFKDFFVDSNSKIITLGPNVPQKCSTCPMVCKLNYDNKET
SHCWKKLNKPLCQKLSKVICSPHCGAGRCYGRGENECCHQQCAAGCWGPKDTECLACKHYKDDERCVESCLPHVASTFKHSAS
HTSSDVRIKFSHHAFCVDTCPDNLLIEGRACVEECSPGFKTSLSSSPTSVLFPSLLSNSSSLSDRLCLPYSDAASNKDVCKGT
DEVLSSENINNFINCSIVDGNIHITDISFLGDRANGIEPLPVESLMLMEHVQHIRGSLVINSLKRHPQVDSFYFLRNLETIGG
SKLYHDKWSLFVQNTNFKFLGLSQLRSVRRGRVYILDNEHLCLASTINWPLMRSNGNKSHNSDDIIGNNMNDTNCKLMKMLCS
EECSEVHGCMGPGTSQCIHCRNMNYNGECLPDCTIHSNAEVHLYQINKTKLCGACHQECLGPCSGPDGDDCLEGCKHTQLGST
CVSHCPVGYYVEDDDDVVEDVFKADDEADNNYGDENDTDDIENTGNTANSNETNLKLNKIANNVNSIASSNKQTAGKHGTCKQ
CSSVCYNGCTGNNTHLGRFGCNMCFLGLLSNDDDDDDGRATDDAYDLKEGVNYSGEHGQEAVLGRKRTLNGSRDNLRNETLHN
ESSYDDTNNKKNKNKISRTVFPGGDAHKNISRIITKVVCINASSLEHICPEGFYLDHHFKHPHYEPVAKLGVCRRCHAECETC
RGPGSSMQYGCSKCRNFKEKDVCVRSCSLGWYADGVDGSDECFRCHPLCRTCNGPSADNCTSCMHFAIPHISLQEYPAFWNGA
TAVHDTYDGDNNSSMKQLNCTDECPTDLPFTMLLHSNLFCVTSAAIKSQLEDRKPNVISLALPLLFALVMFVLLVALLIQRKK
RTNRKDEIDSEFIEYINNNDESTRESDVKPDMSRLRLIRENELKRKNVIGSGAFGTVYKGYWTPEGTRNMKIPVAIKVLTEHY
LPSGADDESNSENSILGEAHIMASVMHPCCVQILAICVTSPIMLISQLMPLGCLLEYVKKFEQKIGSKHLVTWAMQIAHGMSY
LEQKNIVHRDLAARNVLVQDSFKVKITDFGLAKLVDRREGQFQSRGGKLPVKWMSIESIKYRIFSHKSDVWSYGVTLWELFTF
GCRPYEDVSSQRLLEVLEMGQRLNQPSVCTIDVYMIMIKCWLVDACSRPTFLQLHEEFNKMLEDPARYIVIPKEESALSKKSA
KENRISYVTTKYIRTKHDRQQTSPGEYIVEQQFGANQTPSPPPSTAGTTTTTYATTYTKSSAPNLPDTLVVNGGITKTLLKNK
PGGNPHSTKHQLVTTFVNRSYNNNVGGDNKQSKKKIHNNRKKIASTDTCDSHKFQYDHPTTSLLRGKNGDSNCKIKDGLASVV
VHSRSTPTNLSMKKVINNIFQNSKTSADLRTSRNICYLEPKSSDAVTYLEIQNSIDDPQANCAEDVTDCSHRVNNNRTPSNKD
VTEANNTAASACDEIEITSLISSPHIPPSQASFLNKEYFQSTEI
>Helobdella robusta EGFR-6
HHHHHHHYHHHHHHQMYRTDNAKPDLSTMRLIKESELMRGTCIGSGAFGAVYRGAWIPEGENLKIPVAIKILQDTTSTSQNKE
FLQEARVMASVDHPCCVRILAICMASQMMLVTQLLPLGCLLDYVKKNRCNIGSKVFLNWCYQMAKGMQYLESKNIVHRDLAAR

```
NVLVQSPTHVKITDFGLSKLLDYDEEAFQVNDGRMPIKWLALECIQHRIFNHKSDVWSFGVTLWEMFTYGRRPYENIRARDIS
TLLEKGERLPQPMICTIDSYMIMIKCWILDAQSRPSFKELAEEFSKMARDPARFLVIPVRKQYF
>Helobdella robusta EGFR-1
PDLSVLKLIEESELKRGPEIGSGAFGTVYKGIWSPVGKRIKVAVAIKVLNEGSSTSLQNELLDEARVMSSVTHPCCIKILAVC
MARTMMLITPLVNNGSLLEYIRKNKADSSTLLKWATQIARGMNYLEQKGIVHRDLAARNILVHNKSQVKITDFGLAKLLNCEE
YYLATGGKMPIKWIALESIEHRVFTHKSDVWSYGVTLWELFTSGDRPYDNIKAIDMAQYLENGNRLSQPPICTIDVYMLMVKC
WLVHAESRPSFAELETEFSIMSRDPGRYLVIEVRKN
>Lottia gigantea EGFR-1
MTVLSFLTAASGRMLPCLASYLLWNEKDKHNDIPPLHPANLTVFEYVREITGNLIIQSSHEDFKNLSCFKSLEYIYGRETNSE
LSLSIIMTKLESLELMNLKQIRRGSVMIAGNTQLCYINSINWSKLLSTNQQKTTISHNKNTSLCMAENRVCDPECSSDGCWGP
GSEKCLSCKRYRIEEKNVCIKSCAHIELHYEGDNMICKPCHEECRNNCTGPEPSECDSCINVSIIDSTNHTICLAECPEIMYP
DKNSVCQKCHKNCANGCTGPMDHVGENGCNSCEVGYRQMKGSSTIKCMPTDTDNCPDGHHMKPVKFDIIDPLAGKRICEPCHR
FCKTCTGAGVYYCNLCRYYFQQSTCVEHCHTMSYGNNDTKMCDTCHTECRSGCRGPDSSDCLACKNFKIYTNEETTKVNSTSF
LIFSVHNTQNTQQVKITDFGLAKLLDYDEEVYQAAGGKMPIKWLALECIQHRIFTHKSDVWSYGVTVWELMTYGLKPYDSIRA
RDVPDLLEKGERLPQPHCCTIDVYMIMIKCWMLDAESRPSFKELQEEFAKMARDPGRYLVIPGRDNLFLEVLAHVRFYSRFEY
SWWETFAAN
>Lottia gigantea EGFR-2
CQGEINELTARGDSKFRYKNLRDRYTNCTYIQGSLEIVFLEKVDSVTKYDLSFLRSIKEVTGYVLILSVFADYVPLENLRIIR
GRTLYHDKYSLYVALNSHPTAEPGLRELQILRGKVFFKNNNKLCYENTIIWSDINPREEPPVEFHFDSIHEKRQCGECHDSCY
QESKQTKACWGEGPDMCQKLSQGEVCHGSCDGRCYGKLPNQCCHPQCAAGCSGPKKTDCLSCRNFNDLGACVEFCPLQEIYDS
QKFIKVPNPNARFTYGALCVKECPPGMLNEDGLCVIQCSEGKMEDKNGVCQQCKGPCPRACLGTGPDKFLTAENLERFTDCTR
IQGTLKILTPTFSGFQLILMKFDKHNDIPPLHPANLTVFEYVREITGNLIIQSSHEDFKNLSCFKSLEYIYGRETNSELSLSI
IMTKLESLELMNLKQIRRGSVMIAGNTQLCYINSINWSKLLSTNQQKTTISHNKNTSLCMAENRVCDPECSLDGCWGPGSEKC
LSCKRYRIEEKNVCIKSCAHIELHYEGDNMICKPCHEECHNNCTGPEPSECDSCINVSIIDSTNHTICLAECPEIMYPDKNSV
CQKCHKNCANGCTGPMDHVGENGCNSCEVGYRQMKGSSTIKCMPTDTDNCPDGHHMKPVKFDIIDPLAGKRICEPCHRFCKTC
TGAGVYYCNLCRYYFQQSTCVEHCHTMSYGNNDTKMCDTCHTECRSGCRGPDSSDCLACKNFKIYTNEETTKFNCTLKCPDDV
PFLKVKKVVHYICKYTVIMIICYNSREEKKKMAIIVGPTIAGVVVLGLLILCIAWCCQRQAKAKEKTAKLTARMTGCDEEVPL
TPTLAKPDLAQLKLIKENDLRRGGIIGSGAFGTVYKGFWIPEGENIKIPVAIKVLQEGTSPNQNKELLEEARVMTSVEHPCCV
RILAVCMTAQMMLITQLMPLGCLLDYVRKNKDHIGSKVLLNWCTQIARGMVYLEERGIVHRDLAARNVLLHNTQNTQQVKITD
FGLAKLLDYDEEVYQAAGGKMPIKWLALECIQHRIFTHKSDVWSYGVTVWELMTYGLKPYDSIRARDVPDLLEKGERLPQPHC
CTIDVYMIMIKLIHLNVQNYPNLVLKFLKGTGAPFFFDQSSAKPISNSGDKLMRLPSESYDKHDLMRSLSVVNDGGPEEVVEA
DDYLQPHPNSDDSIDNNNGQAKVDNSHQTFFLDSVNSFELGVMNYDRNSRKVPLNLPVDEDDYLQPKSHNPAAYMDLSDKGNI
DLKGWGFGTRVSGVWD
>Crassostrea gigas EGFR-2
MCLDKTDTKTSIILGSVIGGVVLLAAALVLFGYCWCQRAKAQEKTAILTAKMTGYEDEQPLTPTNAKPDMSSIRLIKQSELQR
GGIIGSGAFGTVYKGFWIPQDENVKIPVAIKVLSDSTSLCQNKELLQEARVMASVKNPCCIRILGMAYLEERGIVHRDLAARN
VLVQSPGQIKITDFGLAKLLDINEDEYHAAGGKMPIKWLVLECIQQRFFTHKSDVWSFGVTVWELFTYGQRPYENLLEKGERL
PQPSICTIDVYMIMIKCWMLDADSRPSFFELTEEFAKMARDPGRYLVISGDVLKKIPDESNTPSAIELITPNTSNNPKVEGDK
LMRLPSHSYDKNDLARSLSVAVDGPEEVIEAEDYLQPTPRASVEIPTTPVSSKTPLMPYEASSPSSNAIPMKEIQAPARREKR
YGHLESAAKARDQRAQDPSRIRGDSVNSRYSSDPVKVLHTDETDTGHKRPHRNGSAGKANIPNYYTKHSPGFNKMQLPLDEDD
YLQPKSSKPRAYADLIDGQDYLNDSSGSVFLDDHLDHNGYPGLNFQNPEYFDDPNLNVPKKQLTNKSYYNDISAVNGGSDEQE
PLVLSDEMKPETTV
>Crassostrea gigas EGFR-1
MAYLEERGIVHRDLAAKNLLVQSPGQIKITDFGLAKLLDINEDEYHAAGGKMPIKWLAVECIQQRIFTHKSEVWSFGVTVWEL
FTYGQRPYENVRVRGVPDLLEKGERLPQPSICTIDVYTIMIKCWMLDADSRPSFFELTEEFAKMARDPGRYLVISGDKLMRLP
SHSYNKNDLARSLSVAVDGPEEVIEAEDYLQPTPRASVEIPTTPVSSKGHFVHASPPKLIKVEA
>Macrostomum lignano EGFR-4
MSSHSTRKFVQLCLVAFLFLLPDFERKMPVDAVIPDQPTACVGTRRSEFTMSNSVKLVEVLHNSLADCQFIIGDVHIIKINEQ
EFIDQGKPFNLSFLDSIREVTGCIQIIDSCFNTPISFKSLEVIRGKCDGEAITMHFMSTCKMQIPPVDFRNLRYIGKGGVLVG
GLKNCYLEKRINYDELFSDAANQKFESKLDCLEEKESACHESCPVWPNSKKYCWGPGSDQCQARSKCRSIQDCVSNRCYISNG
VENCCSENCLGGCYGTSSRQCFGCTRFNKNGSCVGACSTSKVYDTVSARFVEPREKVFPLGPICVSQCPRSFFVEEDSCVSKC
TTGYSASSNRTCIKMQAIRQCEAVTSSILCGNSNPLLTPLFNSSVMPCTEFTGDISILDSCLKGIPFPGQKILSLQDMEYIFS
RLTVVRGSIYVTIKEPLLLTNLSFMSKLKRLDSAPKIRDSGNLLVISPHIQYLGLSSLQSVAGSIHILSSAVRNQKELKSTSV
QFCPNLFPKQNATTDGIIFSSGSLSSKVTDNFINKIRNGFFRQTCPIDLNRCHVECDPRYGCWGPLSSDCVKCSHVKAGTQCL
SNCSAASGWWFDKFLITSVSPTTSIVQQQCSRCSLLCSRCYGPNENNCTDCVSSAFRDGTKCVKSCSFSSQYADSSRICQNCS
KLCLNPRKNVPTCSGNGSFVGRGGCQFCARVLPELDLTGKRILKCLDVRQTCPDGFYVTVLDQSHLSEFPDLSEVLSSTTLSV
SICLPCADQCKKCQGAAHICTECWRFNIESNEKTPSHAGLKCSSNCPNGYFAVAEEKVCKPCAKHCSECSGPSSSDCSKCDKH
AFTYFPLGFNKSNEFVCLRSCPDSQPHAFLATDLGMTCSSNPLTAISDRENARNEKQATVISGVLICATLLLILVILILFYWC
RQRSQHSKKKPRIIGVWDEDMEPLNVTDAAPNVAVLRTISEKEISRGQEVGSGAFGTVFRGEWFPDGDNQRVSVAIKILSDSI
GDNNVSSELLEESRIMASVEHPHCVRLVGICMTQPLQLITQFMPLGNLRDFLRARKDTDQIGSSHLLRYSSQIASGMSYLEQR
NIIHRDLACRNVLVASNKCVKITDFGLAKLLDNPNEEYRASGGKLPVKWLALECLVSRKFSHKSDVWAYGVTLWEIFMFGDTP
YVNVKPTDLADYLEKGERLPQPPICTLDVYILMIKCWMLNESQRPTFDDLESSFKKMYHDPGRYVYIRGDKYARNPSNESGST
EHRSESSKSPSINHLHSTELNPTGEVEDCNYSALDTADMDNDVFRGEPSLPQTSMPSTSQPHPTIGSDSGYATAPPVSTAAAA
AGASGIASRQPRAQPKDPQFYVNVAKSRLSSHSDNKYTSDPCKASAEGQPLSQLNDQPSAATSDDDYLRPIRANSSSSQDEAL
```

RSPETQSNTVPHNTSTVQNPEYFFNASRRQYHNIATELPPANQGEEADDAFPQLPPPPPPPLPSPPAPQPPPPLVHPLQLQAS
QSTSVGSHSEEDPNLSYV
>Macrostomum lignano EGFR-1
MSICQLLTLLAVALASVTASTAARQLTAYSKLCKTLELTHMDYASHLNASRLRDRLEEMVGGCQYLHGDLIISWWGVDRTTGR
QLGGQLRDMRTDFLDSLIEISGRLIVAQSNLARLELRNLRAIRGHGTSDGDRPAIELSQCAHPIQLVLPRLAAIVQGGVQLYS
ATDRGLYCRITRSVVWSALFQQPGKQRVDYAGVSSCTRDSRPAACDCAGGSASAVACHPDCLSGCWSADTGACQSDGRCKKRA
CAFCYTRGLALEEECCHSACLGGCVGPNAKDCFSCRGVSYNGECRESCPKEYYDTETSKVIELPEPMVQVGNVCKRRCPAPFV
LDSRRRYCLAECPNGELPVEGNCTACADTADGATSGCRVCESAERQLDDNLASSFANCSVWKLSSSFNLDGAAMSDRAWLGVA
SARIIFSIRVIVKRTEERAANLSVLRRLGYVSGELIINQLNASYLALSGLRSTQRLSIFNIRGLCRAWFPMEHLNSTSVGFPV
SYGNLEFADCPDAACHPLCRGGCWGPGPGLCVACANASVDGVCYADCRQAGRYRSPAEEDAGRCLPCHAECGESGGCSGPSAH
QCATCRAYRQDGACVASCAGNLEPDTYGVCYPASTMRLGVLAAGLALLLLLAAVAGLVAWRYYQRRLHRYDMVDLDEYLGDAN
SPNDMAQLLIVNDDDLIKQKEIGSGTFGTVYKGILRSETDCGYKELPVAIKVLKGSNPKLGQELLNEASVLARVRHPCCVRLV
ALCLTQDVQLVTNLMPRGCLLDFLRARQNSIGARRMLTWALQVAEAMAYLESINIVHRDLAARNVLLKTPDEVKITDFGLAKL
LQNSERQLIYTSGAMPVKWLGIECFESGVFSHKSDVWSYGVLLWEICSYGEAPYKPYRISSVDDISNLLRKGVRLSQPPVCSV
DFYNIMLTCWLPNPESRPNFTDLIASMKDCLTAPSAFIAFGPGMDGDADKI
>Macrostomum lignano EGFR-2
PGPDECIACRNYREDGVCVAACGSQLEPDADGECYAAAAARFAGLAAGLALLVLALLALGLLGGWHYRRQVRKYEMVELDEYL
ADPSNPSDMVRLLIVNNDDVVKQKEIGSGAFGTVYRGLLRTDTVKGVRELPVAVKVLRGNNPKLGQELLSEASVLARVQHPHC
VRLVALCMTAEVQLITALMPRGCLLSFLRDHRGGIGAERMLNWALQVAEGMAYLESISIVHRDLAARNVLLKTPDEVKITDFG
LARLLQESEREFVYTSGALPVKWLGLECFESGVFSHQSDVWSYGVLLWEICSYGESPYRPYRIASVEDITGLLKKGIRLNQPD
ICSVDFYNIMLACWLPFPDSRPKFYDLTASMKDCLLCPSKYIAARNAAATEVDMEKAELVHFMAQHQPPPAEHRRVAEVADDS
GYAPMADAAPERQRAGANSYVNEPQLRSRPATNGCPTSAEAAGTGDYLVPEARPATAAYDEPDAGYLVPQQPQPVDIDDDYLA
PTKS
>Macrostomum lignano EGFR-3
SFDNTSETDKERSFDNTSEVCRNAVCDPQCRDECWGPGPDMCVACRNFEVDGRCYATCQEAGRYNLSGECQECHSECEGGCSG
PSASECERCSNLEEDGKCVAACSSREMRADSKGRCYSVASARLLTVGIGVGLLILVLLVLLPVAYVHYRRRMRKYEIVDLDEY
LTDPSNPNAMAKLLIVNDDDVMKQKEIGSGAFGTVYKGILRSSTNKGVRELPVAIKVLRGHSPKLGQELLNEASMLARVQHPC
CIRLVALCLTQEPQLITALMPRGCLLEFLRAHRNQIGAERMLRWGLQVAEGMEYLESLGIVHRDLAARNVLMENLSPGPPRLK
IGDFGLSGMFQKRADAEYLYYTLPKDNRKFPMYILPPTDLQTKIYPTTDSWSFGTLIWEMFSHIPVLEAFYVNPSRDNGPLHL
VEYVKKKPQKYLLPLDSVPPRLQGLVQRCWEPDHIARISLQDIYSFLHNDVQAIFTEPNGIEKSSTRQID
>Macrostomum lignano EGFR-5
WTPAPAADSPAPPGQPLLVAAKTLRNPGANPQAAEDFRRETRTLARLRDPNIVRVLGVVSGSDLCAVLVEYMQHGDLHQFLRS
LGTGGGPEPPLSYGCLMHMAAQVASGMAYLERCQIIHRDLACRNVLVASAKNVKITDFGLAKLLDEPNEAYTASGGKLPVKWL
ALESLVSRRFSHKSDVWSYGVTLWEIFTFGEPPYANVKPKDIADYLEKGERLPQPPICTLDVYMVMIKCWMLNDQHRPSFDSM
EIEFKKMATDPARYVYIQGDRFARNASRSSNSEAGSSAERQSSGCEEGNESPPRHQPVRSPSSAAAAAAAAFRQPELSYTTLD
HHEQGTEMAAAGGGAAATSPSIDYLDTSLGGGGSSSDHVFAEAETAFVDGPSTSASATATTLPARQSPLPPPPPPHSRKASSS
GRQPQKFVFNDNSYTSDPCHQGADSPEGYLQPISLQRGGSSASRGPLPALPPSPPPSP
>Macrostomum lignano EGFR-6
VDHPHCVRLVGICMTQPLQLVTQFMPLGNLRDFLRARREPGQIGAAQMLRYAAQIASGMAHLERCNIIHRDLACRNVLVSSSR
CVRITDFGLAKALDTASQEYQASGGKLPVKWLALESLISRRFTHKSDVWSYGITLWEIFTFGEPPYPNVKPQDLAEYLEKGER
LPQPAVCMLDVYMLMIKCWMLDEHHRPTFDELEANFTKMCSQPERYVVYNRGVRHPSHCSSSRVSGNSRGGSGGGGPKSPRHSN
NNRQSLDELDDAYVAMEDEVAESDLDNDVFVVDQSTLNTSTTSTACRRLPLSPQPLLYPSVHQNQHRQLLPSAPPPPEQQLSQ
PPPPPPPSADPLTGIERISLAEYHETLTKLAAGHWNRCQSAGQRGDLHAALYSCAQAEVAFDQASRVPSVGTEARESAARSAA
RCRERRAQLQRALSANSTTQAAAAAVAAAAAEAATTFNNFGYVGGGDGGGGSLPDDSGCDTLSDDRSPQPQPPPQPPGPPSSS
LYISNIKKKPPPPPPPQRVSSMQRTLSQSDLFESAAAADAAPPPRAARTPGVVRKSCLQQQQQYPTKKSVTFCELVTEVQVTP
TGCLSEAEDEDFDEQVQQQRQGGEQGISGGIVWCHMCRRQPVNNTTNSNTILANSSNSLQCCEACQAYIAKFAPGQT
>Macrostomum lignano EGFR-7
MSSLYRSVCLVLLSSWVVLNEAAYNPPVCSGTKEADLTGTGSLSNMVLLRKRLSSCKFVIGHVHILDIDENELKAANKSFDFS
FLDDIEEITGCLWIIGSCYSTPLSFKSLRIIRGRDCSYSTHLGNSSLVVLHTDKSCRLKQPPLDLRSLRYIGQGNIYLENVTD
CYLQKFINSEELFRDTRIQTVKPSTNCTGLADRCHPDCPVWPSDGRRHCWGPGPDQCQARSRCISVSQRECPRQRCFMESAKT
ERCCSEQCLGGCFGVQSNQCFGCRKFNSNGTCVDTCSLKTVYDPAKTQHIQSATKLYSYGPVCTARCPDSFFIQVDACVSKCT
DGLSANADNQCVPTVASHICRAPVDSGSFICGVDNPLMEGLTNESRLCTEYIGQFVINSVCFSEKSLFGARRLTLAQLYKMFS
RLRVLRGSAQINLRDFPEVTNLTFLSKLERLDVTPGRSMEHIFVNSEHIQFLGLSSLRHVISTEIIIALMPRPTASSNITFSY
CPGFLPSDRAFADGLIQFRNASKNGLVRIDNAIAEKLKNGQLRSGCPYSQSAACHSQCDRSYGCWGPGPDACVRCTGVRGGSV
CMSACDAVAASPPTAPGWFDAGRSVQLGVGNVSERVCSPCHRSCAGCTGPTSTDCSRCVPGLYLDGSQCLPSCNRDYQYPDSN
RVCRKCPQVCAKVGLGLPTCTGNATIVGQGGCQFCTLIMPIKLSPTDSSLVLQCHRLADSCPIGHYLSALDPNREQSDGLLDD
FLSLQKRASLSVSICRKCDSRCLRCSESASDCSECRYYTIAPSASEEPPARVGVRCSGRDEKGTCPIGYYPVHSNRTCLPCHQ
LCSNCTGPSSQSQDCHKCGRAAYVQLLNYSRPESSAFTCVERCPETHSYQVFYDPLRAWTCSSSPLEVRDIEHEDSSLFQASS
TVAVAVVLALVVLIIAVIAVFVCYWMQQRDGAKRGKRTAFDQSADDDLEPLKPPDGSTLPNRGSLITISEKELVRGREVGSGA
FGTVYKGEWISEAEHRKISVAIKILTDSGDTGMSSELLEESKIMASVDHP
>Macrostomum lignano EGFR-8
MLMHLMALCLLLPLSAAIDTAAVNASSEYVVCTGTLEAEQTNEDSVRKFKALRDRLSGCQFVIGDIHLTRIKQEHFDEQDEVL
DFGFLNTIKEVTGCVKLVDSCFSSPLEFESLQVIRGTGCGNSSLLLQFDSNCALQTPYVAFPNLRHIGGGIILYNVSSCYIER
HVSFSELFAYPMAPVVSRIGQCRDLDNACHSNCTEWPEDGQQHCWGPELRHCQPRTRCRHLSTRDCPYQRCFVDSQGGERCCH

EQCLGGCFGPLAQHCLGCRNFDRNGTCVQKCELENYYHPGAAQNLSPVEGKMLPHGPVCVTKCPGSYFTEDGSCVKKCMSERK
PDADGVCVAVDESRVCEIEVGALSTICHSGNPLMQGLTNASMLCTEFHGSVTVNPDCFSDEPKPGKVTVEQLYTMFSRLRVVR
GEIQLLLFGYNQLRNLTFLSHLERVDDRPGEKKSGRIMIVAPEIHFFGLASLRFINKYIMFLALPNGSSVSLCTGFIPKQDAV
NESLIQLGASTTSNWLRSPEGLDNIRVRSICPIKPDSCDPQCEPRFGCWGPGPTNCVRCRSVKAGRICLQSCESSPGWFLVNS
TKLLEKQGNTTLELMCSRCHHSCAKCDGPGPSNCTACLNGTFLDGSVCVAKCNPETQFEDVSARACQPCPKICTSLGNGKPTC
TGNKTLVGPGGCQFCQHVVVTQQSEADFTPLLKCGPSSCPAGFYTALVDFGSNQFNNFSDFLAVKRTVDLPVTMCAKCAPHCM
TCKGASNKCTACRHYSIEPVLTDEFECETRAEPACRDGFYADHGNQTCRPCAPPCSTCSGPGEGDCLRCVAPKYLVLAESEDG
PVDVENSTSFCTDTCPEDRPRLIASKRGLICTAALSTSMIGVLIGAAGFAVSLAALLSFCCIWRRRRSVMLPEEEKEELEPML
NRGYLATISENELARGPEVGSGAFGTVFKGEWTPADGDGRKTTVAIKILADSGDNGLSSELLEESRIMASVDHPHCVRLVGIC
MTQPLQLITQFMPMGNLRDYLRARKDTSQIGSSHLLRYAAQIASGMAYLESAQLVHRDLAARNCLVSDGSVVKVADFGLTRCL
EGDSDVYVARRGAKFPIKWTAPESLAYNRFNSQSDVWSFGVLLWEIATYGCSPYPNIELTQVYQLLESGYRMDKPPGCPDSVY
QLMRLCWLWEPERRPAFGQLHDELTGLCNQGCLQSGGGGAGREDAVASAGSNSNPADDVASHHSGSTCSSNRSRRFDLQQQQQ
QH
>Schistosoma mansoni EGFR-1
MFMLIYLMGWFFKLLVASTANTESVKVCFTHMNSIGGYPKEGSYEMIKLILDQNCTHIMGNLILTGLYRKADGSDPDLSFLKS
IQEISGYLIIMHSNVNNIPLSNLRVIRANNGGYKIHDELDFAALIIRKNYKDGETLKHVDLHNLKSIVRGSVYIYDNPGLQYL
PDSIHWTELFESVGEQNFYSHKLIQTNSYGEKNITIDLQLYEHNPHDIEPEPVSASVKSSCSKLCPKINNNTYCWGPSPSDCQ
YQSKCQNLLCNRCLRIRNSYSCCHEQCLGGCTGPLASQCVSCKEYYNSGTCVAHCPSRTIIHEGKLISNPEFKYRLGNLCLSV
CPDGFMVEGDVCVTHCSTGSMSTDGRICLPCQDKCPKSCKITDKETKSGRKMSNFDLGNLNQLINCTILEGNLILTKESFNPS
SDLTDHIPITDHHQLWALHSLREVTGYIYLDLELLGDSLRNFSFLENLVKVSGYLPSARVSIMIINSKAHFPGLRSLRQVPHG
RVIFHQNPNLCYLSSLPWTNSAHTSIFSSLNQTNSMQITVIDGPSDKYCESFNYVCHPACKKEYGCWGPGPDQCVRCSYRRAG
LHTCVQSCSNLTGYMSEQWEKQLQISKKNDYFGNSLTKNFHLNYHTAETEDVCVPCHPECGKSCTGPGAHECIGSCKTAWSDG
QCVSECHANTYLNMDRRICEPCQTHCHQRQLTKQPVCTGPGRHPGKGGCNKCEKFIVQSPFNQMNTEISDLASSLTTISLLCV
RGDCPPGTYLTTEVVQPNTLFAKYAEIFTSVAAVCRSCHPRCPVCTAYSLLRANEQRLGCMKCNGFWLRDACVEHCPVEQTYA
IWIKTNRNVPGNSSENHLNLKDSNLKYNGIYVRHLNGQCLACHEQCHAGCWGPGPDQCNHCLNVKVPIRFISDNLTNVYGLDQ
FSEDDLSIGMNSLNLHLYHGKFICLPQCPNDLYNHIVNIPEMEGETICHHSTYLSASDYELIMQDNVYPFQYFNNYPLSNNHQ
QLEQGTSAGIIGLRKSSSYFSSVNGSIMHRKLKNDPVILIMIPLCIILIVFAVLVFICYRYRSNQQYIKKYEHSPLLLLKSLF
CPLSFISKRFVSDISNYKYKKGNPIINLSPTNYHYKNRNDESGTEINLMQYPGTEDSLLRRSSVTGSHRYPNQIVNGQKAPNL
GRLVMINLDDLCLNEKSGPLGTGAFGAVYQGIWKVCQTDYPNLPNINYVITANNEEELMLKVNSQSSCSLLTTSTPMNEMNVV
STNQINSSNIRENEVNTSATEQSSIINQKNNNTDEDISQTSNSVATTSKASTEKSKKEYKLLCVAVKILNEVRGSSDLQALLD
EAKVMASVSHYHCLPLLGICLSQSRKCLVSAYVVNGSLDRYLRLRAPNIDSLILLDWASQIADGMAYLQSRGIIHRDLATRNV
LVTSPEHLQITDFGLAKMLESEEEAKRNEVIVCSGRVPIRWLACETLTHRIYSFKTDVWAYGVTIWEIFTFGDKPFDRIETAN
VKDHVLAGGRLPQPDICTLELYQVMLNCWNENPDARPNFVELYNMFKDFARQPHLYLHPRNESSSNTEVYSSTGDTRSRTTIY
PTTRLSGERFRQPDQTVPSFQSMKSRNSCSPLQNSSSSSGLTNRRFDVGRRTRTSLLLPPTVLQANRTGKYPRHKRRSVGART
DYTMSASLSINSGIGERWGNSSFATHSHPTLIKVDEANGTANISHNFKINSNTSGNNRNNEQFNVGLFSTDSWASSGQPLLSS
DQESSLSHLMLSNFGYTSSESRSSMTVNSPGQHNRNFSLAGSSVPTESRNSEQQSVDEPTSNTQRIGLDVRSGLPVNTSFEAA
AGYVWPQWPDAIPNPENSNLDQANSSETSYVLTAIDSESENNLPFLNRQLWLRRNARQRRYYLRQLQMHRSVADSSLPEQAGE
DAMDETSSWAEPLRSNQSNPNGQQEDSDTDPGSADRYWPDPTYSTQNV
>Schistosoma mansoni EGFR-2
MCQKMLKCANNPDNYCLGGRATTQPCLEECLGGCETRPGNCRACKHAMNDGKCVSQCPPPLIVSREESRTVANPEFKYNFHDI
CVKNCPAPFLKSDSYCVIECDLNTQIPVNGTCKDCPKSGCPEHCKEETIFVNGSLNILQSSSLRKFKSCVYYTGGLYISKESF
QKSSLFPDPIQNVNELYNLLHLKSIVGYIYFDLREAPEELKNLTFLENLESVVLEVKSQSPGAVITIMNGENIESFGFKSLTN
IGGYVYLKNMPKLCYISALTKMLPVRMIDVQDEELCAKRGHVCHSECLPELGCWGADANMCAHCCGLKAGEYCVSRCTDHPGF
YELPTPFNHSILGKTTNNPVCRTLPLTKSDMAEMDEQAIIASVIPSKTCAICHPECAQTCYGPNANQCVGECKHYQHGDTCLP
ECPRNTYIDPQTRHCLPCNESCSHILTTGQNQLCSGPGNFLGLGGCETCWTVIQDKITNKYQCLPDDCPPKHYTESYQTQDFI
NKEKIIVSSHSQVKKGELGGMIRVCKPCHPFCDLCTANGTHASICHSCTHWWFKSECVEICPPAETYSLAGSDKELDNQEMFE
NDLITLSNNTQFSSNQLKEFTFLSNVSTTTNASIDDNQQEYKTSSLAAPVFLIKLKRTQRRCLLCHEQCIQGCSGPGPEDCVK
CRNYQIILDEETNKFVCNSSCPEDRNHIFHGMCLTAEQNARLSGQTARELRNRILIGVSVSVFIIIALVTIILVVCLKRKAEA
EKIREQLRSAYTNLLEPDMKTQSVSREPNMGRLEMINQDDLFCDFNSAPLGTGSFGAVYKGVWKVPKHALLRYNWHRGAQLDV
AIKVILNDSPECSVTANPSSPFEAGNSSYSEEEAKRASVRANIEELLQEAKIMASVMHRHCLPLIGICLSSERHCLVSIFVEL
GALDRYVKQHADELNSLTLLSWAEQIADGMSYLEMRGIIHRDLAARNVLVQTREHVQITDFGLAKMLERRDEDSVIVKAGRVP
IRWLAIETLQYGIYSHKTDVWSYGVTLWEIFTFGKRPYEDVDTVDIKDHVIKGGRLTQPDICTLDVYMVLVKCWMEDYESRPT
FIELMRTFNTFCKTPGRYLYIEGDQYAINYFHNTNSGSGNFSSESHELQPMLSVRGIPDGGTTPHRNNSLHRHHTMLTEPSMT
RPYSSGKLLRALSDQPSERSDLFSVGQTGEHTETQLLLPIRSNNSNSGNHSTWQSRQHGVGTGASSNTSDTNFSGLGRIWKRS
NFHDKVNTDNTSLLKQRKAPLASREDSWLNDIPRQSDHSESRSSVALSDPATASTTAKTSEWSGISPYNQSRNHSNFGGKNLE
EMNTKFSPHTHGNSSNSFGKNTVSDYLLDPPPPPPVPRGLPDEYLQPKTKNPTNTSHAYSSALSKSMNYTELGPPMVTNPSTT
RDEYLSPNMQPPEEYLSPISGGFSVTNPEYLMETYGHPQQYPEPNTLKQSTNPASDSNSPLKFQDKTNQK
>Schistosoma mansoni EGFR-3
MKNFNPNCTHILGNLIISGVDEDDDVTLLENIEEISELRLSHLKLIKGHEWIIFKSRKVALLVVSNYKQNDLLSSSNVSSQST
SSVNDTGKQRYGLSHLQSLEMTSFISVVQHGIYFYDNPGLCYTPFTLKWDEMIESSELQTIDLYALHSNDNITLCYENTTSSI
TVISNERIHDDNHTVKMNLSKIDNKSIMNYSMEYPSLPCHTSCPLIQGKRYCWGPGRDQCQAEQCCHSECAASCYGPRARDCN
ACLRLSNRGVCTDSCPASKKYNKSTFSWVENPDGLLTFGMICVKSCPKTYLRDDDHCVSKCARPGYMTYQGECIPCPNSICPK
ICTLNEIESIKGIDYLHRKSLHAMENCTVFEGDIKLSMQSFIGDPFYNLSQIDEGVKWKDLIIGLSKLEYITGTLYISAGSHA
PWMTNLTFLSNVKIIGGISPNIQQTRTVNINLNHHLEFLGMTSLQKLGHPSLLITGNPRLCYVDTIDWTSLLNDETFVDELHD

KGTKISKLIKTFNTNLNNDSLAQIILRHKKDSKRKRPFKDSTIFVGGNAHFEFCKGRGAVCDELCQPHSGCWGPGRQFCTLCK
YWSIENIDNGGRICMEHCEDLPGFYTPKLNDTHSNLEQILNNRSNIVIDVQNLSTAVYNNEINKSTTELINKYYSKENKIFSS
AQCKKCSDMCSLQNQTCYGPNDDQCIGLCRFVQDGPFCRAYCPPNKYTDPITKKCMECSSTCTPPGDIENGNIYINNNLLNQK
IENKLKYYCTGSGDWPGNGGCSFCKQTVLYLEPKNSNHHLKCVNSCPFGTYQHVINLHHRGGSSVDRQSTVSFNIKEQNSSMN
RFTWSQTDANLFHNFPSKIIDEISLWLLNHTQPQLYGLAQICLPCNEQCHSEIISSRQSSLIGSSNVACYGPSPEQCVRCAYA
SYQGRCVSTCPSGTYPKKKSLRNFDPTLLNDYYIGYLPDNITSFECLSCHKECEIGCSGPTAEECTRCRHVKIYHDSQMKSWL
CNNTCPDFSPFKIFDQKTGEIICSSDSINLHIIPYYNGQYSIENHLNSTILHNSYNNNIVTMATKNLNFLQNIHMMNYNNIWH
YHYLSTQTAGLISSLSALFIMLLLLSLIIYWTIYKRSKYIRIEQYDTTINTTDNTTNTTGTNNNNNNKGNKSCFISLNKICEK
FWTQGKPQLVIKKKKKEKNKEKRKKSKTFTIATKNHRSILNYKNNQLIKEKLLFHPLNWNHSLIPMNGSLLLDKDNKWMNNMD
EEKSPTHSESLKPNMATLRIITESELIRGPLIGSGAFGTVYCGVWCPKFTRQKLDPINNDTYSTITNSTITPTSNVFNSKKFD
WDKNTNGTETIPGGFNNDNYFNLHIPVAIKVLSDSADPQTNKELLEEAKVMATVDHPCCVRFLALCLTSKLQLITQYLPLGSL
LEFIKIRWDFIEVNSLFQWSEQIASGMIYLSSRGIIHRDLAARNVLVQSKDQVQITDFGLAKCLDTTHSEYHASGGRMPIKWL
AIECIQDRIFSSKSDVWAYGITLWEMCTFGHRPYENIHAKNLLDFLEKGNRLPQPETTSLDFYCLMLQCWQADPNLRPTFKEL
CSALCEMKATPNRYLFITPELRTSNFTNQDTSQNALEFLTSCLTDTTISLKSTHTNISSIQSYQQIYNDLNEYNNHSLLNQLN
IEDYTQYTQMNYDWLNNLYVTLPNQYIDNININDPRDNSIHHCNDILIDAEETNRNEENLVTKESEGSFMEESSENVSLSILQ
MDSAQISDQYYVNTVIEKNNCNEVSCIVIL
>Echinococcus multilocularis EGFR-1
MNEISGFPIDGTYEYLRKLFSSGCTHIVGNLIIYGLHLNVDGLGPDLDFLKHIEEISGFLIIMNSSVEEIPLSGLKIIRGLGS
GYSLREDLPKASLLIRHTYDSGGILRKVDLGNLRVIQNGDVYVLDNPQGCNLTPGISWSDLFANPAVQHFHNSISSDPNPDDA
SHVESCPPLPARGRCKKRNCEWCYETKGASSEEHCCDTSCLGGCTGPTNADCFVCRSLRDGNRCVDKCPPPSTVQNYRLTLNV
NFSYEFNGFCVKECPKSLLTEGTKCVSECTPGHYYVLGHFCIPCHTNCPKVCSIGKFSSLDAATLASLENCTTLSGDLIISEE
SYNSELPGRKPITTTEELWALHSLQEVTGFVHLDLRNHPEPLKSLTFLENLRKIGGSNSRFSMAVAHGEIEFPGMHRLREAPH
GAVAFMESPALCYIQTIVTTKTTDRYRFLPNFRIQMYKIPTAAECEARGAVCHPACDSTFGCWGPRREDCLLCRSHSVQGFIC
VESCTELPGFFEASRENVTIIPTLPPQSNPMTARLQMARMAAGHIIEEHIYSRYGDYSEDAKAPITCARCHSECAETCTGPGA
DQCTGSCKNARSGNECVVECPHTTFLDTERRECIPCPSLCHQRKLSSKPVCTGNGIYPGPGGCNKCEMFLEFISFDGLLKDNS
DRLSKSSMRTQHGALLCMRGACPKGTFRTMETVESHSRFAQFVEEGVYTVPVCSTCHVLCRTCTGYSVLRATNTTPGCLECHG
FWFKDTCVKECPEDMTFQISLTHPPEARKAFHFRQLLDGSGLFLPLRLSSPVRGTDAFPRPHLWTRHLNGHCLLCHPECLAGC
WGPSASHCHRCSHVRVWRSGGHQLPGATWHPPNDVATVYLLNKEAFDPLQSELRETDSSHKMTSMYFTCEAACPEEMPYVSYD
PLTGDKTCHSSADIDSAYADRRRGEFAIISSRLAAGLVGPLAASFLIFLICITLACYYKGRHKRSSNSFTGKLNAFSFSLIID
KVLRLYEGSKAGHSSRVVTYTVGSGSGMDRSEIISSNDSSSEAHGPCLDTAVNRVTWFSEGSFCQYCRDGSCSNSCDDVKSPP
STRVYRSTHWSNPQMYRRSTRQQPNMGRLVLINLDDVLLPPIKVALGSGAFGAVYKGVWRVPHDIEDIHLPTFEGRDHRRHVN
VAVKILSEVNGPSDLHALLEEAKVMCSVRHRHCLQLLGVCLAGSQRYLVSEYIANGSLDGYIRRSRNDLPHWLLLLWAEQIAE
GMAYLQSVGIIHRDLATRNVLVKEREWVQITDFGLAKMLNGEEEEIGEVIVQSGRVPIRWLAIETLTSGRYSFKTDIWAYGVT
LWEIFTFGQRPYPTIETKDVKRYVLTGGRLLQPDICTLEAYQRLLLACWRENPDERVSFDDILQLLRSKMDMPEYFLHSRQAN
SVISTQTHTTRLSGAYSEAARRDRDYASSPPNALSTVASSSFSSFPLPMATAASSACQRQQRSTLLSQLPPRFPQNPIETQDS
LPGTVEYTTATNSRNSSSADNSSAERQESPIPAGSGGYVWPVPFTEHAEGVTGGGEYVGPLFQRSTDATDL
>Echinococcus multilocularis EGFR-2
MNAKTLLAYYKRQYSQCTHIIGNLVLCNLKRLEDGSDPDLSFLNSIREVSGYVYIGNNEVKRIPLISLRIIRGRVPYHVGNVG
DGALIVTRNAKNYTHGLEVLDLRSLAVIQEHNIIAWDNPMLCHFQYTVDCPQLFVDVKNQRRLSVSKENLISGSGCDYGNTTC
HSACSENDEKGHCWGPEKNQCQLRSKCTKESTTYCRLDNPFERIKCDDACIGGCNGSTSNCWACRSKRNGDTCVSFCPPRHHV
NPTTSRQELNPDFKYELHDICVRQCPEPLLKMDTVCVIECDFKTTIPVNGSCVPCAKTPCANHCEQKDIFGSRFPLMTSAAAK
RMSQCVFYSGAIYINKDSFKVTLKRPGLQIEDLWNLHNIREIVGYIYLDLDASSPELRNLTFLENLYKVTTEFSDGVSLDQPL
VIYHSKYLEFLGLKSLRYMDMTAYLEALPSLCYTSALEKILPVRTKGVKDPATCIREGHVCHSECLPEAGCWGPGPAMCAHCC
TFEANGVCVSDCSEAPGFYLPPSSAVSTLTSKFRCRTLPLSMKQVAQMQEEEVLAAIRPAVKCARCHEECAESCTAPGPDQCL
GECKHFKSGDTCVKRCSKEQYAEEKQKLCQPCNSKCLASLKSKFPPDDISACSGPGDYLGLGGCAFCYFVKKDKATSKYQCLD
MKCPPKHFGNATSLTHLNLKKEEFPLVSVRAANADLQECVPCHPECEVCVGPGNHVSVCRKCRNWMYRSECVNACPPDDTYVP
NATATEHLSEREKQMLERRECLRCHEQCAGGCTAYGPEFCNNCRYAKIMIDVQANKFICNSTCPPELYKMENTNLCLDEEQYE
KMSGAKLARARNQALIAAGIVLLFLLIFSLVLSVLCFNYKAKRSRIKEALKSTYTNTKAPDMKDAKSSREPNMGRWEMINIDD
LTFDDANNPIGRGAFGEVYRGKWRVPKRVLNQFNWARNTSLDVAIKVIRSAAPTAQGGAASNLGASGLDGNVSTVASSQLGAN
TIDRISARSNLQDMLTEAKVMASVQHKNCLPLIGVCLTRKMQCMVSMFVEEGSLDRYLRLHRDDLNSFTLLSWAEQIADGMAY
LEERGIIHRDLAARNVLVQSRELVQITDFGLAKMLDTNNEDSVVVRTGRVPIRWLAIETLQAGIYSHKTDVWSYGVTLWEIFT
FGKQPYENISTSEIKDHVMKGVRLSQPEICTLDTYMVMVQCWMEEHESRPTFLELMKCFHKYCQTPGRYLYIEGDEYAITRAS
PYTPSPAPWTEMKPLPSSFRGVPDGTLGPPNGTGCAFRFPEEHFFDDHQHHLLGETMGEGSGNPEAESLLPQRVGNDHHHHRR
FFSHSNETRMRQSGRQERAPAAGGGASTNTSVVSTNLDSWPAGSGMGLNAAFPVLEHSRNQQQQPKYYNTAFLASGTYGPLVR
PPSPPPAPPPLAQVERDDDLDTMGSGIKDAEINYLSTLEMSMDGVEGAGGGEQSLQPEDYLEPRASADGKKRSRGFNAVGNPD
YLVDNQEYFQPAAFTEAMVNMASPRPFYPSFRLFLICSRSPFPACHPAPNRCLTAFIETCRRHTHQLTSLTCAKQLHFY
>Echinococcus multilocularis EGFR-3
MLSLHLLVLLLCFIRMEAMGAEDFPTIDEAELITHELINPFTPYFRDPKYHGKIICKGFSTWQDALFGNYKKLKKKFARNCSH
ILGNLIVSEIRPNEDVEFLNTIEEVSGYVFIRRVTKPHFALPNLRIIRGEESVQIRGQAFTLLVTETYTSDRDFMSDIEFPSL
EAILMNGVGFFDNPGLCYAPFSVNWDDILEYPDLQPVMLVPMNSRSATSHWITACATRYGNDSKVSILTTTPSSSVNMSNGER
STAIQSSRLRRKSNEDVITTVALDNAQPSHAMELMTNDQPIIRGFSTLEINPIEVVSTEVMDNVTQNDDENPKVSPETTHQEQ
ISTSIALDSEKISSEFGEMSTNEMYPVLWNLSSGNGPWVKWKCHDNCSKQNGRSFCWGPAADQCQILRKCQTRVCSGPNRCVK
EGGVEECCHDECVGGCNGTRANECLACRRYSHEGNCVSACPVTRFYDVHTSSWKVNREGRVALGHMCVSKCPDGFLRDGDHCV
LKCSRPGTQQVHNVCVQCPPTGCPKTCSGKELQLPSVDFLHKSLLRKMVNCTFWEGSIILKRQSFEGDAFHHLTKEEESVTFE

DLQSGLGSLFEITGVLYVGTDNTAPWLKNLTFLSKLRHIGGEVPPGYTSPLKIVHNNYLEYLGLASLVYVGVYRGTIQIVQNP
KLCLADTVNWRSLMWAPKTTTNTTIPIPVVVPNRTVKDCVSKGFQCDTSVCELSQGCWGPGPENCRLCAHWLIQGRGETSRCV
LNCSLADGGYYPVMITKTIDKNRALKIKSCSKCHKECGSMPGACNGPDANHCNYGCAHVKDGNFCRAECPLGKFPNEENICYE
CAAVCTSHPRSVLDGTESSNWSAVCTGPGDWFGHNGCSQCIQVVAAATTNEEHGLRLKCLTPYQPCPKSTFLHLIGGQKRKMG
DPKLAISGDIALSHVPHELQEPLGDWLSSHQLQSASINMVRVCAPCHPECQDGCSGPSPNQCVRCRNAQFNGICVASCHEGTY
EANLTSGGKQCRPCHDQCSQSCSGPLSSDCLFCQHYKRYLNKEKTKWICVESCPPDSIAHVELNSITIAVEFVCLHTGIAHHL
PPAISGKIDECLIFCTWSEIISPRLLSHLSWLSTALLIFCGLLALFVAVYFGYVCSRIVIRGRKSRSEPVVDQLQGGRSSMCS
RFWMRAVVVAELEEKKNNWRLSKSRRNISLRSLPVRSNTLNGDDEQSYLVSSQADTMPDMTTLLIIPEYQLKLGNRVGGGAFG
SVYRGIWYKGPISPEFAKKVDDLFAQAAKVELEKPKLVPLDSLVEDNAYASTEQEYLTMEEEEEEEKEAKEAQNLEKSVVLEE
KTELKLLLQVEKPITSELNITSSPAPQTGMFRFSEDCEEEREKFYMENVEEHVVAVKVLTDETDPFTSKALLDEARVMATVNH
PCCLRLLALCMTARPQLVTPFLPLGCLLSYLHRHGGPNAIDCDVITPEIMLNWANQIASGMAYLASRSIIHRDLAARNVLVET
PEQIKITDFGLAKCIEDTDGEYTAKGGLMPVKWLAIECIKKRIFSSKSDVWAYGVTLWEIFTLGRRPYERTHTRHLIQYLEKG
LRLVQPMTTSLELYKLMVECWQGDPDRRPSFDELFQRISFMQLNPLAFINFKRVRGMKKNKQGRICLWSDSGLMGSTSSYVAT
TINTTVTTGNTSTIVKSTSEVSVPTSTSSCLNYLAEVTEEKEDVRYTSPPRDDRYGIIMPTSSQRQDPVQPLECIVEDVDETS
ALELFCFPQNDYEKPIETVQRVDPFSPNDIASAAIIAGGYSGPKEEINQAEPEEVA
>Prostheceraeus vitattus EGFR-1
MYTFHVYHLRHIFATCCLLYLLTLCQGKRNSYNDKMENSDSLFNMYQQHPKYKNFTVCKGFDSNILNTYQIHPEKNYNLLKHY
FDRNCTHVSGNLVIKHIVPINGIDPDISFLSTIREVSGYVMIYANLISEIRLPNLRIIRGEAGYPFGKEGRRYSLIVHHNHDG
RTKFLKVLDIHSLRSIINYDIDFSSNLGLCFAPFTVNWTDIFENPDQQRATYRNMRNSRISQKYCTKKYPTLKRYDDMVLWKN
NTECSESCPLYDGKRYCWGEGSDMCQKLNKCQQSKCSGSNRCYKLLSGDERCCHHECAVGCHNGLDTGCEACRLFINGDKCVN
HCPRVTIYDTKKYRHIPNPDYRLTMGPMCVKKCAPGLVKYKDACVESCDKVDHVIVKGECVPCEGICPKKCIVNDLQRKVIKE
KNLPTGIYHTLRSVFDLLINCTIVQGEISVNPLSRKDHMNSIIEEKNETLRPEDFWKLKNIREFEKSKIHLDLKSYPEIKNLT
FLQNVRSFKNTHILLAESKVEYLGLLHVREFSQSVFVFINNKKLCYLRHMRRQWKMRSKKQISLSGHNRNFCRSRKCDRLCDA
RAGCIGPGPKMCFKCRFYQAGNTCVNNCEDIPGYHSFSLQRRIADPQKCKTCHPQCKSSCTGPSADHCVGGCRNFQDNTTCVE
KCPKDTYTDNDLGKCHRCHDACMPINSFVRVKKSHYCTGPSDRPGEGGCTVCALLSFDKYGLPQSCLSECPDGTYRSPLTNAV
YKTATFDKIRPFVSPAYTTTCLPCHEDCAVCNLPGPYYCSRCKYLSYGDMCVSRCPIETTYNSTLDSNKTSSTAPAECLPCHP
LCRKGCRGPTKSDCNNCKLSSAKIFSEDGKTWMCNETCPVEAPFQIHDEISGDNVCMDTSKKDLLIEESKRRMRIIVPIVFVL
ILFVAFIILIVFCLLRQRAVAKQETIRLQAKYIGALPDEEMEPLNPSNSQPNYTQLRIINERELRRGQIIGSGAFGTVYKAQW
TPPMERDERQVTIPVAIKLLTDATDQAVNKELLEEARVMASVDNSYCMRLIGVCMTAQLQLVSQFMPLGSLLEYLRKNSASIS
SQALLTWAHQIAKGMAYLESRSIIHRDLAARNVLVQDPTHVRITDFGLAKLLDINEDEYLATGGKMPVKWLALECIKQRRFTH
KSDVWAYGVTLWELFTFGERPYHELRAAQVCDFLESGERLQQPEIVTLDVYMLMIKCWMLVEDSRPNFTELADEVKRMSRDPM
RYLVVKGDIQEPDRRSDHYETDDTTQTGDTVEMSHHTPSTLPHMVEEEDDFNNEEPETEEEDAVTNKDEYSACEGEYGEGYTP
PSYRQVCSSLRPLKTTGAEPNSPQVTTTPNHLISGRRHLGEINLAAILPTCSGSEQKYGHLEKELANKRVPTSNRRDRSRYAS
DPLDSPIHIPRARHDSFEIPEKDVLKVELSSKLVNQEEEDDGYLQPIVPNSSQSSGPNDNHWQERECVTSPHSCETATASTGN
QRSKKPSAPPLAVVQALNPDYFENGKEQYYNEAAFENTKLV
>Prostheceraeus vitattus EGFR-5
MMGQMIRIFHWIICVLAFHHIVVDCRHFRMKDYPNAKVCNGETTGDDYHYIGSAGYKRIEALYANCTHIMGNLVLTDLPGYTR
DKNGTVRTFDYSFLQSIREVNGIVRIRSVWVERLSLPNLRVIRGLKTYVTNKKSNNTLYIGMNNRQKDQNFIKYLHVLDLRNL
VSIVKGNVLVESNPGLCYIPNTINWTYILENPKEQKAITQSKEFNPQFCNKYVHNFVKRWNISGVNYVGPNGQSCHSSCPLID
GKRYCWGPEPGQCQKIIQCDRKQCEEKGGRCFNSYNVKEKRYVEKCCHKQCKGGCTGETEKDCLVCNNFINGKACVKECPPLT
FYDPYARRKLKNPNFKYAFGTFCVTNCPVYSLIKEEVCEENCDLRDNYIEGRNCLPCKAGKCNTNCFIGEIENKIKNKKHKEL
SELITTRSVLRALKGCKQIQGRLKFTGAGKAARLSNLSHEIPTYEDLMNLKDVKVWIGELHLDLYKDYPSVKNLSFLSNLETL
RSVKEGLQVSRSDLEFLGLKSLKYVIVEKMYFYVNSKLCYLENLSPYSFAFQDMLFKSIRNRKFKDCKREGKICDKNCDQKFG
CWGPGPTLCVKCMNKSADGTCTKRCSDVPGFYSDPKTTGCHRCHSQCLGTCTGPHANQCIKCRHAKLNGTCVEKCPVHMYLNE
KVGECARCSPLCAAIPGHHQCSGPLGMLGPNGCTSCRKVQVEKHPIMRIKSCIAACPKENFFEGVIDYDYLPGSNLTEKDFGP
ISPVFCIPCHPMCIACTAAGVTNCVKCRHFFYRGICTKSCPPEDTYEVPYSKNTHTRKCKVCHESCRKGCTGPTNKGCKACKA
HRIYLDAEKMKFACNASCPPNLPYPNYEEETGDSVCLKSPVATSKQTKLVIIYSGIGVTAVILVVIVIIGVVCCQRRRILKKE
HKLLEMKVLRQLEVEHVPLNETNHQPNSSHLRIIGANQVIKGIEIGSGAFGTVYVGTWRPRDEPQLDKLEIKVAVKTLKDSGG
DGFSSAATNDFLAEAHVMASVDHPFCMNLLGVCLTSQVQLISEYLPLGSLADYLPKHRHELGSHQLLSWSVQIAKGMVYLESL
GIIHRDLAARNVLLQSVQQVRITDFGLAKLMDDEQNGEYFADGGRFPVKWLAIECLRRRVFSHKSDVWAFGVTLWEICSLGQR
PYQDIRATELVDFLERGERLKQPDICTLDMYMLMIKCWLLDADSRPTFEELENDLKKKAADPARYLVIQHDHSRGPPNRSISA
ASNGSLPRRVSPNPQRSRSETPDAERGDEMGRKTLGVRGASASPAFSHLTDEVNRRKASSPLRTQVSDDSRYVVDPMLREDSN
EVFADYLTPTDDLHDSFPPRVEAYLEADRHPSHEYYNETPGQTSNAYINTELPTEDDYLSPRGMHPTVQNPEYYSEKSLSPHK
RLLNHEEEEDEDEDEGEENGYLLPSELKAVHNPQYLSSAGNRKTENAYLQVRPRSNRSTPNKDMV
>Prostheceraeus vitattus EGFR-3
MNLYKFIIICGILTGCVTISNGFSGYPGLQQNISSYKLCDGTDFPDSAQSVNKNMVYKYLKSTYENCTHIKGHLSIAGLKETT
NVTYDLSFFSSIVEVSGSVFIFGNQVSRIPLQNLRLIRGQTPFTSKKTQYSLYIAANKWYDKKPVYFDLSNLVSILRYNVYVG
HNNGSCHTESTIDWKHMLENPSQQHVIQKANMVKCPVKEVCNKTCPNINGKKYCWGPTASMCQKVSPCDRKTCMSNQARCFTA
KSGNSRCCHEECVGGCNGISRTNCYACRNFLNGRECRISCPKKDIYDSKLYQMIKNPNFRYTLGTLCVKECPKHYLVEGVSCV
KKCSVNSYPVKNVCMKCTGSKCKQDVCRIAEMAKEFLKERPDMKNDLLRNNEISRIPRSILLKLKNCTIVEGDLVFNAASPKD
GNESVLAEDFNNLSNMKRLNGKLHLYLRDYPGITTLHFLYNLEEMTSQSTKVGFELFFSQVEYLGLTKLKQLTLKNVHIMLNK
KLCYMENFDARFSNATSTKFNIKLNNNNCEKNGKVCDPSCEPSKGCWGPGRGMCVECRLWKAGEVCVSSCNDVVGYYSSGGRR
GICKKCHNQCKKTCSDTGADKCIGGCKNAKDGPYCVEKCPVIKFNDSNGICQNCARECYHDDAGLGYDRKVAACTGPSNRPGP
GGCSTCKIIQLDAKGKISRCIKSCPREGFYEASILRNFLRIAENKNLKKLLRIGGKLCVPCDPMCKLCYRRSCDLCWPQFYKF

DGDRCVKDCNDQTFRSKDPYGNKVCKPCSDECRKGCNGTASSDCFFCRRVKIYLDDKQFTFSCNGTCPKALKYTFNEADTGDT
VCVATEPAAGRRSMIIGLIIGALLIIFIIVGGIIFMCVQRHRARLLINRDKLHAKFTRKLEEEGVTLHTTDYTPNSGQLRSVS
AKRINKVSEIGSGAFGTVYKGFWNPEKEEHDELEITIEVAIKELKNTNDHTSTKEFLSEAHVMASVEHPFCMGLLGVCMSNHM
QLISEFMPLGSLSSYLPKHKHTLGSQRLLTWALQIAKGMSYLISCGIVHRDLAARNVLLQNEEQVRITDYGLAKLLDPGGEEI
HAAAGKFPVKWLAIESLLERTFSHKSDVWSYGVTLWEMFTFGSRPYGDLKACDMYEFLESGERLKQPQICTLDLYMLLIKCWL
LDPESRPTFNELVAEISVMAGDPGRYLIIENDTLRRAPLRSDEAHPSESTSPRVSNIGEKDLAKGAGVVNLPPSAGLPALESQ
LAKRGPPTSPGDHYSDNPLTSNSLPPPPPPPPPP
>Prostheceraeus vitattus EGFR-2
MDYIKLLLYGILLILFVKNVYSADGFFSKVKMCSGFKECKVGGHSSAGFNYKRLKKVLGSNCTHIQGNVIIQGLEKDKNGDDP
DLSFLDSIQEISGCFLVVANSVKRISMKNLRVIRGQQAFERKLTNNSTTTRWAVYVSANQIVSRVGGRVKYPPALSHLDLRNL
KSIQNYGVMFESNIHLCYPQYTVYWPDIFEKPNQEIKIEQDRNSIKNFCKDNSTCSSTCTPFNGKRFCWGEGADMCQIMNKCQ
RRSCSYSNRCFVNEKTKEEECCHPQCVAGCHGGTARDCMACREFLNNGECVTSCPASNIYDKQLFKYKPNPDRRLAWGPFCVL
NCTDGYYEHEDSCVASCDKINHILKGNKCVPCNDMCPKKCNLENLLKAEKIKNPDKFFDASYTTRTALRLLSNCTVLIGDIKI
MQLSKNDPYHHAKYPNETVRLEDLMHLKNVKDMTGTLYLELRSYPEITNLTFLQSLEVIRGKRFLITSSKIPYIGLPRLKMID
VEKIVLMMNPELCYINEFFLSVQHKVLRIIQHKNKNPSLCKSKCDETCNKLTGCRGPGPFLCGSCAKLKAGDQCVNKCSDRKG
FYYTDSKNSTGQCLKCHPECAVTCTGPGPGACIGGCKHYQDGPNCVKTCPLETKFANSSSGQCQDCHPSCQRISIGGNAEKRM
CTGGEAIPGPGGCTFCEYVLMKGRKVERCLSKCPPNTFRMLLSKDRVEARIAKYVRPDITKVCRPCHSECAVCETPSNYDCLR
CKNFRYKRLCLPSCPPGETYSLGLPVSGSGKVRASGTTKYTAGEERKCLPCHDFCRGGCHGPSLSDCKHCKRMKFYLDDDKTK
WNCTLSCPSHLAYKENEPSTGDTICVDKSQSLLAKESRQRTVIIASVVPATIVIIIVAIIIICCVLRTRDLARKERMRLKAKF
TGALRDEENVPLTIADTPSAPPNKSLLRMINGKELARGNIIGSGAFGTVFRGKWYPPKEREDNQQVCIDVAVKILSDVSDPAL
STEFLEEARVMATVQNPYVMALIGVCMTAQLQLVSRYMPHGSLLEYIRRRHNTVKSKALVTWALQIARGMEYLESMKIVHRDL
AARNVLVQEENHVRITDFGLAKLVDTPDGFQSNGGKMPVKWLALECIRHRRFSHKSDVWAYGITLWELFTFGQRPYGNLSALQ
MCEYLERGERLQQPEICTLDVYMMMIKCWLPDEESRPSFREIVDEVTKMNNDPSRFLVIQNDNPRRHLSSLSSNSGGYSNPSR
SHRPSQTSDEEKSAGCSYVPSCPSPLPASPSPAVDDRHRHLSAAVRRDRKAAAARYAIDPLSSSSPPCSSSAAEDDYLEPHHN
SVAGTPYVNTGAMLGDNSGDTLFDRPRIPSADNPEYFGPPGFDNSNPYYNEDVFTPSQSEGQTPHVKYANCSFPSEKAVEDSS
V
>Prostheceraeus vitattus EGFR-4
MNTLCYVFAFLVNLSCVIGFKLDGIPSKVEVCRGPSIYSGEKLANPIQWMKRHYPKGCTHVHNHLIIRNLKENDDISFLQSIE
EVNGLLIIENNEKVVISLPKLKVVRGSGAAKNGNFAVVIRNNKSPRGKYGTLDLSALRSVTNHNIFVKDNPNYCFLNAVDWGE
LLEDLYSQTVVTSKGTGCPTTKCSSSCRDKLHCWGPKASQCQEFATCSHKCSETRRCIHQTSSCCNTKCAGGCIKNEINCLTC
RAFTNGTSCLHACPPRYITIDFQQTRNPDFRFIFGPFCVEACPAGYLEFNSICIETCPTDHYVKDGKCISCRELSPDIDKKHQ
CKNNCHLTHIINDVKKKNPAILKNPKLFTSENAQSFTWKSVLKRLKNCVQLQAKEGIVINQLATSPSAGDERVQLADLYNLKT
ITAIYGSLKLNLKGFPELKSLNFLKKLEILIGGDFVVSEGERLEYLGLNRIRKMEMAAVKITKNPQLCYMENFRERLPSTTSS
VTLQSNANMVKCMRERKICSSLCKRGGGCWGPTPDMCISCSKFRAGHLCVEDCAGVTGYYLHFANSTCLPCHQQCLNCTGPRS
DQCIGKCRNVKDDGVCREKCHVMRYLNNSTGECLNCNKVCVRATGISGTGCSGPLPRPGPQGCNNCKFVGIVPNKPHIKCHLK
CPLGYFQSPIRTIEKLRLRLDGFISSDTKACRPCHKYCMSCVGPSEHECIRCKASFLKYHDASCVSNCPYDSFLIPERRYCKR
CHEQCHGCNGMTQLDCKECVHVRIYINETAGVFNCSSSCPPSHPRNEYSQKYDDRMCFPLETVSKKAIAISVGSICGFFGIVI
AMVIILICMQRRRAQLLKEREMVQSQYSRRLEGEMIPLSETDQKPNYSQLTIISAKQLVKGQRIGTGMFGTVYKGKWTPEIVT
NDVEIEMDVAIKELKSSDNARASKEFLSEAHVMASVNNPYCVHLLGVSLVSPMKLVSVFVPLGNLTRYMPKNKSEITSEIIMT
WALQIAKGMDYLESKGILHRDLAARNVLLQNIRYVRITDFGLAKLVDPGGSGSLTSKGDLLPVQWLAIECLQCLQFSHKSDVW
AYGVTLWEILTFASQKPYGTMKQREIIEFLEKGERLEQPRSSSNEFYMLLLKCWVFEEDGRPTFEELVIQIRDMLGDPKRYIS
TEEELRKIMRRNNSATTDVPDNTIHNPHTYLEVSQREDDLDGGTGIKPVSMIKEDDETDAGGYLLPDRFGPARGSNRSSCSSN
SGEIAFAPQGDGRRKHGYMAVSTEV
>Prostheceraeus vitattus EGFR-6
LDSKSTFYHKKYERIYYINETVRIEDLNNLSKLRHVRGAIYLQLKLPGIKSLHFLSNLESIQDHLGAFDVFGNGDLHYLGLKS
LKRVNVKKLRICPNNRCTKLCYMEHFKEYFQKNSSLIFVNETLIENCKTLNRTCDNECINGCWGPGPRMCRNCKNYRANETCI
KNCALIHGFYTLTSSKECAKCSEECQTTCSGSMPNQCIGDCKNFKDGNTCVKKCPMSKYPDSMKICRNCSVNCTKHEEKPSCN
GPLNYPDIKGCNFCHFVVISNERNRSAPKLQCRSEKPLVGYYTMLDTENFIQVKRRSHFLFAPCHESCLLCHGPKSTDCSRCH
NSYYYYKGSCLSKCSIGTYPVNSTNSTHNLSCIDCHVECSKKFGCYGFSNAQCYMCQDTKIYSSESSKMDWYCGSKCPVEMPY
KVVDKITNEKICSDTDPRALTAPIIVGSVSASVCFIVFLIALIMKYYKRQVRVRFERESLRAIYTRKVAHDVKLETTDQKPDP
SLLKVIGRQQILKKHLIGAGAFGTVYEGIWSIPEKPNEHSVKIPIAIKELNDNGSKKSTEEFLSEAQLMASADHEHCMKLLGV
CLTKDLMLISEFMTMGNLKNYLLDNKRKIGSQCMLRWALQIAKGMSYLNSRGIVHRDLAARNVLIERENHIRITDFGLAKLVG
NQHSFQDRGQKMPIRWLAIECIQHQVFSTKSDVWAYGVTLWEIFMYX
>Schimdtea mediterranea EGFR-1
HSRIFNYVKFDCWLLRVLNFIMVFTLFHLLILSKFIYFGDSKPNATDTSQSLTYSLIEYPEIKYCFSSMDLLDGSQSSNYNTL
KQDLGNNCTHVYGNIFINNVKSESNGSEPDLEFLSGIKEITGSLKIRNSNISSVPLRNLRVIRGDSRLYTHDSMGALQNSFEV
SIDQDISVSNSIKTVIDLRSLRSIVSGNIYISSQFCSSLANVEWNELLEDPTRQSVTGKCTPQPTKCSKKCMQKGKSGFCWGP
LTSQCQIFSKCRKRMCANRCFLSETGDEECCNSQCLGGCTSSDSSSCIACQGVLDNNVCQPECPPKKIINRETGIPEPNKYFR
PLFVNICLNKCPEGLLEQEGECKTQCTFRKYERQGDQCVPCKILCEQICTLEEIFSPNDFKYSYFSLDYPKLKKLANCTTFKG
FVQIDTESFVKHRPEIVPISNSDDLKFLENLRNISGSVYIDLKDAPFKLDSLSFLSNLQAIESLVPIISTSLTIINTDISYLG
LKSLKIIKNKAIFFKNNTKLCYFDIKKFKNIVKTDTLNDVTIISDNGMADPKLCEQRGNVCSVQCDSQLGCWGPGPNHCISCA
NVRLGTICAENCLVQPGYLTNPDSTSKVCLKCHDECAKTCSGLRSDQCIGGCKHAMENSMCVRECSRTHFLNKITKTCEPCHK
DCHSRSITRLPVCTGPSNTPGPGGCNKCQSMILRRDYQVERCYQNTSLNGYFSLNHPNTNSSPHSNFIEKFTDLIVILEPCHD
LCSQCTIGTDQRISLSKTDWKGCTQCKGYWFDDKCVSICPKVRTFLKADETNSSLGKCHPCHPQCNGCYGVDKNHCIHCNNFK

LPENENLTSFSCVESCPSNYKETSGNSNENICVFVFLSGLELILVILAVVFLFVLFTLFIIITKFCLARKGKRKRDKLKAYFS
NANESDSQTPSPPNMNHLLVISSDNLEFIMGGEILGKGTFGSVYAGKWKLDNGCFDDNSSMNSCKDSKVDVVIKVLNHSCDQN
EFMDEVRIMASVRHKHCLRLIGVSYKGSEKLLISSYMPLGSMEKYLEKNKKSLTGKILLKWAEQIANGMLYLENNHIIHRDLA
TRNVLIYKENHVQITDFGLSKIINPNKDEMQIFGGMVPVRWLAIETLRDGIYSHKTDVWAYAVTLWEIFTFATVPYEFLKAHE
IRDEIIKGTRLRQPDICSLDIYILMLKCWSVDPEERPNFEYLWTEFKNYSNNSETYLSYPNVEQNYTTNTFNNYIEIKQHEDE
ISYHAPHSPNQNRVRVIGNMRPRGNQKSIIVPEHVQRFLNGPPIRNLLYEPVNFSKTSNITLNGSFNKSNGNTNYHDFHSRPD
VSPSSLTIEYLEPLTTRI
>Schimdtea mediterranea EGFR-2
MGFVCNIIVVIILLESSLAIRQFDEEYPGVTICESSMTSGFQYFMPHSLMFLKGELGGNHNCTHVEGNLVIQNVWPDQESGDL
GFLSSIIEVTGMIFMKNNSIKDIILPNLKVIRGQNSLALEKSEVSVYIDLLDEENSQYAIFPKLRVIANNSVYFKTKVKHKFL
PQSIKWEELFENPKKQKLITYNKYMEHQCHSDCLRNDFPYCWSSSANHCQKQSKCVKEKVDNIEWCYFKMRNGEIDEGFCDEQ
CTGGCNGPKNSDCAVCKKYVNGNHCVEYCPPEFIYDRGNTKLNPNFKLRVGYMCANSCPVNLLQEVDACIYSCNSLHFYRVDN
KCIRCQNSSVCAKKEFNINKIINEENEDVFYMTAAILKNFTDIEHVIGEFHFTAQSFDKALGGITLPMLYKHLGNVQYITGDL
IIDGSNIVLKNLTFLQNLKEIRHLLTQSASAALNIMHTDLEFLGLRKLRRIFGKFEVHDSPGLCYADTLNWTGNILKNNQFDP
IYPPCNKPCDSKCDSQGCWGPGSHMCYNCRGFRAVYNCVDRCEDEPGFYTNTSAIKKYTDNYDDGICQRCHPLCLQNCTGPRP
TNCIGGCRYFKENDECVKNCSRKYFSFNKTCIKCHETCMENDKNQPLCTGESSILGLGGCSYCSKLIHKRDEKFYCYKDPCPR
HYYLNKIDISEVMKRQKSKEIGICQPCLEPCLECSGNYTIYPTCEKCNGYWSGDRCVKQCFPIALNLPRNASCPYCNGNCVER
CTVTPNDPFQCGSCNFKRLYLDNHNKSFQCVSECPQTHKYKHKDGNLTDIICMTRSQWLSYQENPKFISKWSLIILSVITVFL
ISSVYIFFRYVNKINQKKSVKYLKAIYSNIHEPDSRIILQNKKTIPNMNRLLLIPTTDLIFNINTKPIGTGAFGAVYRGVWKP
SNKDLEKSDRDSLDVAVKVIRANRNIDIQEIMEEAKVMASVCHKHCLRLIGICLKYEMPCLITTFVELGSLDKYLRKHKSTLC
SRTLLSWGEQIADGMTYLEIRGIIHRDLATRNVLLNDLEIIQITDFGLAHIVQCTEDNKNEIVISGGQVPIRWLALETLLNGI
YSHKTDVWSFGITLWEIFTYGERPYDELDVREITSYLIQGNRLAQPEICSVDLYQLMCRCWYENANDRPSFETLMSTLSCFHQ
FPTCYLNIPNDSCLRPMKKDKHRLLSQISYDFQVYPKLNNIYKQHSLDGTISTFVGDYQDDIEGEPSLKRQSSGFSWSSKQTR
SSQEIDKRLFYFRQHSAVSEDLEENDNFCRSDEQEESDSEYLEPKKIENFKL
>Schimdtea mediterranea EGFR-3
MHLLLFGVVIFISNICNGSNFNPDYPNFRICVVSEAHGSFKTPAEAYTYLKYTLGLGQRCTHIYGNLPISDLSRFKNGSDPDL
SFLESIIEISGSLSIINNDVKRIPLTNLKVIRGVTLRNEPDALNIIHNHSENGTFLEEIDLRNLRAIMSKNVIISQNPGLEYL
EKNLDWSQILDDPTTQKLKVSEFVRPSNGTAKCHTECEYWTREDGVRSQFCFGPSKNECQKQTKCVKNKDLKWCKFTKTGEIP
CSHECAGGCDGRTSKDCTVCNKLVKDSDCVSSCAPRPNHIFGMDSKDKLRYTVGGLCTSQCPASLLDQNKICVYECDFDAHQK
IGNRCIECPNGNCNSSTAGYKLCNIATIDKFYPKNNRKYSLNKQTLMQLTNCTLILGDLDIDDISFNKNGVTVPMLYHHLKNV
REIIGLFSLVTFQTKISNLTFLSNLEEIRLSVKSHTVASAMNIHGKYIEFLGLKKLKKVYGKISIGGTNGTCYLDRIPWEEIS
PDNRVKFSCNKTCDPLCTGQCWGPGNEMCYSCAYYKAGNSCVKQCSDSPGYFASQTEFKTIKCQTCHPLCRQNCTGPTPKNCG
GQCRYAIEENTCVKNCSIDFFPVANNVNAPITCQLCHSSCFVDKTLVGTNHACTGPSNILGTGGCSKCEKLIKTNNNQYLCHK
GSCPKGFYQSVINQNISHLHKNIVHQSSCEPCHSMCRTCSGSTNEKRMCHQCKGYFLDNKCVKECDNMKNVTIKLEIGDECPQ
CHKECRNGCLLANDSTACTRCLNHKLFVDDDKNKFSCVTRCPHDRNFTEYQDGDFICLKFEETINSKQRKTIFIILSGCFGFL
VFIIGFCVLYFRWRANQTRILAEIKALYSNFKEPDMKNMKEDYITRPPNMRRMQMIPISDLVFDTSSKPLGSGAFGKVYRGYW
KVPRQDNKYINLEVVIKVIHNSSKGSTDEFLEEAKIMASVSHRHCLPLIGVCLSLEKPCLVSTYAQHGSLDKYLQQNSKKISS
HSMLKWAKQIADGMAYLNKRGIIHRDLAARNVLVNDDDNIQITDFGLARLVDETDDSIVIKSGKVPIRWLAIETLLSATYSPA
TDVWAYGVTLWEIFTYAKRPYEELATGEIKSFLQQGNRLCQPEISTLEVYMVMIKCWLDDPHMRPSFMELVSTFEQFIQSPKR
FLYMPHEYVNQQYPKDEDQREMIQLNQISIDSDFRPISIRNIKSDETSWAAATLQNDSAYHLINNSYSKESDKSFLPKRKKAK
RNISLERIKNFSKFDLNKILSPKSHRESKNFNYQHSSTYDSNKTTCTSIDSGNPFYTQRSNEYDTQPVNPQIAKLRKEIKHNN
SLYDGSSKSDNTFKIPERIFIDKSLKEETEHLLQSPESRSPSLPPVEVPHVFPRTNPQFESIKSSVGNRIENSPDSGNSHNFD
IDDYLIPSRISSAGYYNLTPSPSSNVKHSMELEEVPLAVLNKEYIDDDGYLEPKKFDR
>Schimdtea mediterranea EGFR-4
MTHKTYLILFLFLIQFQINNATFEEEYPGTIICQNDNVQIESLHSSVEKLKILSIIRSKNCTHIQGSIYILGITKLESKEREL
NFDSIEEIDGHLIIEGNDLEKITFKNLRIIRGANSIKRNLIDKGHTDFSLVVSGNAHLVDLDFRSLRSINAHNVIFDSNPKMK
YLPHSIKWNELFTDPKAQILFSPTVTKDLILRDKCSDQCKLWANFTQVFCWGPSANQCQQQTLCGSKYSSECTTCRYINGEEQ
GCCNDQCLGGCNGGGASKCTACKNMLNEKKCVELCRGPYSGGIGGTITNKEFRYHAGFFCSKQCPAMMLPENTGCVHKCSEGY
FKNKSNHCIKCYGNMCTNDHLPCNLFKIAGHTDGANDFYLRAQDLVQLKNCKKILGYIVLTEDSFVKHNITVEMLYENLNKLQ
ELEGGFSYFKDNHLKNLTFLRNLLRFRAAYLHSDIPNLPSYHKFAISETELEFLGLKSLKEINGPFRFFKNQKLCYMNIPVNE
LFKNVKNFYYIPSNCTDKICHKDCSEKGCWGPGNKLCYECRKYDAAGTCVSDCSQKKGFYNPINSSLLSNQKCLRCHPECLNT
CVGPNPNQCIKGCKNKRENGTCVASCSKNFYPPLNDSKACLPCHPNCSEYNSTICTGPSSVPGIGGCNKCERILLSRKADIVS
CVKTCHEGTYAFPNDRPDISRFYPVTGFTCLPCPELCSKCKGKSPKNDECEVCKGVWHKNTCRLRCPRDESYGTKIIDSISYC
KTCHSSCKGCYGSTIFNCTRCKNKKIYLDKKKFYCNDTCPPLKSVEEIEKGSGDIVCLSLWESKAKLEETKRTHITVGVVVTM
LILVIIVIVTIVVYLKRKAIAERIETELKARYSNLTEPNSKDEDLLSKPPNMGRLMIVSKEDLIFEDESKPLGFGAFGYVYKG
KWKIPKNEETLLKKASGDFYLPVAIKVIKNMGNEEIESVMQEAKMMASVSHMHCLRFIGVCLSYEKPCLISAYVKEGSLDKFL
IRNKNKITSLIMLSWSEQIADGMLYLTKRNIIHRDLAARNVLVSRLECVQITDFGLSKMLESTKDKEIIIKTGKVPIRWLAIE
TLTDGKYSHETDVWAYGVTLWEIFTFGKVPYEDQATHDIFDFVLKGGRLSQPDICSLDVYIIMVNCWLESPNLRPDFQNLMQT
FTDYCKNPGRYLSIQGDPYEIPSPHSIEMTNFNNHYGEDQFNFPSMTAETMLPLLNGRDSIDRDVKDFTDNNEIDESLLSSTD
KEPLPTRSMNRTSNIFKLFQDNKQSQNPQNLNTRNNNTNNNNTPRLVSLASQEEPTFRNQPLITDDDPRYVADPTEKSIKMRQ
QLPLQSQFLQETPRFHSNISRENHTPFNNVSASQSPQISQISDDYLEPNAPGSKNTSLVDTGDPEGYLEPINPSNKSSLLNQD
DYVVPSLTNTPRESMLNSKPPRNSNAFKQSKQYISMDDGNPHFSNSIDDDDGYLEPEPKSRLLNSSNR
>Schimdtea mediterranea EGFR-5
MVFSCCHLSFKPMRSMLSGLSTVILLSFLILAVESMYNNQDIMFKFFDFQSYLKNKERTENHQDMELYFEAIKETFELYNQDK

RYRHGKVCKSTQTQIDVSKNSYESLKRNFDKNCSYIIGDVVIAHITNENISFLNSIEDISGYLMIFSNAVESISLIKLKVIRG
ENPLLIKGLKFSLIVGYNSMLENILFPNLQVILRHNVFFNNNPKLCYASRKINWNSLMRRSQEVVIVETNEKDCGLNSKIDEE
LSCHESCQAHLNKTYCWGNSSSACQKINDCANRTCYDSTKCYINEILKSEECCHEECVGGCTGPARSQCLACKNFNNNGICVE
NCPPEFKHDGMKYIQNPLGKLAFYYICVDKCPKKFFNEHGNCVTQCSAEKTYAVNQTCVPCPNGKCPKICRARDILDSQEISY
VHRKVLGRLENCTVYDGNILINRFSLEGDNFHNLSKQNDGIRKQDLKKLENLEEIYGYLRIHVPFNSNWTDSLEFLKNLRIIR
AENVRSHKSRGIEIVGNPALKYLGLRNLKEVRNGNIIIFSNNQLCFSNTLTNKSILHKGRFLRALNGKIENCSKKKCHSECEQ
RLGCWGPEKYHCVKCKSFVIRESKTCLSKCSNEPGYFYNSSSDHEVNRSCSRCHSECLPGINTCHGSNENECNLGCKNYKFGE
SCIKKCPAKYYVDSMKNCYPCHTNCTENSQNKESTCHGPEDSLGQKGCSICNHIIVSSNHSTSYKNSSNFNILKCLKNSDICP
KGFYKSIIDFRSGMKVNAAMQSTMEILEKWIEIGTNHELPIASVCLPCHPYCSECHGGSSWHCSLCKYFRSDNKCIQKCPKEY
FITVNETAKPESECKKCHEECSEGCLGSTNYDCLRCKHAKIYVNGSTQFFCNSTCGDRIQLFQTSEIICTDHLIYSRFNMYIY
IGGPAGIVISFVFIIVCFILSSKNQEKKFKTKLVNQGDFYPSDTGTNSGVLPNMATLLLITESQLTRQEIIGSGAFGIVYKGI
WQPNIDAIDKSNSFLKRNISKLSQDSSIKLNVAVKILTDISDPSNNREILEEAKVMASVDHPCCLRILAVCLTAHPKLITQFM
PLGSLLEFVQRNRSLINSITLLIWAKQIASGMEYLESKGIIHCDLAARNVLIQSPRQVKITDFGLAKMLDYSQQQYQFKGGRM
PIKWLAVECIRNRIFSSKSDVWSYGVTLWEMFSYGEKPFADIKAYDILEHLEKGQRLNQPKICSIDAYTILVQCWLVDPDARP
SFTELRKTFEMMSSNPNRYLLVQDEDSNEPLYVEWEEDAQKYDEFLQALVTVSHINSSYESAATKPIDRKHIKSVRDRYHEMR
AYKNCFINNENYCETDGKKHNTNSTNPDYTALLLQGKNNSNQTGSTWSDYFDSKKKRTSNCTTLTNLNLQREDCLKKEILETD
LSEDGYLIPISAYRKESAIGNPIEKFVYVCEKPAEKEMLINHESSI
>Schimdtea mediterranea EGFR-6
MGLVIPALVLLLSLRAIKPNPNHHKEINGNDSLVKINVSNFDRQASEFNLKTNNASSDSFRRADSHSDHQNTCISKLVYEDIR
DKPNNYMKVTANLNKNCTHVIGNVIITGITNENLSFLNSIEEITGFLFLFSNSVESISLKNLKIIRGENPWIYREVKFSLVVV
SNIIGENMLKYLILPKFQVILQHNILVYNNPGMLYFKNNINWNSILGKHQFLEIVEPHNSSNFTSKEIGDLSCHSSCQPHMGK
RFCWGNSSGQCQIINDCVRKTCKLSSKCFLYGELKIEQCCHEECVGGCYGPLNTQCLICKHFNNQGACVEKCPRKLVHNGVKH
IKNRLEKFTFHNTCVDKCPDKYFIENEKCVPKCKNGATYSVNETCLPCPNGVCPKDCLIKDVVQNYNIQYLNRKMLARLENCT
VYHGNIIINTLSIEGDIGNGWPKNIYGIQGKDLEKLTNLKEIYGSLEINVGSKAPWLKTLLYFRNLEIIRAETTNNKHLTGLS
IIQNDDLVFLGLQELKEVRNGIVEIVNNPKLCYTDSLTNKSILHSNQMNIYNNGKDGKCEARKCSKLCHPKYGCWGPLGMFCV
KCRHFQITEKNMCISNCSKQPGYFYNKENHQTHEMQCSKCHNQCLSSNITCYGPGADQCTFGCVNYKYNDYCTEKCPQDHYGD
GSTSNICKACHQSCTKENLFSFKRTCTGPGNHIGANGCRKCNYVQLLTNGSEGFISRFTVHSLTCIHSSNNCLDGYFKISLDF
HSPENIMSLLKLYSNNSSLKMVLENWVDRQANHEYQFGAICLPCHPLCSQCSDGTIESCHRCKSHRRGKECVHNCYEDDYISK
NSSNAESLCLKCHEECMEGCTGSTEYDCKQCKRVKIYLNSSKTKFFCNSICGDNFRIFVNSEMVCVQPQELKPIQNSWYIAAV
GGSVVSVLAVGIVCKCICMSLNKRKRYDLKLKFPNGFSTVDIESDFAPSSTKLLPIAEGQPANEHILDLGKFCIMDKGTWTQK
SNNTNCKKSCFKECLFKIIPDELVGLNVTIKVLSECSDLADNKDFLQKLKIITSTDPPWNLRIIFVVVCAYSELSKQLTTTGS
LIEFLKQNPSVVEDISILLWAHQVASGFGYIKYEGIINYDVETSNIKIESPLRIAISDSGLSKLSNTCNRMKKIKEGKLGIIW
SLKENCNDQGFSSKSSVMTYNCNSDHDTRPNLRKLKQSIEMIMKNPCSFIILQX
>Branchiostoma floridae EGFR
LKRGMVLGSGAFGTVYKSLWFPESGGNVKIPVAVKVLREGTTPEASKELLEEAYVMATVDHPCLVRLLGVCLAQQTMLVTQFM
PLGDLLKYVLQNRDNIGSQVLLNWCTQIASGMLYLEEKRLVHRDLAARNVLVKTPNQVKITDFGLAKLLDIDEEEYYAEGGKM
PIKWLALECIQYRKFTHQSDVWSFGVTIWELMTFGGKPYDGVRARDVPDLLERGERLPQPPICTLDLYMVMVKCWLLDADARP
NFSELVDTFHKLA
>Ciona intestinalis EGFR-1
YLKIVSWPGNLTDFSIFENLEQIDGFNLYKDLAALVIQDNVHANGPSYLMQIQSLGFRSLRAINHGNVYIGYLRNLCFDKAVN
WTSMIKDPQSYRGFLNGILLRKNKPASQCNVSSLCDEQCNSNGCWGFGPTQCLQCKNFSFNGTCLSTCHKEMGITFADGENEC
VRCHSQCKDTCRGIGPRNCTECLVYDHNGTCTDECPMDKYPHPVTKTCERCHSFCAVHRGDPMCFGPNNTLGENGCHRCYTAL
TTRGFDVTQCMPAGAPCPTGYFIHDSSSTLILERQTCMPCYEGCNNCTGVAREDCLHALSSSQEFTAAIVVPVAVVVCALLFS
IFFGCRYRRKQIQKKRTQSMRKLGIDPHSPEEQNMDPRVRLMEPMTPSGVAPNQAQLRIVKEAELRIGKILGSGAFGTVHKGY
WIPDLAPRERVKVPVAIKVLRDESSQVASNEILDEAFVMASCEHPNLVRLLGISLSQRIMLITQLMPLGNLLEYVRDNKDNIG
SQHLLNWSLQIAKGMKYLSEEKHLVHRDLAARNVLVKSPNHVRITDFGLAKLLDVKEDVYRAEGGKMPIKWLALESIQHRIFT
QKSDVWSFGVTMWELMTFGKKPYESVPAREVHTLLERGERLPQPYICTIDIYMLLIKCWTVDAEARPTFKELTEELSKLARDP
QRYLVIDNDGLLTDLPSPTTSEFLRSLVNDEGDDFPITDAEEYLHPQPLEGTENPLNSNQFSLQPVPTMVPWKRQHHQQHLSS
TSSQQGLLGSRGSGSGRFVHPNRSADRRLDSVMTTMTNLSSVSGSLPNGASTPFSPVAMGLNPEDDDDREVVLGATGDSLQGP
IIHYNALAAPDNFRSFSESSDAVFAPSPLIKHDRGYRTNRMDEGCSTPEQQSRYLRAREDSTTMRYSAEPVSLMRQQTKPNEY
LDPSEFPPSPFALKPPVNIFDSVRTPTSALANPEYEPSFVPSDCESGTAGNTQDTFDEDDREYQNIVPNGVTVIPDQHQLQIG
LRRGSQSEESVNSEKRDSGLQTDEEDDKKALDVKATSVANPEYDYLGDVTSGGSENPGSPGNNQNSPPSPNFVPNETEYINDS
VAPKSGPNHQNGFAEDEIFSALTLADPKMRERVLYGKNSAGRTYSEPDSGVGIEVAMDNILYHKLGPNWDTTE
>Ciona intestinalis EGFR-2
MRHCYPIVVLLVVLCLNIQTTESTSECDNVVCPEQVICQPPKTKLVLPYPGKEKCCAKVVCTCKGTNSELTSSGSREIQYSNL
EKLYTNCTLVNGNLELTGLFPFGNITTIEFTKHIEQVTGYLLIYRTIMDSINLSSLRLIRGLTQYYIGVDCRTNLTLARDQPG
YSVYANRITGDQNYLRELWMPKLTEISAGNVFFTKSRICNLESIKWRDEIFNDGSQTVEITNDPVRDRYCDPCPSVCRDDSGS
TPVNRCWGNSSDLCQLITKSKCRRQCGNDRCNAGGACCNGKCAGGCYDNMPATGNKDKWCHACKDMNNNGTCVEACPQGTQMN
PTSTITKTPKTYQFARYCVPKCPFNVPQTTSFCRTYCPEGTFDYNSNICEPCENGKCEHVCYGLGVGHGPLKDDKEINSENIK
YFNKNCTIIEGSLSFQASTFNGDSYCSIGKLNNLEMLSVFENVKEITGYLAFDEWRMHDLCLFKNLKKIRGKELKSELYSLYV
YTNDKSLRELCLNSLENIYDGGVLVVRNPRLGHTDTITWENIFSDSGNQNTMILRNQKEVPCHTMCDQSQGCWGRDATNCVRC
QYYTDYTDVNGQLTPAWVTDQTTGLAVPGYNTTTCVEQCNQEKGVYASSDMRCLPCDSECLSTCNGPTSYNCTSGCQNFKLNS
QCVPSCPLNYYIGLNKTCSACSPNCVGGCTGPLDIYGEGGCSSCHVSMDEKTSVYTHRGAKCPFCLNDQLRETCAHGCTSFNN
TGRLQCYYLPKPDITVYIIIGVMVFIFIFIVISWVFYKHRKTIKRMRQTIGETMVGERIPLEPEAVPLTPSGAAPNVAQLRIV

```
KESELEFRKMLGSGAFGTVQKAIWTPLNIQGEKVKIAVAVKTLKTSEDLLQSANNEIMDEAYMMASVECPYLVRLLGISMTEQ
VALITQLMPLGSLLEYVRNPTTRDSIRSRQILSWCVQIAKGMKYLEEKHLVHRDLAARNVLVKTPNHVKITDFGLAKMLDTKE
EIYHAEGGKLPIKWLAIECITEREFSHLSDVWAFGVTCWELLTFGARPYEGVRAVDVLSLLERGDRLPQPATSTIDIYMVLVK
CWMVDRLCRPSFSNLVDEFSKMAADPSRFVVIKHDDTDNIMSPTSVDDASFFRQLMEDEKTAEEDLIADEPDEYLLNLNHPDN
LPKYANIAHDPPQHPDQGARDARLFSRNTPSSFPESSTSSSTAGMRAEPPVTPQADPDDVFSEVVNSVKTVSPHPTSVEQQAT
NRKDSEETQRYTEDPTTKRPLLDGMQSPTTLYKPNPGEVDENNYLLPTPLKKQPDYFDPSHVPSEPQPSPLLCNETYVEGDSL
PESRPYPGQLDNGYAPICGSPQEHHDYVNTDSEARNPMWFSNEEYMATDSGIGEDVQSLLSRGNTANRGQRRSNQSQSSVDDS
EQIFLQKDAEVRYRPQQNRDSSPDSQKLNETSLNNPEYMFLNPGSSGHPQT
>Homo sapiens ERBB1
MRPSGTAGAALLALLAALCPASRALEEKKVCQGTSNKLTQLGTFEDHFLSLQRMFNNCEVVLGNLEITYVQRNYDLSFLKTIQ
EVAGYVLIALNTVERIPLENLQIIRGNMYYENSYALAVLSNYDANKTGLKELPMRNLQEILHGAVRFSNNPALCNVESIQWRD
IVSSDFLSNMSMDFQNHLGSCQKCDPSCPNGSCWGAGEENCQKLTKIICAQQCSGRCRGKSPSDCCHNQCAAGCTGPRESDCL
VCRKFRDEATCKDTCPPLMLYNPTTYQMDVNPEGKYSFGATCVKKCPRNYVVTDHGSCVRACGADSYEMEEDGVRKCKKCEGP
CRKVCNGIGIGEFKDSLSINATNIKHFKNCTSISGDLHILPVAFRGDSFTHTPPLDPQELDILKTVKEITGFLLIQAWPENRT
DLHAFENLEIIRGRTKQHGQFSLAVVSLNITSLGLRSLKEISDGDVIISGNKNLCYANTINWKKLFGTSGQKTKIISNRGENS
CKATGQVCHALCSPEGCWGPEPRDCVSCRNVSRGRECVDKCNLLEGEPREFVENSECIQCHPECLPQAMNITCTGRGPDNCIQ
CAHYIDGPHCVKTCPAGVMGENNTLVWKYADAGHVCHLCHPNCTYGCTGPGLEGCPTNGPKIPSIATGMVGALLLLLVVALGI
GLFMRRRHIVRKRTLRRLLQERELVEPLTPSGEAPNQALLRILKETEFKKIKVLGSGAFGTVYKGLWIPEGEKVKIPVAIKEL
REATSPKANKEILDEAYVMASVDNPHVCRLLGICLTSTVQLITQLMPFGCLLDYVREHKDNIGSQYLLNWCVQIAKGMNYLED
RRLVHRDLAARNVLVKTPQHVKITDFGLAKLLGAEEKEYHAEGGKVPIKWMALESILHRIYTHQSDVWSYGVTVWELMTFGSK
PYDGIPASEISSILEKGERLPQPPICTIDVYMIMVKCWMIDADSRPKFRELIIEFSKMARDPQRYLVIQGDERMHLPSPTDSN
FYRALMDEEDMDDVVDADEYLIPQQGFFSSPSTSRTPLLSSLSATSNNSTVACIDRNGLQSCPIKEDSFLQRYSSDPTGALTE
DSIDDTFLPVPEYINQSVPKRPAGSVQNPVYHNQPLNPAPSRDPHYQDPHSTAVGNPEYLNTVQPTCVNSTFDSPAHWAQKGS
HQISLDNPDYQQDFFPKEAKPNGIFKGSTAENAEYLRVAPQSSEFIGA
>Homo sapiens ERBB2
MELAALCRWGLLLALLPPGAASTQVCTGTDMKLRLPASPETHLDMLRHLYQGCQVVQGNLELTYLPTNASLSFLQDIQEVQGY
VLIAHNQVRQVPLQRLRIVRGTQLFEDNYALAVLDNGDPLNNTTPVTGASPGGLRELQLRSLTEILKGGVLIQRNPQLCYQDT
ILWKDIFHKNNQLALTLIDTNRSRACHPCSPMCKGSRCWGESSEDCQSLTRTVCAGGCARCKGPLPTDCCHEQCAAGCTGPKH
SDCLACLHFNHSGICELHCPALVTYNTDTFESMPNPEGRYTFGASCVTACPYNYLSTDVGSCTLVCPLHNQEVTAEDGTQRCE
KCSKPCARVCYGLGMEHLREVRAVTSANIQEFAGCKKIFGSLAFLPESFDGDPASNTAPLQPEQLQVFETLEEITGYLYISAW
PDSLPDLSVFQNLQVIRGRILHNGAYSLTLQGLGISWLGLRSLRELGSGLALIHHNTHLCFVHTVPWDQLFRNPHQALLHTAN
RPEDECVGEGLACHQLCARGHCWGPGPTQCVNCSQFLRGQECVEECRVLQGLPREYVNARHCLPCHPECQPQNGSVTCFGPEA
DQCVACAHYKDPPFCVARCPSGVKPDLSYMPIWKFPDEEGACQPCPINCTHSCVDLDDKGCPAEQRASPLTSIISAVVGILLV
VVLGVVFGILIKRRQQKIRKYTMRRLLQETELVEPLTPSGAMPNQAQMRILKETELRKVKVLGSGAFGTVYKGIWIPDGENVK
IPVAIKVLRENTSPKANKEILDEAYVMAGVGSPYVSRLLGICLTSTVQLVTQLMPYGCLLDHVRENRGRLGSQDLLNWCMQIA
KGMSYLEDVRLVHRDLAARNVLVKSPNHVKITDFGLARLLDIDETEYHADGGKVPIKWMALESILRRRFTHQSDVWSYGVTVW
ELMTFGAKPYDGIPAREIPDLLEKGERLPQPPICTIDVYMIMVKCWMIDSECRPRFRELVSEFSRMARDPQRFVVIQNEDLGP
ASPLDSTFYRSLLEDDDMGDLVDAEEYLVPQQGFFCPDPAPGAGGMVHHRHRSSSTRSGGGDLTLGLEPSEEEAPRSPLAPSE
GAGSDVFDGDLGMGAAKGLQSLPTHDPSPLQRYSEDPTVPLPSETDGYVAPLTCSPQPEYVNQPDVRPQPPSPREGPLPAARP
AGATLERPKTLSPGKNGVVKDVFAFGGAVENPEYLTPQGGAAPQPHPPPAFSPAFDNLYYWDQDPPERGAPPSTFKGTPTAEN
PEYLGLDVPV
>Homo sapiens ERBB3
MRANDALQVLGLLFSLARGSEVGNSQAVCPGTLNGLSVTGDAENQYQTLYKLYERCEVVMGNLEIVLTGHNADLSFLQWIREV
TGYVLVAMNEFSTLPLPNLRVVRGTQVYDGKFAIFVMLNYNTNSSHALRQLRLTQLTEILSGGVYIEKNDKLCHMDTIDWRDI
VRDRDAEIVVKDNGRSCPPCHEVCKGRCWGPGSEDCQTLTKTICAPQCNGHCFGPNPNQCCHDECAGGCSGPQDTDCFACRHF
NDSGACVPRCPQPLVYNKLTFQLEPNPHTKYQYGGVCVASCPHNFVVDQTSCVRACPPDKMEVDKNGLKMCEPCGGLCPKACE
GTGSGSRFQTVDSSNIDGFVNCTKILGNLDFLITGLNGDPWHKIPALDPEKLNVFRTVREITGYLNIQSWPPHMHNFSVFSNL
TTIGGRSLYNRGFSLLIMKNLNVTSLGFRSLKEISAGRIYISANRQLCYHHSLNWTKVLRGPTEERLDIKHNRPRRDCVAEGK
VCDPLCSSGGCWGPGPGQCLSCRNYSRGGVCVTHCNFLNGEPREFAHEAECFSCHPECQPMEGTATCNGSGSDTCAQCAHFRD
GPHCVSSCPHGVLGAKGPIYKYPDVQNECRPCHENCTQGCKGPELQDCLGQTLVLIGKTHLTMALTVIAGLVVIFMMLGGTFL
YWRGRRIQNKRAMRRYLERGESIEPLDPSEKANKVLARIFKETELRKLKVLGSGVFGTVHKGVWIPEGESIKIPVCIKVIEDK
SGRQSFQAVTDHMLAIGSLDHAHIVRLLGLCPGSSLQLVTQYLPLGSLLDHVRQHRGALGPQLLLNWGVQIAKGMYYLEEHGM
VHRNLAARNVLLKSPSQVQVADFGVADLLPPDDKQLLYSEAKTPIKWMALESIHFGKYTHQSDVWSYGVTVWELMTFGAEPYA
GLRLAEVPDLLEKGERLAQPQICTIDVYMVMVKCWMIDENIRPTFKELANEFTRMARDPPRYLVIKRESGPGIAPGPEPHGLT
NKKLEEVELEPELDLDLDLEAEEDNLATTTLGSALSLPVGTLNRPRGSQSLLSPSSGYMPMNQGNLGESCQESAVSGSSERCP
RPVSLHPMPRGCLASESSEGHVTGSEAELQEKVSMCRSRSRSRSPRPRGDSAYHSQRHSLLTPVTPLSPPGLEEEDVNGYVMP
DTHLKGTPSSREGTLSSVGLSSVLGTEEEDEDEEYEYMNRRRRHSPPHPPRPSSLEELGYEYMDVGSDLSASLGSTQSCPLHP
VPIMPTAGTTPDEDYEYMNRQRDGGGPGGDYAAMGACPASEQGYEEMRAFQGPGHQAPHVHYARLKTLRSLEATDSAFDNPDY
WHSRLFPKANAQRT
>Homo sapiens ERBB4
MKPATGLWVWVSLLVAAGTVQPSDSQSVCAGTENKLSSLSDLEQQYRALRKYYENCEVVMGNLEITSIEHNRDLSFLRSVREV
TGYVLVALNQFRYLPLENLRIIRGTKLYEDRYALAIFLNYRKDGNFGLQELGLKNLTEILNGGVYVDQNKFLCYADTIHWQDI
VRNPWPSNLTLVSTNGSSGCGRCHKSCTGRCWGPTENHCQTLTRTVCAEQCDGRCYGPYVSDCCHRECAGGCSGPKDTDCFAC
MNFNDSGACVTQCPQTFVYNPTTFQLEHNFNAKYTYGAFCVKKCPHNFVVDSSSCVRACPSSKMEVEENGIKMCKPCTDICPK
```

ACDGIGTGSLMSAQTVDSSNIDKFINCTKINGNLIFLVTGIHGDPYNAIEAIDPEKLNVFRTVREITGFLNIQSWPPNMTDFS
VFSNLVTIGGRVLYSGLSLLILKQQGITSLQFQSLKEISAGNIYITDNSNLCYYHTINWTTLFSTINQRIVIRDNRKAENCTA
EGMVCNHLCSSDGCWGPGPDQCLSCRRFSRGRICIESCNLYDGEFREFENGSICVECDPQCEKMEDGLLTCHGPGPDNCTKCS
HFKDGPNCVEKCPDGLQGANSFIFKYADPDRECHPCHPNCTQGCNGPTSHDCIYYPWTGHSTLPQHARTPLIAAGVIGGLFIL
VIVGLTFAVYVRRKSIKKKRALRRFLETELVEPLTPSGTAPNQAQLRILKETELKRVKVLGSGAFGTVYKGIWVPEGETVKIP
VAIKILNETTGPKANVEFMDEALIMASMDHPHLVRLLGVCLSPTIQLVTQLMPHGCLLEYVHEHKDNIGSQLLLNWCVQIAKG
MMYLEERRLVHRDLAARNVLVKSPNHVKITDFGLARLLEGDEKEYNADGGKMPIKWMALECIHYRKFTHQSDVWSYGVTIWEL
MTFGGKPYDGIPTREIPDLLEKGERLPQPPICTIDVYMVMVKCWMIDADSRPKFKELAAEFSRMARDPQRYLVIQGDDRMKLP
SPNDSKFFQNLLDEEDLEDMMDAEEYLVPQAFNIPPPIYTSRARIDSNRSEIGHSPPPAYTPMSGNQFVYRDGGFAAEQGVSV
PYRAPTSTIPEAPVAQGATAEIFDDSCCNGTLRKPVAPHVQEDSSTQRYSADPTVFAPERSPRGELDEEGYMTPMRDKPKQEY
LNPVEENPFVSRRKNGDLQALDNPEYHNASNGPPKAEDEYVNEPLYLNTFANTLGKAEYLKNNILSMPEKAKKAFDNPDYWNH
SLPPRSTLQHPDYLQEYSTKYFYKQNGRIRPIVAENPEYLSEFSLKPGTVLPPPPYRHRNTVV
>Saccoglossus kowalevskii EGFR
MEVIFTSDAIFPVAVLSRANQNTPHLEYVTPHHMLVKIRGCLKVPLLLLKFHFTWQACRLFEDDGACVKECPDPFIYDSITFQ
NVPNPNAKYAYGSKCVKECPEHLLVDQSSCVKSCPPGKIANDEDICEECDGPCPKTCPGFGRDELSIYDQVDERNIHIFQNCT
VIDGSLIFTSSTFDGDPFLGVVGIHTSELDVFNTVKQVTGYVSVLHAGPGQDDLSCFKNLEVIAGRDLYEGFALAVMGTTLKS
LGLVSLQEIRMGNVYIKENNYLCYVTRQMFNGILKNKNIQSASVNNNKLEATCNEDGDICNSECTNVGCWGPRADECLTCKNY
KLGEECVERCELDNGQYMVSDRQCDYCHEECFESCSWSGAENCSACLNVKDGPFCKSECPTAKYADQDNYCQLCHENCKIEGP
NSGCTGPGNSIGGDSCRSCSQVLLNKDGNLVECMTPNSACPDRHFEDYHIAGTFIDSHVCQACHEECLSCNDKGPYSCTKCVH
VRYSNVCMLDCPPGYFADDQKICQMCNEECKEGCSGSDPTDCNDCKNYKIPMNDNYQEVNTTVFSCVAECPVNKPFIMDENIC
VSNCTGNTFSNSKKHCQSCHDECLNGCYDDQRSSCFECKHLRADNGDCVLDCRPNEEESNGICISIGSPSTGARPVSSTPIII
GCLVGFSLLLILIFFVIWYFRRQRNMDERRRSYLEYTDYNLDTFTEPLTPSGAAPNQSTVRLIKETELKKGGLLGSGAFGTVF
KGIWLPEGDKVRIPVAIKVLREGMSAKANQELLEEAYVMATVQHEHLTRLLCVCMASQMMLITQLMPLGALLDYVREHKSKVS
SQHLLNWCTQIAKGMVYLEEKRLVHRDLAARNVLVESPMKVKITDFGLAKFIEINEEEYTAAGGKMPIKWLALECITHRRFTP
QSDVWSFGVTVWELMTFGGKPYEGVSARDVPDLLEKGERLPQPTICTIDVYMIMLKCWMLDADSRPVFHELAEEFTKMARDPQ
RYLVIDNDGHEPLPSPSRSEFYRSLLPDEGPELLMDAEDYLQPVSTFGHSAGSDGLGSQGYPTTYAPMAHPPPIARHESGGSN
GYDRAYVGEGAIASPTGSNGKDIMQGKLNCPTKKEDSIRYSTDPLLLLQAQKQREYFEPQEEEDEEEDAPGCLLPGNAVDNLE
YHRLSQEEEEANALHNRQPLIPMSPSNMVPNYGQRQNDRLLTTPNGGDRNNVAHSLSSDGTSPGSPPKRSLINSSMNSLDGSK
HSLDKVLNNVGSGSLSQAGSEEYLSDHDYVNDMPIRAPPPIHIDGMTSTNTMV*
>Strongylocentrotus purpuratus EGFR
MWRIQQPKRIVCTQIRGRPRVCTGTDQGTAKPYNSDNAGARQQYYEELKDRYTNCTYVDGNLEISFLHEQEYDLGFLSSIREV
TGYILILLTYSRVIPLTNLRVIRGTKLYDDKYALYVALTFHTGDPTIATEEMWLTSLHEILKGDVYFHQNNQLCYVETIAWDD
MQPGLVANISMWDEHAQRNCSEHRCHESCNGHCWGPGEEHCQILTLKDCSSTCDYRCRGPTQADCCHRSCAGGCTNSSNAGCL
ACRQFSLDNTCVETCPRRYEYDRNTFTNVENPNFRYSYGSRCLKDCPNNVLVDGDNCVKVCGPGKKEQDNQCIPCEGVCPSTC
DGIGDDGALSSKHIRSKQFDNCTQINGNLIITSHTFTGDAFAGTTPIDPSALEIFRTVKQITGYLSVQGLHQHFTNLSMFSNL
ERIDGRNLYGEPPFASLAILDNQHLEALSLISLNELKLGGAYVQNNRLLCYSEETAFDAVINPEYSVSFVNNKDRATCIAENK
TCDPQCADIGCWGPGPRQCAKCKNAIIGDTCIEACDLDNGQYVETEASASEAAVCEHCDTQCSAGCSGPEPDQCDCQSDSTGT
NCRRMCRNVQDGPFCRAECPEPKFANAQKNCTDCHSNCLKGCNGPENNLGEDGCLECPQVMLDYNRKVMECMLINTPCSDDYF
YDRISHSEKSDPLAGSVICQQCDPMCIGCDGAGPRRCKQCKLFRQDDECVDECRSGFYPDVDDMCQPCHPQCRQCFNGTVHDC
LDCESYTVQTGENTFFCALSCPAEYPNELSPYVCGKTCPTLFFPNAMSKCEGCHTECKDGCHNGTRSGCYACKNVVVNLECRA
ECPAAYKNTNGTCLYIHGPDQKATPKPGGGMSIGLLAGIVTSSLIIFIVIIVFVIWFKQRIKNDMYIDIPPNIDLHDMKSCDY
TGAPLTPSNVEPNQAQLKIIKDTELKLGPVLGSGAFGTVYKGLWIPDGEKIRIPVAIKALREVSPHAAEELLEEAKVMASVDH
PCLLRLLCVCIAQNMTLITQLMPLGAILDYVRQHKEQIGSHHLLNWSFQIAKGMVYLEEKHLIHRDLAARNVLVQSPAQVKIT
DFGLAKFLEVDSNEYKAQGVTLWELMTFGGKPYEGVKAREVPELLERGERLPQPPICTIDIYMLMVKCWLIDEDSRPTFKQMQ
EELERMTKDPQRYLVIQNDGAMSLPSPTPSDFYKTLMQDTESGADPDLLMDAEDYLQPMSLTANNMTYESQQGFFPVVSHYWI
GR

EGF-TYPE LIGANDS
>Mnemiopsis leiydi EGF
MRKLCFIVHLLCSVSFLLVGAQRPCDENTLDSTIRNFIRREYQSDVRRVLGLTTISSETIDLFPSCVERRKQFGLPEGKFGIN
KAVVSFNIEGDYSSNSGDAGYTGRLNVDCWLGRWLLTKSNVEPYLREPEGVCVTCDIRSHLPCQPCLLTCESNEIPLDDCTDC
TQSNIKTEIHTYSQPAWKAHLAASTPTTKKPDTFNQLHSTELVGTTNVVTHGVIHPNSTGAHTFAPGIETVEPKGYTTKTVKP
STKAATTLEQLFTTKIIPSTRGYTTKVIPTASTSHQNVVTTKSVNVTEGETPGFTGYNTAKFSPLYTGAFEVTTSEDPTTPES
LTTENKVVTKKVTVKTEAVATTEPVEVVTTKEKKLTTPLATEAEPTEAEQTEVVPVDWEVDNEVDQDENKNADPEYCLDRDRC
YNGGESVCERNGGDFLYCICQPGFTGTFCEENRSVAKVDDHDSSHGSFWDWKLSKYIIGALGALTKCVKSGKEDRRRMEEYNT
VGLETFIPPAPVVQTALTAAGRDRGSSDVMDERSEEEFPLHDIDSDDGLGDGPVQVHRLHHYPDPEDDGLLHHAPSHRPAPSP
DHYKVPTPKLNREYMNMNRKPNRKPNGKPNGKVVNGNYMNGHCTDHSPKFSPYMNGSANTYSHKLNDKNNDQYAHLNRSFSKK
GKPQHNYLNLP
>Amphimedon queenslandica EGF
MGIFNGDMNDDMTPRGSTEPLPLDSTLQTIHERFGITWIISDPSDSLFFYDFPKRWSTYYDPSFTPVYEPEFTDASLEEKANE
ICGNDNFCKFDIAATGRTEIGEATLNGGKIFEAIVNLSQPIICDPPCVHGACVSTDVCACGEGYEGSTCDSIVTTECIQNPCN
GGDCQYHAGSYICTCLPGLTGDFCEENIPTTTVDDADNVGLAVGLTVGILIPLVIVTMIAVIVLVLVIVRHKRSKKEIPSSQF
ASSGD
>Trichoplax adhaerens EGF

```
MLVIQSTLYWQTVTPETSLVLEELESVEATGTSEISSSGLKSCHEPKLKQELTESVYYITENNLALFDLKTNYHRIFHSKLDV
HHFYPNLSIVISSQLGNTPATQSASKLSTMVPGSIMATDSSLGTMAPSSPLAIDSSLGTMVSGFTMATNSSVGTIVPGSTMAT
NSSLVLNSSQETVLSSTVTKIPTPVQATNPPSVLTSSQETVLSSTVTEIPTPVQATNPPSVTTSMPISTTTMPPSGDLCVSRA
DCNDRGPCINITDINHKICRCEIGYFGNNCQFRDELALRKYFEDNNWRAVACIFIAISCILAVILAGLLIHSCRAKPQSASAE
IELLTKKYRREAGLNDSHSNTHSRVRQSGTRQPNLHDEHDLGVSNPTYSGTAGHHHGNGNQHA
>Xenoturbella bocki EGF
MELSTEFQTATLALCVIAELLFCTVQGAVITTPAAAVTPDQYSSNITTVATSSAMGDTTSDTTPSTHYTTCTPHYQEHWCMHG
GTCIYFVDEERASCLCPQYWDGNRCHIFEYVYQSGKQDAEEKTIAYTIAGIVFAFSLICVFVAGIVVLLRRRRARNDRTGSED
ELAPSSPGSSSLHVNTKLAAQQNGALMLNGATKNGALAC
>Meara stichopi EGF-2
MNASSLESNGTVISIDPSMAVQKLNSTQCNQQQSRELNCGHGGACFYTMQRGEPNAFCLCPQYFSGARCLQLSSEACENNTCA
TAGVLIAVIAIMCLIFVILSILACIFIAKWRRMNESLLEHKKQLQQLQHKSPSKPSVKYTPMYSTDGGGVQRSGSSASCVSNA
SSLRLLTVPTTANNIKISAV
>Meara stichopi EGF-3
MLRHKFFHPMLRTLQKLLVVFLSARCLLAVNITTDNITATDNITVNTTESTVIELSECPPEYSNYCHNGGTCQFDLIHKLPKC
YCTLYFSGIRCEDLQLSSTMLEANRGRMTIVIAVIMICLLLTTVIVGVIYLNFRRRQVARGNHNDSEAIIPRNHDISPDHSSN
ETTPTNGLKIGKRLVSNDKVPDQLRMMNGGGV
>Meara stichopi EGF-1
MVILWLYIFLISDFLHGVSSSECVVSDGPQVQFEILLYIDTSLVGDYTDVNGGDGKMLADWYISCVTDILEDTFGGQFHCVKV
LSYDRPDSNLKAMGVAQFHELSILTRVDLSTVLVEVCDKGTNFTITNITVNDLDESRMDDDTINKCDHLTTVTKNSFRTYVCE
CIPGYVRESKVSCIGVCDKNDHCKNEGECIPSLGKPVCICKDGFTGETCEEQYQLSTDGQQNAMQNVLLICLAAVTMFLFVTV
SVLSIVIIRRGRRRRAQNAVSRPRGYNSRRGANSAFLYDELGGRHPSIDGSDDSAVQLDDYELQPPTWNSDIRGAVLRATGSR
QTDKYHQHAYNPDYSNKLGRQDICTEYVDQVSALNLMEGIRSS
>Meara stichopi EGF-4
MTAPISEFFQYFLLTYWTHLQPLVMANDTLNSTVMQPTTAVIDEIMFIDCTGENANFCIPNQSLRCVQIMDTPDTPICWCAHN
FMGNRCQYYDIFSLASHTPQESQMCSCALHIALAIPLTIIATVLLSFLAVLIYDRFRKRYPVSEERVAVVNRSSYTMSGAVNV
NPRAKSQGDQELSYKSLQQSQSVA
>Isodiametra pulchra EGF-1
MTKSLAEKTRDELIALIEMLTNLLTTNELPTSNDTFIPAEIINLAPETTTQLYQTTTDVPQSSSNVRNCSESFKHYCFNGGSC
FQLVRENITACSCPLHFGGERCEKFQWYSRNNCAQLRMGDFPFGNMTELVLAILFSIAILVIVFQFIIIKNKSSKLERLKRKL
RDSTLNYDYDSFRLPKSVYNRQISAISEEKNRAAFSDRRRPVSCPSGLLTALWPTPAPPTEQTVKDVQRLDNAQGDTEGDRQT
AFDA
>Isodiametra pulchra EGF-2
MNHGMVVMLSVLFNSDVATNSISAHSKLPRPQIHRTPQQMALARNQHHSCKGDPCGEHGTCVGVDEGYTCDCEPGYVSHVKLQ
TPNCKLFHAGTCAPAMCFNRGFCSPESEGPVCTCVGGFSGNYCEVGPAAASGHSGGNGETKHWKYHRWMWIPLLVAVTGFIIV
LAVVLCRVRGSIRRVLAALGCPCCRERHASPKHHVSRETHSDEHSKLLSDD
>Priapulus caudatus EGF
MWPDKRTVHTSLQSLALLLFTATLFHDAESCSRRSTPKPRPPEATVRPNITAHTQECKEPYNSNFCLNGGSCFSIQIGNSPIS
INCVCPTGWTGRRCELKDLPDDYYIRSAQSRVEVAGLAGGVVFIILVVVCLSVGVVIYVRRKKKLKQGMQANSATDSTDAVGS
SSSRQPFSVHNTRQPQHPHPASSSAAAVKPYDNYAADISGATGNSHMGAPAARERHRDTPX
>Halicryptus spinulosus EGF
MWPERGNICTLVQSLALLLFSASLLQVAESCSRRSTPKPRPPESTVRPNITAHTQTCKEPYNSNFCLNGGSCFSIQIGNSPIS
VNCVCPAGWTGRRCELKDLPDDYYIRSAQTRVEAAGIAGGVGFLVLLVACLTLGVVMYV
>Caenorhabditis elegans LIN-3
MRKMLLFCILLLFMPQFTVSESCLPSWFRQERSAPEQLQSAENAAENSGSVPPDTSRNSLETNEIGDAPSSTSTPETPTETTI
SEAGDDEKRTEEVAKELIEKEAEYEGEYEDEKVDEEVEEALKYNEDATQDATSTLKPAVRKEIEKLKEAKCKDYCHHNATCHV
EVIFREDRVSAVVPSCHCPQGWEGTRCDRHYVQAFYAPINGRYNVRLSTMSSTAQLLVQQSSTSAIPAFAFLIVMLIMFITIV
VYAYRRMSKRSDDMTYTMSHMCPPEAFNVLKTPNGRHIPVHQIPSCSYTIPTPGTVPPNISSTPGSRIPTRQQAIRNNEQARN
NFFSILRSQGTIPSRSINDDDTPKHYKSVPRVEVSAINYSGHIDFSTVSYQSTESEVSKASVTCPPPAHTVINIELDSADTNF
RSPSRSSGEQGSPATCEPMIRHT
>Drosophila melanogaster GURKEN
MMQIPFTRIFKVIFVLSTIVAVTDCCSSRILLLREHTLKIVQHQHSHMHEHAHELQQQIQETAVELLNRLELQRKQLEASAQE
EADQLHPDTDPNPDSGGQLPNADDSIAADPEQDGIILGSSTDTWLASESSTPITDSETVTTPETVTHTGEPPPDPSSSSTPDS
TTPSPNDKETEIQMLPCSEAYNTSFCLNGGHCFQHPMVNNTVFHSCLCVNDYDGERCAYKSWNGDYIYSPPTAQRKVRMAHIV
FSFPVLLMLSSLYVLFAAVFMLRNVPDYRRKQQQLHLHKQRFFVRC
>Drosophila melanogaster SPITZ
MHSTMSVQHGLVALVLIGCLAHPWHVEACSSRTVPKPRSSISSSMSGTALPPTQAPVTSSTTMRTTTTTTPRPNITFPTYKCP
ETFDAWYCLNDAHCFAVKIADLPVYSCECAIGFMGQRCEYKEIDNTYLPKRPRPMLEKASIASGAMCALVFMLFVCLAFYLRF
EQRAAKKAYELEQELQQEYDDDDGQCECCRNRCCPDGQEPVILERKLPYHMRLEHALMSFAIRRSNKL
>Drosophila melanogaster KEREN
MRAQDLLLLATALIGAYLPLTAACSSRAIAKPRPTAAPILPPDNVEISTTPRPNVTFPIFACPPTYVAWYCLNDGTCFTVKIH
NEILYNCECALGFMGPRCEYKEIDGSYLPTRNRVMLEKASIVSGATLALLFMAMCCVVLYLRHEKLQKQKLHDSTTTTTTDGG
CQNEGMDEVDGLRPLRPVRRPFGPCRILSLEEAHLQAKASNRPRHCNELLR
>Tribolium castaneum KEREN
```

```
MEDLRPLYLLLCFRRKHECFIVEASGTTMCLSSSKQHRGRSFLNIKCFRLIEHENRRDEEEVAHVMWPAWGHPAEAAAAASPL
PLVLRCLAQALATGFHRFLARFSDACSSRTTPKPRPPAPTARPNITFHTYECPPAYAAWYCLNGATCFTVKIGDSLLYNCECA
EGYMGPRCEYKDLDGSYLPSQRRFMLETASIAGGATIAVFLVVIVCVVVYLQYKRYNKMSRASTDCVDGHSTPLNTPTFGTRW
RTSPPADIPVAVQEAGDSKEVSWNVIRSQRYTLPINRDVCSA
>Terebratalia transversa EGF
MEAVRCLIIILSVTVFIGLADGCSRQASDYTVSASKFNIPAPTIGPVVPTNLNCTPLEKKTKACLRGECYAIDLNNDRRAACR
CNSGFSGVRCERRDSWSTGQRRRREIIIYRYDDNSDDFITCCHISIGRTQHEVATAHLAGGIVGGVVFVALIIGIVFFIRWRK
QRVSNRNIQLEAGLPENNMTRTNIDEESVPMKNGKSENMNNEDELDKISDRKPSQDSENETDKTTRLIQQTENQLSRNN
>Novocrania anomala EGF
MYFVVLVFCSLAPALLGLTAPTTSNDCLPGDACQRKAAVFTPDTTESANILAPTTTTPGSTMMVEEVVSHQLNCTVDEVQSTA
CLGGGSCMAIMLSSRVSFCQCPINRDGRRCQYLRRPPRPSVEMDSVMAAHIGAGLAVAVVVVIAVVIGIYVYIRKRKRDSAAR
TVRLTDDITGVSNVNSNGSGDPDRKPMLIHKEQIEQIDKETNV
>Lepidodermella squamata EGF-1
MSPFLAQILLAIIASTNASFLSDFCFQNLFSRQAVHYRMIDNTANGGSSFFAPLMPPENAWPFTVDIRDLPETTSAAPPIATT
TLIDETQNESSTFWDNITSLPSTQAQVFKDTCSAYETKKRDCDHGHCYVLAIRGNGHNERVIMCACDPLFTGERCDIPSSDLE
STLFAQAVKTSTDSSGKLLMKGVVAGIICLVLLVLGVMAYFATHSYRQHQAFRQVVRRIFCMQADFVQHSSGHRLMEEVPASK
PARRRNPSKDDTPNDFYEMDPLTRTTAITHA
>Lepidodermella squamata EGF-2
MIYCSIGKSNLALFILLSFAPQCRCCCGVGTRESVSYQLDLTNYPTQPVFHYYNAWNVATYTTPRAEYSTATATTTTSISPLS
TSSNFQPTYAPTRATTTIGVYRLNCTDKESVERNCLHGVCYVMEARDASNTLERVILCECDEFFGGEVCDTPKTDYEAVLAAS
DKQKVPISALVTGVTVVAVCIVLSVLVIIAYFTSFAYTKCCRKPASNRRSVSQISETGIPLEQQNLSAEENAVRRRRLVAQEN
PTVVTDSVEMPLRMSRPNTGSAHVLVVPSGMVGDEVSPINSLESDRRTLISAHISQQGAHTNNYFFNASPSLRGGAAAPLATM
EPVESDNTNSPRIYTLV
>Membranipora membranacea EGF
MPSRIFTSIVMALILGDLTGACLKREIPKRLNDMKPIQPSFTMTPDLSDNITYTNITNVTTGLPSVIVILNCTGKNDSVLYNK
CVNGGYCQYELMPGGGKTSYCNCPNGYDGPQCAKRYRAPSMASLYAAVGVSAIVFIVVMVGLACVIRIRKNRSRGVQMGRSES
PRSPPPVGNSRSTVNTPQHFSLYSSPSHNGDASAAPEESIELLEQGNGHVDEQPPTYNPA
>Lineus ruber EGF-2
MVITEPALPLVVVWLSFACIADCCSRTPAKSTPPATTTAIPFSTTTAPDTHKLDCEYHERQRTACLNGGTCFALILEGTREAF
CNCPEEWRGNRCQEAELPFVFYSTQRMRTANIAAGAAVAVVVVVVILLSLYVYIRWRKKQQRMENLEFEEHAQGAIQDSNKHL
DGNSFEDRESKDGLIKQTAVDIHTSQYNNAQSDHRNGPQKTERTPNETTV
>Lineus ruber EGF-1
MTKLIAFLLCGFLLSLEALVIPEKAAARLQPPRTYTQGPSPTDPNRTKCKPKVIRTYCFNRGTCFAVKSGEKIQYGCVCPSGW
TGYRCAEVNLNMYRYMT
>Lineus ruber EGF-3
MMKFLWIYLIVIAFSEAFPRRKIGLEDGGWAEVTDINSAEMYPRKEMAKDSIRFRLKRNVDAVASMRELALALRERPNRRRHR
HGRHRHGHGERSHRRREASMLEHCGPENADFCLNGGTCFKARTIGTLICRCTALFDGPRCQEANLMGIIEVDHSASREVLHYL
ESHSIAPPT
>Owenia fusiformis EGF-1
MFSPFLLLVLVTPTFLSIADGCFRRAITQPPPSTTPIPDLENHKVECEPLEVTGQGCLNGGTCFVVKIGDNRVTNCACTEDYT
GERCQERHIPPDLLPRLHRDKMKTANIAAGVVVAVLVVFVVLLAIYVYIRWRRKRELTRDREFNASPEELGYRQPFNRRSSII
ESEHRASFRRQGTPNPSPNTNTNDVNININKDCHSPQACSEAPTGSDTTVGGGHNLTVATIEPGGGARNTHAIPSHTQPTNNI
EKSEHLETNL
>Owenia fusiformis EGF-2
MHIIRFLSELFVVTIAAGLVGATPVHQEKRDGTYNTFIKKGLRQLLYMMQKQEAEIQKQEAMNQWRFKQSRRGANNRVVIDND
NLDNAPRVTAKPIQGPVHIKVVDRHTNEFIKDHLIPLVNDKLSKSPQQHAKHRRHRTAKLHHHQSSDANRSEHVEGSGEGESQ
LARNQRDYDKITRTEPENDHNDATKPRIDKRHGVEKPKPTKRPAFLSEHLKLCEKADEKYCFNEGVCVIVGKLKTKSCYCPLG
YTGVRCQLLDIYDLFRMMRQLHHNRYRSRM
>Capitella teleta EGF-1
METGKIFLRTLVNAIDHRTRPEAVSSTSEASLDMNTGSSISMDTPDWTSSGLTGCEQWDPIYGCLRRSSQPDTTRTTTPSMDE
DPFRSPTPISSHRYPCAQEEIEQAQCLNGGTCYAIEISRKREAICNCPPEWVGTRCEEGYIDPDILGLTEDNMRTASIAAGVS
VCILVIFVLLLAIYVYLRYKRKQRLLADHEFEATADELYRRPFSKRSSIVESLSRPGSLRRGGNSPIHEGGDINMNELMSNGR
SKPMAKKAPPLAEAEDLEEETDEVVKTNKTSNGLSHKIIGQILFAIT
>Capitella teleta EGF-2
MADIRVELVDGTAAQVVTPPPFYQECFNITPAISTEMPGNNGGHRGSLCHDQTYCLNGGTCWEGGPLGRLCNCPGLYGGHRCG
EIDLSLLRSMYHKAISMDRCLRDFVGIASDQPGISELKPPENSELENHLTFVLCTDGTTANGQEAVAVIAILMAAVIILLAYI
VIKRKREVMYETHAYPCTSHTSIEAGCVNGTASFRQQTSMCSAHVEIIAQDRFVNASSQTDQTDPLPDMSITNECHTRPTCEI
NNENRHAVPLD
>Capitella teleta EGF-3
MYEGSQCGETDFRLLWPKLGHRESLCKDQSYCLNGGTCFDGGELKQFCECPNGKKGDRCQETDFENVGRNPKINRPTLVIKWT
QDQGIPESHAKDYGYTFAYTDESGDVLFGPSVAHDPQTETQNLTVSGGSKFNIYIRPYRLVDGVKKYGWPSETRSMHTSSACE
EGSFGVNCTGHCTGCKEHKCSAVDGRCSDGCHLWFTGEFCTEEPGFNLKIVITGVLSLQDLSLSTDQVNWSTMDITWTQDPEI
PPENEKYHGSTYTKNDWILSVCGLFIVVILVILVIVVILVIRVHRQIIANERMLRSMKAGKDVIQDPNMNFSVQEPDLEMGLI
SQP
```

```
>Capitella teleta EGF-4
MDFRIHSCWLPLLFYVLASSSLMTDAEKKKSFGNCKEKNKDFCLNGGTCSMVENNKPACLCPEGFSGLQCQVQDGENGGKINA
TLELKGEHIAKMDSEIQLSFNGESVFMVQLYLV
>Helobdella robusta EGF-2
MSKHMKMKPQYNSKPSHQKKVKDFLMEKHLRAVHVMEPGVRNNYADHLDNSVTLYKCGVEHYKFCGMNGICYYYSNDSNGPIR
TCQCNGGFFGDNCEFTWYYAKHFDRSAQLTLLLSVVGLIFAITILLALLFVIWRVWRIRKSRLEKQKLVACSLDDDINNINNE
LDKLTHEEKLVTTTRAALVTFPGPSKTFMKTGSVSGNRFQTAAKTTRLSSIKEKPYGQSYRRFSAPTLFDPALPESQKLEKLA
SVMGRMSRLASKTGARGDQHGGDDCMVFVSL
>Helobdella robusta EGF-3
MAIIWKISFYSLLLLLLLLLMPSFYYHHPTITLACLSKPDSTPTTTMSPFLPATTTSRGETLCTEQEKNIQKCLNGATCKVSL
TSNGGRTLYCQCLSGYEGVRCELQSSPLFRSSMAGALDAPVIVGIVAGVIVLLILTSTIIFLIVKKMRKDSRPQLDYALEPSH
QEFPPLKKPAPSSNYAISRGMTNYGSPYERSIVNQGFIPDDYIPTTKTTTAQPTKSNIYDNNNSNNHNNNNKNNNNNNNDNKK
SKPTKSTTTSASHKTSASPTQLFTISDGSGTRRSGVIITDDATDRASNASSRPTSGAGSLHYTSGLTSLHVAPVAYKETTL
>Helobdella robusta EGF-1
MAELQRHQKRCTEKENPGCLNGGLCFWYIALEYAMCKCLGEYSGEKCEHGSPPKHVVSPDPPTYSPDKSTLIIIVILLLIFCA
LALVAYFVAKLRKDYKAMSRMQDHLISPPLQQPQQPQQPQQPQQPQQLESYNSGGTVSNETKSEATTLTPRMVANTRQQHSKN
ANQNTASNASSISRAKNREKSSRSGNVAAVAAAYTATTTSTSSKTSAPSSTARVGTGNNGSNKSVRRNKAGKDEYVISNVYTK
KSCNGNNCNYGGDDDDDDEGGNDDGFDDGYANELRNNTRVDMFNNYINNKNNNNINNMNTMNNNNSYNTNNVSKKDSTTKTQK
FSKTKLMLALADANTTTAATILATGATTTTRTITSNFRLMVQQKQQKQASFQMSQHQHQPPQQQQQQTQQQQQFYQPTMQQKL
PQQQQQQQPQLVSATSSNDKRPRLAATSTTPTNGYKFSLNPTIPTTTNNNNSSNNNSSSNNCSSSNNNNNINKKLEKFENLLK
MSCNIPIRKTYDVTQKGRNIKSIADNINNDINNCNINNNNICNISSFATKFTTFFPYV
>Lottia gigantea EGF
MLLKGFFSIMQIFVNTSEIIIHYKKHCTMEGCGRTPNKPQHSTASPLDSHITTEIPTHRHPCTDAEVKIAKCFNGGECYALEI
HENRSVNCLCPKLYRGSRCEEISPDIFGELQTGGKVVIGGVAAGVCVIILIIVIVIVYYVIKIRKKRREERSHRNENGVQGET
LLQQNIINHNHTKNGIHNKETNV
>Macrostomum lignano EGF
MSPFFLLVLLLLLPSMLCLGVEDKKHQEQHGSEDQRKEASGGDGELSVLIRFSGNSFFNLIKTLSSYLSGTFRVVGASISIRV
SHFGKSIATAPTGSDKTTPAPNPTPSLHPTEKPEKPLDKIKAEDVKAAKARDAEFKKQMERWINGPVICKPPCFNGGNCTRFL
VFGENTCSCPNGYKGLRCEVLDIKRHP
>Echinococcus multilocularis EGF
MSHQTNHLLRLAFFLAVSLAGASTIRPRTASELETTDSETMNAHKDFLSLLRGPLIPAAFMNPHGARNNPRCTKSSTYVDMDD
KVFVEELPSPRIESNIREIINTFVEALKHDKSWFVSLLFQIRKEQKRRESESCSEAECIYKIPCYLSQSFRSVPMYSEMLKRA
RVREGFDLLSRRRRSLDGLEMDAEFKSLVSSGKAPVTNCTGIFANYCYNSISCIYVSVLESAACSCMPGYTGLRCDLYDLSIT
LATLSQFRTTTVDIEVLRPEDSATMYSVLDATARYTYSAFEIEDEV*
>Prostheceraeus vitattus EGF-3
MNSITTSCLLILVLTVFPSSASHRQNCPTKCIEGRCVLNIYLDQWYCRCNFGYKGLSCEELNIWAFLKAIIDDRKRRK
>Prostheceraeus vitattus EGF-6
MMGGGYHLFLDNFIFHRFTSAFSTPILYISFLIGFLIKQTAGADMHNLNFDWKACDYPHNATYCFEGSCFMKKNLDDADYSGP
YCRCEEYFTGTRCEIFYLPGKKGRLTTQQAGLMAGVIFLAVVVVLAIAVIIIFYLYKRHSSQSHNSDFLRPDEQQPMTSIENH
NHTDSVKHTTV
>Prostheceraeus vitattus EGF-4
MSVFFFQLLFFLRTATVANSFEINRYQPRLSSRPQAVNESPSPIQLPQLDEYDTDFMLQLILRRLRQHSNPLVKSLISTPSVI
PNNNRLATTARTASVPPDKYVIPPRLPPVKSPSSTPLTPTTPTSMPVPEPIDSIYADIYVPMSQVSDDSAADFDDSVTSMNQA
EPVVYCSAEDSRHYCLHGGRCVRASILEVKSCICPSGYSGQRCETVKFRPLPDEDYFDDSEEDDYFLKRSVSSSQHRPSSKLL
TLRDGR
>Prostheceraeus vitattus EGF-7
MPKCFTSVFSYLNPVCLSHKFSSKTFHRLFHLSSKIMRTRLSNYSIPLALLWYCTLLCCNLPSSSASAFIVRQTTEQANENVV
YTTQREIKDNGVFLPCNQHAAKYYCVKENSARCFALEFDDQPRCRCKRCYFGRRCEHTDFDCIIEQYFENTELLGAMN
>Prostheceraeus vitattus EGF-1
MAQWMYIVISFLIGVSHGGNNDVEDVDDEGRHALGHDGVRRMSQTEFRARLCDPEEEKFCVRGDCVVAATPDGPKRAHCICPS
GYSGEKCEIKGGSMTADIEKSANPGTCSDKDQCKLKEENAGTENEENSEQSDENQDDKQASSKDKRKGEPTGDDKDNDLVFTG
DDNKGNEKSSTKSDKESSTPGSEKENDNGTDQFTPEEFDDDEDSESNSSELETEVKIPADIIEVIECPTEVTNPCLNGGKCSQ
VTIDDYKTIVCDCPGEFTGDFCESPSNSTNVISEIDSTKTEL
>Prostheceraeus vitattus EGF-2
MKSILTSFLLILVITVVISSASNCSPRCFEGRCVHNEYSGDWYCKCNPNWKGLRCEEFYPFEK
>Prostheceraeus vitattus EGF-5
MTAVFSVVLEGAASASLNLTTTPMPYVRPDRPCPRGQHIGYCQNSGLCIYKFENGEWLGPECRCTDVMFHGKRCELFRPPGMV
TETQTLLLAGGIFIGLVIITLIICIVVGVRWYQKRASESHDHNVEQPLMNGTPNGPDSISNNNNNEDILPLKVQAQTTMMMTK
KSPVYEV
>Schmidtea mediterranea EGF-1
MITTFSSVTNCFDLTYCVHGVCTLRQLSRNSMPIRYCICSFPYSGNRCNHKNFSVFKPVQEEDTYSEIILLIISAILVLLIII
AILLIYCCILYRKNKHSYTNSVSQNHNSANVKENLPQKIHILCNETLVQSPMFNGIPATDKWRRNSYDDLINIAPLNDEFKNI
KFPRKHSDITYSLSWN
>Schmidtea mediterranea EGF-2
```

MLTSTLSSLICADTSYCVHGICTIRQLSESSLPSRFCACFYPYIGPRCNKKNFSVFKRIMEGDEYSLAALSIISAIFILLIII
IIVLIYCLILYKRKRNNPINVQGKTFMESCPQQIQIVCNEAHVQSPMMGTPSFPERKFRTSFDDFKFFPLNDEFQNIKIPRKY
SDRNYF
>Schmidtea mediterranea EGF-3
MERNCTADELLYEKDKTFCFHGNCIMKKMSEVYKEIRMCLCEKNWHGKRCTYYDPFDIENASISIKAISIISIFIFLLILLLV
FVKIGKNSQLIREKLNNLCTKKNKMKYAKPQINM
>Schmidtea mediterranea EGF-6
MYSDVQLFKLTHAHLLKVFFLLLLVAIIVPQLNPKSGNINETIVAMIRNHYFDLMSKKNDWLMKILEVVKVELXPFDKSFGGA
XXXXRCLYSIQLFHPISESRNRRSIAEQIFKDNPIFEERILNSKPRYKQFKNIKVHKTTREYLPTKPIANRMTACEGRFLDYC
QNGAKCLFIDMLKRAVCLCNKGYGGIRCEDFSLSDTVQYLGDYQMKTLDVYTLNQKNHEDICSVVDATARFTYNVISKDILQS
KPLDN
>Schmidtea mediterranea EGF-7
MVHFITLFLTFTCFINGAVLSTSRMSYNSLSLFKRSIGTSPILYINIPTKHPNTIHKFYSSMKNIFGYIYTHLLNENTQIRRP
GCLGTNCFNSKSPFENCEGRELSYCANNAQCVKVIYQERPLCRCQTGFHGDRCENQEFGTLQNMESFHEEFSNFDKEETGD
>Schmidtea mediterranea EGF-4
MYKNCSITESLDEFGEKLCLNGHCVWMNSSGTMTAAKQCFCNDNWHGKRCTYYDPFPPSLANTSIIAISIACILVLLIISVII
VFKVLKVYDSFKRPKTNNFNSLNNYQKCYQKDNNILEKQEI
>Schmidtea mediterranea EGF-8
MKMKTNITTLINVIIFGILISPITTKAILKVYIYNYQNHEDMKHDEKKCETFHWANDHCDPIFEICITEKSLCDVFKTTTNVF
DNSKKIYNVFTQTFKRDKFINSISISVRVDDVDSGGISDHIAKFRTSFNIETPGLIDRKTMSVEPANVKVSLQASVSVKCALN
YYGNKCEERSDKFICNSSCINGKCNFNYQKDTFFCQCYNGWDGINCNKSIFINNLSSSTKLPTKHTSQRLTTKYNYVVYVSSF
DFSTTIIIISLIFVLCISLFSMIYVIRTTKKRNKKSSNLFTATSRPLPTITFTKNLNKESQDPIKQNTLVSQFNQPDTVYDYS
TVQIGDSYSERNSNKQNMPENEFEDPYEVPLLSHIESASSTVDDTYEIPIKSEILKY
>Schmidtea mediterranea EGF-5
MEVDCNPQESLYIDGGKYCMNGRCVMHKTSEILEAVRMCICENDWHGKRCSYFDPHHFFESSISISAMSAISILILLLILLFV
FVKVEEKSKNIREKLNKFSFKKNKMATK
>Branchiostoma floridae EGF-2
MRTTSDQILLLALCLMPGTASRAERDADGDECPDRFTGYCLRGICEYIAEADEALCLCPTDYWGPRCNHALVEQQVANDLVDV
PVAVIVGAIFVSLLVLLLVILLIICIRRYRRRRQARQRPHILLNGTHSVGVRSQISEKVNPPLLVEYMTTV
>Branchiostoma floridae EGF-1
MPDGIASDVTSTVSTAVPTISAAVPTMSAAVPGEELQVNNMSTTEAHTTHRIPCPTGSPPLGYDGFCLHGGECLYLAAFQEAS
CRCLEMYWGQRCQYHIPFSQAGQKTELRDTYIIIGVLSALLTLLLVGGIVLLVR
>Branchiostoma floridae EGF-4
MTTSVLARRLLQFCCLVCLSAIPVFSQEEPLQLELTSQGVLCGPEYDGFCLNGGECEIIPDFARPRCSCTWEYMGERCQRGNI
LAIQAMQEELRDYYIIMSAMGGVLVALMALGVGYIIYRKCGKPDPRPESV
>Branchiostoma floridae EGF-3
MVTTTVPEKHLRLMILTLCLVPAFSQDGIFYDDCPESYSGYCLNGGTCTILPGLPEDQAIRCSCPSDKVGERCENSNPNSQTT
ARGPEGEIRAVYIVVGVLGGLLLIATVAAVAYFCSQYAQSQTPNVTLPQSSNTTQFEATPQTITVHRRSTSKKIENGTGEEAT
PTTLSTGHFQTCPTEYEGYCDEGTCNYIAEHPDPKQRISCSCPPWKRGQRCQFSDPDYSRKDSVERALLNSYVVIGVLVGLLV
LVAIPAAAFICRRSRSSRDSNMNARDMREAGSSATLMGP
>Ciona intestinalis EGF-1
MYLSIPIIICVLFAGVQQSMGTEISVPCSGAEKSGFEKQSTWDASCTQVCQYTCGSTKETLTNCTTPVTPATCTGTNTCSNTT
GCTCPAQITVTTTFEQAVVRTLDCFNTSIKIVLDKCVLNYFGIKLGDLFLGTPSDLTKTQLAAVTPNDTCRGITSYENGPSYV
LDYTFAEKDSCNAIIEQTTTELIFKTAIHGLSGGSGSAVDRTSVFRLNFQCTYQRNYTKTPRGFLTPTCAANETFANGTCISS
AYFQIQLPLVTRVFDAQLNDRTSTLYAAYTTEAEKIARALFMGINMPPLQIVILGFKNGSVIIDFAVELRVNPLTPPMDVQMS
LSRYIVAVMNNQIPSSDPNVALLMQYSFGPLSVGKITATQTEILSKNPCYTKLDTCHTKAICKAISVTDYVCTCAGGYVDVLP
SNPGHVCHSPCDATNEGYCLNNGICVIEPISGSPRCLCQAEYMGVTCQDLKANEPIRRLLEILPWVAAAILLIFFGLAIVLYC
RRRAYMKKTFAKTAFMPDTRPSNSDAVVGLMRHSEQRTVLE
>Ciona intestinalis EGF-2
MVQTYIQGIFLGFLFGILMTSSTACVTDSDCPYGSCDQARGVCSCRLSCNEFQPRLACSNLRTQLDTDCRALNHGCTLQRQVL
IKTINITQYINAGRNCRNILTPWNEDQLCLDQSFCHNNGICYVSAVNSQPYCRCKSSHCGRRCQCKKPKVSATRDSTNRVLVS
ISRGGESTNQSRQAGGVGAVSHIVPYVTIGIFSCMLTCCFVVGYRKRRQLRDQCAVTDADVTSGSDELQEVTSPLQNKSEEPP
TNSQCDVIKVIVHKQSKTKETDRTSV
>Homo sapiens EGF
MLLTLIILLPVVSKFSFVSLSAPQHWSCPEGTLAGNGNSTCVGPAPFLIFSHGNSIFRIDTEGTNYEQLVVDAGVSVIMDFHY
NEKRIYWVDLERQLLQRVFLNGSRQERVCNIEKNVSGMAINWINEEVIWSNQQEGIITVTDMKGNNSHILLSALKYPANVAVD
PVERFIFWSSEVAGSLYRADLDGVGVKALLETSEKITAVSLDVLDKRLFWIQYNREGSNSLICSCDYDGGSVHISKHPTQHNL
FAMSLFGDRIFYSTWKMKTIWIANKHTGKDMVRINLHSSFVPLGELKVVHPLAQPKAEDDTWEPEQKLCKLRKGNCSSTVCGQ
DLQSHLCMCAEGYALSRDRKYCEDVNECAFWNHGCTLGCKNTPGSYYCTCPVGFVLLPDGKRCHQLVSCPRNVSECSHDCVLT
SEGPLCFCPEGSVLERDGKTCSGCSSPDNGGCSQLCVPLSPVSWECDCFPGYDLQLDEKSCAASGPQPFLLFANSQDIRHMHF
DGTDYGTLLSQQMGMVYALDHDPVENKIYFAHTALKWIERANMDGSQRERLIEEGVDVPEGLAVDWIGRRFYWTDRGKSLIGR
SDLNGKRSKIITKENISQPRGIAVHPMAKRLFWTDTGINPRIESSSLQGLGRLVIASSDLIWPSGITIDFLTDKLYWCDAKQS
VIEMANLDGSKRRRLTQNDVGHPFAVAVFEDYVWFSDWAMPSVMRVNKRTGKDRVRLQGSMLKPSSLVVVHPLAKPGADPCLY
QNGGCEHICKKRLGTAWCSCREGFMKASDGKTCLALDGHQLLAGGEVDLKNQVTPLDILSKTRVSEDNITESQHMLVAEIMVS

DQDDCAPVGCSMYARCISEGEDATCQCLKGFAGDGKLCSDIDECEMGVPVCPPASSKCINTEGGYVCRCSEGYQGDGIHCLDI
DECQLGEHSCGENASCTNTEGGYTCMCAGRLSEPGLICPDSTPPPHLREDDHHYSVRNSDSECPLSHDGYCLHDGVCMYIEAL
DKYACNCVVGYIGERCQYRDLKWWELRHAGHGQQQKVIVVAVCVVVLVMLLLLSLWGAHYYRTQKLLSKNPKNPYEESSRDVR
SRRPADTEDGMSSCPQPWFVVIKEHQDLKNGGQPVAGEDGQAADGSMQPTSWRQEPQLCGMGTEQGCWIPVSSDKGSCPQVME
RSFHMPSYGTQTLEGGVEKPHSLLSANPLWQQRALDPPHQMELTQ
>Homo sapiens TGF-ALPHA
MVPSAGQLALFALGIVLAACQALENSTSPLSADPPVAAAVVSHFNDCPDSHTQFCFHGTCRFLVQEDKPACVCHSGYVGARCE
HADLLAVVAASQKKQAITALVVVSIVALAVLIITCVLIHCCQVRKHCEWCRALICRHEKPSALLKGRTACCHSETVV
>Homo sapiens HB-EGF
MKLLPSVVLKLFLAAVLSALVTGESLERLRRGLAAGTSNPDPPTVSTDQLLPLGGGRDRKVRDLQEADLDLLRVTLSSKPQAL
ATPNKEEHGKRKKKGKGLGKKRDPCLRKYKDFCIHGECKYVKELRAPSCICHPGYHGERCHGLSLPVENRLYTYDHTTILAVV
AVVLSSVCLLVIVGLLMFRYHRRGGYDVENEEKVKLGMTNSH
>Homo sapiens AMPHIREGULIN
MRAPLLPPAPVVLSLLILGSGHYAAGLDLNDTYSGKREPFSGDHSADGFEVTSRSEMSSGSEISPVSEMPSSSEPSSGADYDY
SEEYDNEPQIPGYIVDDSVRVEQVVKPPQNKTESENTSDKPKRKKKGGKNGKNRRNRKKKNPCNAEFQNFCIHGECKYIEHLE
AVTCKCQQEYFGERCGEKSMKTHSMIDSSLSKIALAAIAAFMSAVILTAVAVITVQLRRQYVRKYEGEAEERKKLRQENGNVH
AIA
>Homo sapiens BETACELLULIN
MDRAARCSGASSLPLLLALALGLVILHCVVADGNSTRSPETNGLLCGDPEENCAATTTQSKRKGHFSRCPKQYKHYCIKGRCR
FVVAEQTPSCVCDEGYIGARCERVDLFYLRGDRGQILVICLIAVMVVFIILVIGVCTCCHPLRKRRKRKKKEEEMETLGKDIT
PINEDIEETNIA
>Homo sapiens EPIREGULIN
MTAGRRMEMLCAGRVPALLLCLGFHLLQAVLSTTVIPSCIPGESSDNCTALVQTEDNPRVAQVSITKCSSDMNGYCLHGQCIY
LVDMSQNYCRCEVGYTGVRCEHFFLTVHQPLSKEYVALTVILIIILFLITVVGSTYYFCRWYRNRKSKEPKKEYERVTSGDPEL
PQV
>Homo sapiens EPIGEN
MALGVPISVYLLFNAMTALTEEAAVTVTPPITAQQGNWTVNKTEADNIEGPIALKFSHLCLEDHNSYCINGACAFHHELEKAI
CRCFTGYTGERCEHLTLTSYAVDSYEKYIAIGIGVGLLLSGFLVIFYCYIRKRCLKLKSPYNVCSGERRPL
>Saccoglossus kowalevskii EGF
MQIKPTPTMIKENSSGKNEPTLVLDASEWEFTSLTTSTRHTLAESVISTTQSRVSAVISGRDRNYHSLSHQQDNPSLIGSHEN
LFPRRTTTTGILVRNTVVNVPLENQVIGHIGDAIRFATSVAKIDQTQQLPTSKSSGNSFNTERLSPDYFSGRIPVQPDHTTDG
QTTFLTSTDIKRHISQTSTLPSRDYIRQTVSQPGEIINDPATKHLLNETVDRPTSKPPDNNTGENATKLVNGNDERPTSKPPD
NIAVENDTKLQKENVDRLTSKPPDNIVVEQPTKLLNENVDKPTTKSPDNIVVENATKLLNENVDRPTIKPDDNVVVEQATKLL
NENVDRPTTKSPDNIVVENATKLLNENVDRPTIKPPDNIVVENATKLLNENVDKPTTKSPDNIVVENATKLLNENVDRPTIKP
DDNVVVEQATKLLNENVDRPTIKPPDNIVVENATKLLNENVDRPTSKPPDNIVVENATKLLNENVNRSTIKPDDNIVVENATK
LLNENVDRPTSKPPDNIVVEQATKLLNENVDRPTIKPPDNIVVEQPTKLSDEIVNTTTTKQPDNLNVVVEKATKLQNKTVDIP
KSVQPNNIVVEQATSLPNKTAINMTITPPDKNVEEQGTEQPNKIIERPTTKPVVNITVDHGTVLQSKTVESPTTTSKPLENIV
VEQFTNLPNETAARPTTKPPNNNYVEQVTKTDDIQTAIEHTNKSQNHSVHTQKSTLPSPIADSHTLKPSRNIVDDQNRSLPSV
RTPTLPTFLFGDKTTSSPSILISTQVHAPSEHSVDGQISVLPATIVDHHEHVLSSNQIKNASLDVTITSKTDKQIQRSLTTQV
VNKSTIPSEQTKIELTSTPRIYQTNKHNVTYITNSSYPTMPPVVGQINRISVVQSMATKSHSQTINRILPSSTTPTRNGSMVI
NAASKNTSDREHDTMLSTKQGPDHIIPSTINSLRVTSLAFVNDTHMAQERVTFEQLNSTDDLTTTTTIIDESSKKSDHYSQCE
KKAAEEFCLNGGTCRYLPELGENSCRCTRGYTGVRCSDWIPDKYLSDLLEDELMDTALGLAVAVTTLIPMGGVTAYAYYNTNI
RSQIENEYRATHLYAPGN

NEUREGULIN-TYPE LIGANDS
>Xenoturbella bocki NRG
MMFNSVVVTKQPFFAVFWIQLATIFLTMGAEGQVLTWAHSLETECTPGQDSIDPVMNTYTAAYVMEATVKDKFLDISDGSLGL
YNISVQIKRLFKGGTINATTIEIMGFGDTSPDSCFGRTLDVGQNEKWIFFLNSWSHQLSIDSSQVFSMTSGPAQSTKDVKREI
RKVACEGCAYAPKIKQRYEFEDSTVSEGEKILLKCRIDSNPPLTEVSWSKNGAPLVKGRGVSINPRSYGSSLRIKKAKLSHTG
YYECTGFNGLGEFVSTGATITVLVQQATSAMPTEPSFSVPSHTTPCPSRLNTFCSNGGTCLMALNRPTCRCPPGYIDFRCRMY
EVSQGQKQAIKSGITYAGIAVLFGIVILICVVVWHKTRRINRRRRRPRNIILGAGRVHNNHQHHNTNTKHNRTTDNNATQLQS
LSPPPDPPDKIDFVNETSFQQEAPVSWVERHRPHSSPTVNIARPRPRSLNLTGLRPIDVVTFVPEVATPLFTDDDLAGLSDLS
PGRVESEDHWGDFKSTPGVNLEDEKFQFEFDPAGDPEDSMMTPEPDSRPGPHSDPLSPHSDPCSPHGHNSGPSDPLSEADSPP
GPHSDPLSPHSDPCSPHGHNSGPSDPLSEADSP
>Meara stichopi NRG
MLFSCLFTNNKAGPTLALLFVLTCKLVECCIDITGSEFKAYGAPYVVRGFVAMRPRPTLAGERYNVSIKIEKVYKGYFEQDTI
FVSEFGNDGLDNKCRLDVRSGDKLLLYLYKSIDHNSTGGGNNYYTNYAKPEKLKRENVKKVNKVVCEDCARPLRLKQVYKFRD
SSFTVGSRWTLLCRFEGNPRPEITWRKNGIRLVSGKGGIKLVKKKTGSLLKINKLKPRHSGDYSCTAHNQVEPPLSNHLRLQV
IRQQENTTVSMLATSPPDIPIAGDYGPCSNVYHGYCMNKGSCIYLYAYEKPICHCNDNTTGRRCEFTYPKGTRRLISVSDLDK
YKNNVKMVVTYIVISLIVLFIVFIFVFSWRKSRSIRRKRGRQNLETAVPKKQMKVNGSATTPNFHNDEMVPMLEQQLDMADST
PAKLSSPTPNVPPETIRVEINDSKLGTEMYPVDISDLKETAIIDNGSEDEEEGVAGSYSVSTPQSASQSLFLPAPTTQSPDQG
LGEELMTQSDNKTAPSFSSSGIDVLNNHRTSNDDGASDSLDEVLNELENIQDGLTGLRKPPLSFRNQMAENTFGVNTIDSN
>Isodiametra pulchra NRG-1
MLLAALLLCLPLLALQSPVSTGTQTQMAVGCMEEREDVDMYHVYKAHAATNFTHGEPLQRLDTHPHILQVEKENADGSTEVIH

IDTSASDCELPESGRHLFFLHPSGSLELPGGTSVEVFAPAAKPTELDDRHRQKFDKIAKKSLCQDCSQPPRVKKIHRFPEHVT
VWEDSSFTEECKMRGDPQPHMYWLFNNQPITADLGVRVEPHWKRNVLRIDRVEERHAGNYTCVGTNFKDEPAYNTFELIVRVS
NVDDCDDSYNGFCMNGGTCFKLVRENVHACYCPKYFVGDRCQYYDPFSASVGNQSGDTRMVPLANVLIICAGIVAAASVIMAA
TCLCIAYSNERRQSHKRRQVKLQQSFDYNNRILPSQISHESANLDTKPFKSTHLNLTNSFIKGTRSVNKYQPANQEDLTPATG
QDFTPADNHFTPPDVQQNERRLHGSKCNSESIPEEPAQNHLMPNSQNRSRNPSRNQSPAVSPASRPDVDGWKLKQTSRNAVDL
SDDDVPSPMSISGHNFQSYS
>Isodiametra pulchra NRG-3
MCGISGKLPSVIMRGLIAACLLLVRLTGSSSLGMYDAGRPCMEEEYDVDRFYMYKANAAQVVMRGVVLNQESVSYDVDANLQS
FIISNASLYKGEHLFNDIEKRNLYVLFGKNRFYVNLKKSQCNTMRVFKKFQVGEEFLFFLSKSSEPDKFSMQNLPLLETPTLT
LFAQPDVYKRKIDRVLKGALFCDTCTPSPPAVTRGWDDSLLEEGQNVSFTCGLGGLPKPNNTVLFNGQPILPDQHVSLQLYGR
KVVLTLTDIKKEHEGVYTCKGTNFFDTPRQTSAYLKVIDRVEQEAANTQPCPPHFQHYCYNGGTCFWLVREQVKACHCHGIFS
GDRCQDILAPQKLIADQDEGVNVWTLCSFVLMLFILAFFVIIILRQQYRIRRMKKQFGSRVPIDFDYDIELPRQASPAPVKEV
NYSALSSQENHPPVPERSPYSLLPQSRVNHPGGPVSPDSPNHHPLPPSHQPRPVLSREEIDKLLPKYLSEPEHVTRAPDSQDS
GIEPDQRPGAVRSPGTSPIREETPLPTTPTDTYRYSKQIPQNFPQIQQQAKETDIIESDSSSGDNLPYIYPSRTANLR
>Isodiametra pulchra NRG-2
MTRFKTLKAIETEYIFYLEESVEAFWFIGQPDVYKHKIEKEMKKKLFCRSCKISPPKIQSKWEDELIVYHDSFSLKCQATGTP
TPFLSIKFQQDSRSRWEELGRSSTTGEVEWNTTVVTRGSEGLYVCEADNFADDPSSTSSNVTVTEYADCPPNYENFCVNGGRC
IWYKTADYFFCDCPLPWYGQRCYFFSVDRAAKMKDQTVTN
>Convolutriloba macropyga NRG-1
MRTAYNTLTLTVAVFAIAVWSVTNGDSIHRISQQTKSMTNRNRQFQQQIEEEDPLLSCMEERDYVKRYHVYKANAADYYVTAR
ILSDKNSTWKSFSKKHPEFLLLEEMPKETAPVKGKGVKKSGVDENDKEKDKFGDVIIVDTSLSSCEMKMKQNQYYMVYLKATK
RKTNVSIKDAKEGNDEKREKKVWIGMAEPTKYSPKKSRELVKYLTDDSNCKECQRPYMKEVHKLPPVVETKLKQKVKMECRAR
GFPQPYMYWTHEGRPMHPGKETAASVNITTTWKRSILEISYVRWQHLGNYSCVGTMFKPDIPDVTTTTQLSLSQAAVSECGPE
MEGYCLNNGKCFFSKDHNMSVCHCVEAYVGTRCQFYSPYGKRGQSVVGDSEGWGSMMVPIRDVIVICLIIVSLAVLFLISACL
YISHKNRKQKSANRKMFQSLDYSYNIAPGQLKKKGQSNGAGVNAFPSLGGFGNMSAGGPASSNRTEILLTNSFMKRPRNQNGA
VSGSKSAQEALEDLFGTGKSKKKSRGHSFSSAVVTADMGHPMLSQNTPVIIESSPNVSAKTRKEQLVLPIRIENETATAGTKN
GLHEAMNTQNSSPDGDEERSRKSDETDCTVTNEHHVGKETNNVNNNNNHHNGKQAHFMASSNVTPRHMTSPHLTTSHSTSSQM
TSQQPRDQNKGRQSSQSADRSNSRVANYNNGNQGRKVKRQNQFDDSGNGQKTYDENLSVTGSADILSDDDSPSPMSCSDMSLT
KFL
>Convolutriloba macropyga NRG-2
MKFVSTAFAVFLINSELLFITALVSSVTPSSTETEDNLDSSARITCLPERRNVNKYYLFKANAAPIVVRGRVNFSNPVEGHLT
SFKISEVAFLKGKDWITPGELKNSENLLGRNVLYVNLQKSPCREMEKSKKLRRNNAVYMFYLTKASNPDEFEFFKDSTIQVPT
MAFFAQPDLYKKKIEKTLQSELFCQDCQKVGPFVEKGWKPAYYVTEGDNLSLVCKTQGTPLPHNFISVNDTPLHSNYQDQTIN
IITKGRKLVMEFRNIKVEDAGVYSCTGTNFESEDAVTETEIHVTPVVSSSSATAVGTGSPSFPFKPSLSPSGNKSNDGIGCPE
KYEGFCLNGGDCYYLKKSRIVGCKCYGFYEGERCQEYVPRAAYSKQTIVQTRNPKFWTITTFILLTAALVSSLLYSARLFFRM
RRFKRKYEDQLTIASPTSLLPPFDYDIPPVPKRYSNIREVSYKDLDDFETKPRSIYFTPNHVRTPERRPHDSLRRPSSASEPR
RQVPVRNYPVIFENPRDEFDLPVNSPDIITSNSQTFPRSANDPSLSPTAPRTQPRLPEVPDRAFSSGIPNQVDNPTYDPKSIS
NDNSSNSSNHAPRDSFLPSLPPNFYELNKSSKETDIIDSKSDDFHEYSVPEKMRNREPIASQSSDGVYSEV
>Halicryptus spinulosus NRG
MILPASSSLASACVLLVLVRFGDSCRNDYSDVASLAYLAPLTIEASVLSVSESQSDVPINVRVKKAYFGKMPKRNIDIRPDAT
TLCQWTWVSGQKVILFLYITSVGRTYDLYAKPVLATKKNKRLLKRVACVETVGKTCVAAPVVRGLRDATKDESASVKFSCKVK
SVLPPSIAWFKDGKALVSEPGIRVKIGRKGGSVRIAKAKVTHSGTYECRATSAVGLSSKSASLTVMAAAVMTAPPPVVNDATP
TLLSMESDVMQGPRHGMYVICNTKYNGYCLHGGTCWNITDPLVNTIFCRCSPGFGGVRCDFKDPIPVLDEATRKEEQWHDRVV
TAAGLCIGAILLIVLIVVAYCKARNRKQEYRKRKEEKQRQKQRNEEVNLLKQNSANNANDNNSNQNCTTAFALIGSTKSQEEQ
ESSAEIFPPAAFKTVGPTYRRGTLTPDDPHLSVPLQSRLPGADRPDRSDRLTDVENVESFSVPSRDSYAPPRADATGAKLQAA
PRPEKSASVETQDSVDVTYGSFDQYDPVLHISEEDMRPEKQTCAATDGRNTRTNRDDVKYVAVPTEAMDPTQFSHTACLSRSR
ASSSSTYSSTTESCDSTNSSSDFIIVPPPPPAPTGMPPYPLTPPSAHWVDAGEYDGGSFCDTMFNPHAILDDSVINNKRVRTN
MAPSVKKGKIRTSMQSLDGDEIDAVHV
>Drosophila melanogaster VEIN
MYAQHLRKWSLKTKKQLMPLILLIISYMLLLNTCVLSSSATTQQQQQQQQQQQHLPRLWEGSAEESSYYIPLSSDNGSGSSES
SAESGSSSSRSSSNNIDNNILSRLLSLNSNSLSSRSNVKLKPATVFDAGSSTPAQQEQHVAAVPEQQQQQQQQQQSMQKVPNT
LINSQIYNLLYNGMPSEAASSKMRRHIQPSQLPHQPESRAQLPSNYSSRPAVRSYLIESYEMPESMLEDRSPEQAARSRRDGS
NTNGSRQQQRTGHRQQLQQDKRDHRRQRQDQQKEQRQQQQQRQHKSGNKHQQQQQQRRKHQRKHQRYNRYCSARDPAQLAFAA
PTVFQGVFKSMSADRRVNFSATMKVEKVYKQQHDLQLPTLVRLQFALSNSSGECDIYRERLMPRGMLRSGNDLQQASDISYMM
FVQQTNPGNFTILGQPMRVTHLVVEAVETAVSENYTQNAEVTKIFSKPSKAIIKHGKKLRIVCEVSGQPPPKVTWFKDEKSIN
RKRNIYQFKHHKRRSELIVRSFNSSSDAGRYECRAKNKASKAIAKRRIMIKASPVHFPTDRSASGIPCNFDYCFHNGTCRMIP
DINEVYCRCPTEYFGNRCENKWPDSRYFVAIYGQIHTLNNDY
>Membranipora membranacea NRG
MWISSRKRILLWHTARNILAIFYLLQTGVIWCCDRLDPQFDNVESKALMSEIVVSARIISLSQTRLPRRDYRSYTAAVRINYV
LKQNASTIIQRKDVIQIDGFMPNPELEPTAQSTRASSLGLQQQDNPHCLSYVSLDKSYILFLNHSTHNNSNSLTGSRRRITPS
HRYHTAQLSTEYTQSLQKKIRKVLCDTDKDGEPCARAPNAKSKRNLDISQGAYNYKISCRAEGNPPPHVRWYKDGRELDNSMR
GIAVKHREKGKQQVAILRIKTVTLEHAGTYKCIAKNVIGEDTAKTRLRVTPSSIAPKPTPTPPGSDIWDSESLPIDSEESSEE
EAPSHYIPCLERDRGYCLHGGQCEMFPDGRYKHCRCARGFTGERCMEVSILDVSRIVRQDLQALVVRRPTYSSRQRHLQRNSL
SRHRVWT

>Lineus ruber NRG
MDTKHVKHLVNVLLLLWSTQVAFCKICGSDFEDIRSRALLSDLVAEGTVDIKYENKQNPRYNVSVLVSKIFKGSLNSNTRRRV
LESIRIGEFGPLDKEQCLIGVTEGLKYVFFLRPTRDKDFYKHASLPVVSSKKTLRQVRGILCNNCAKPPEIKPIEDIIIEEGE
SVRIKCRVKGYPKPLISWAKNGKVLKKRKGVLIRNTRRGSTVRIKKLRIRDAGNYACIARNALQLTREEMFLAVSPVTSLPIG
PVKPNITSNSTKEPTHLLPTTASTRKPISITSIIRSSTSRPLLSTATTSKYSTVESILNGKESTTSSTTTTAPPITTTVAPSK
SVTDVASSQIAPTGHYYPCEKQTYCLNGGTCVYLPAFKAQFCRCPPDFTGERCETKDLFILLREIKMAGHQHQDRVITIMGIV
IGLLVLIGIFIGAYFKARNRRKALHEKQALKRMITESDEAGQFPKPNHGKKSGSSKPSMKRSNSTTQTDAFNIMELFPTMPNG
KDTGIDTTDSIQNGIDGMHRPRLLGVSTTPQRVRVEHHDNPAKVVPGESSDGGSKGSTPSQSSQTESAMQSWPPPTRRRESNS
KPNTCVDNDLNQSDYENLDSDELEGKLGVVPPYLSLESHDSYTGPRDLQSTQRNSGGAKTKADNNSKISQRKSAGLISDSGSD
SEDTSSSGAKPECEELLDKKHDATLPNKWSNANNSAGNMNWDKYGNSLSGLTANSCAFSPYDVNGYRMDNRYPHRQNPHYKLS
YDKEQRQRLLRTQDDQDATRL
>Owenia fusiformis NRG
MEYTLIIYTVLGLLITVNLGQGSTCDTDLKDVGSKAIFSQIVVESKILKKVLSDASEGSLAKYTAVVSLKKFYKGSIKNSKGK
KPKSIKLNEFISKDSEDCLAEIDSTKTYILFLNSTDKQQHFITSSKPVTSDKKTLKLIRGILKKKPSPPKVTQLKPATFEAGK
RLQLKCKIRGNPPPTVIWTKDGVTIKKKKGVSIKTNKKGSRLRIRKAKESDAGDYGCTATNVLGQFTQSAEIMVTPKPGTATK
APTTATVSPTTSSRHYEECEGEMRNYCLNNGTCGYLPKLKREFCMCPQEYTGKRCEYQDPFVYMQEVKRKEAAEKDRVITISA
IIVGAVVIIGLCVLAYFLARRRKKQYYQRKQVEAATKKRENENNFTNASYRPIVPQVNDVIVRSQPKPVTLSIMTQTEPEMFG
NHSNHLAESDSQFYQTPPPLGQQRRQFTPPGDVPKENERPRGAASVMKRPRLPAISSPTSNNNKYIVPVAVESDQEGSKRNSP
SQLSRTESSQGDPHPHKVAPAPQSVNINIDDDEPVHSDYENNMPSDTDPEPELTRLESSNEEGIKKGGAESSTEDSSDTDSSD
SETGVESQLLPHNHQGISLQNPHTEDPHSNPHSHATPNNLSWEHVPSSFFTDHEQYTANPYARETDIIDNRYTELPTQPNHRQ
TNNVRDYHY
>Helobdella robusta NRG
MTSLYLLLPQKNRPLQATEATTFLTSNFIYYCLLLLLLTTATSTLSRTNAASIDVEPSYWQNHQRSVVSPRSLCEEWRHDVAS
RATVSKLIVEGRVKKIDDSSSRASRSNFQSDKSLLYFNVTFKPRVVYKGYLPKQIYNNNVNSNSVSSNNNKNNNNYPMLVVGV
FGRKEDAGSCTPSIQLGTNYIMFLKFNPSTYDVLMTSSSSSPSYDYNADENDDGGDDDDEVHDVFDDADNDSVINVKNNDNKR
NKKSKMNAYNNSEEEDEEDDDDDGGGGNDNDDLPVGSDVRPYFEIDQFPELTSRRALRSVAEFSCKNCSSMPTIEPMAEKRKQ
VIANQKLLLRCVTHGRPAPLVTWFKNGDVLNTTVKRISVKNSRDGTSTLHIDKIKSSDSGIFQCSAKNILGDVISEGVDVIVL
TESQINDIPQYGPCDVLDYCLNGGTCHSQLKTGAKVCECRQNYTGPRCQEFNVVEHVILMRDKYEKFEKRLESLVMTSLLLIV
ILIFLFIVVIVFLTRRRFKQARTGSTTTTTTATSLICNNNNNNNNVNNNVNSKKSCNGTAVRASYIQQKQQQQQLRLSQQPQQ
PQHQQQLQMQPMKEIKVRKICNDFSSMFSKPRCNLSIRSLLT
>Crassostrea gigas NRG
MPPNDEGRYNVTLAIRRVRKGARLLPGGKKTKILTIGEFGEKDIDSECVTSITKSNQKYFFFLKKVTVENSTFYRISAFPVSV
SKQSGRLIRKAACKTCGKKPELKKIKAKNVSVGKKLQLRCRVRSARPDPSITWTKDGVPISGKTKGVQIRKKKKYSQLRINKA
SSEDAGVYTCIATNVVGTTEKSVKIKVINKPQSTGRPRTTPAPRHIPCKREDQGYCLNGGVCRIIKDLDIKSCACRRNFTGTR
CAIPNPNIREFSDAPGKTPDPERDRTLTIIGIVIGILIFVCFCIASYFLAKNRRMAYLKKRDAKKKQLNGTTTTSVYNTVDPQ
QNGRKLQRQNTVNMETQTDDGCLYPPYPNANPGWPNSYLNPPDNDFLRNPPSNRNSRTRLSGVSTERERPTSQPDTSLSSPKP
LYNRSPSDPTKPFNAADNRVCDIPRLRVSDDSVGSDGEEVADDSTSLRISEDDERTPFVRKTFSESGRFEPGGNSINNDDETV
SQSSCADDEELRRIYCADDNGRYGRRCSETLSWNERGSPEQPVQQNCFNIEDPNYLLSNNCTLPVTYDNIYNLNDSQFLDDKP
PTPV
>Macrostomum lignano NRG
MLCHRIIATSLLLLLIVGVDCRRRGRWKSIPPLDGQQECGSDFHDPQSKWLQSPLVVQARWRSADEHPADPSGAFNITVTVEA
ALKKWRRDIVGLRITVGQFNRSLGRRQRSLQRRLCSPRNLPRNGARGYILFLTPTEDANYFRHRFLPVRNTRSNRGKIARIQC
REPQHCPEAPEVTQEARLRGGSGGRRRLLCSARGQPMPRPHWLKDGVEVQQTKDCVAQFRTKSGTVMTRLTLKNYSDSAGNYT
CVFRNLNGSAASSIVLKVEPAVQREPTRRLVCDSSTKCLHGGTCTVDQLTEKQVCICPAFYTGTLCEKFDYQEGAAVYQSQAA
VAAPISSAALGVVFVAVFAVLAIVLLSVCLMLYKVRRRLDRRLRRSRLSSRCDNVETQYPSNGTATLTDVATMTETPAETLKI
EPQRQESQQPKQSQQMELHRLPDSVSTNGAHPPPRLPRTSNSFRPQQQQQHTIDYAGLPIRPIFDYSGGLHRDESADLRAELG
LLNQLERRQLQQQQQQLQPRHQQPRTMQVVEESEGVDEADAATEALLPFPGHGQRRMHHSTS
>Schistosoma mansoni NRG
MYIIILIISSSILLPMTKCIPYNVVQSNLFKQHYSCIELSKNNIEHLIQQSDLIIAGRLKYHNKNEKYKQIHSNYTTDINTTD
NTTTTTTTTTTTTTNNTTYNTTYNDNMYFNITVFIKTIYKMNHLMNNNEIIIGPVFISNSNYRNGTGCLTEFYSNAKYIFFLK
KNNNNSTVNYYHPLSEAIEYTEYIEKKIYGLYCHNCVKPTIESIPNQVLEYGHSLTTSCLATGTPTPTIMWIRDGKSINLADK
GLTVETFERSPGYVESILEINNLILIDTGEYWCYAENALGLAKEKFTLKVQPNNRTEEKLMVDELVPCSDEDKDYCLNGGQCY
TYKNERVSFQCRCLDHYFGDRCHFHTDGLYAFSESRGNDLQSILHALITTLTLRGIFFTLFGSSISVLIFYGLWKCQQLSMHR
RYLRRRKKSLFKHNSQMKTSHPVNHSNHGSRVNSTVKDSAEPILINDFHTRDRKIENIPVADDYFGQTGGSYILSAPNLVNTE
CYENRSQNFTGDVMIDLKSPLSTSHVNPQQLHDHSRNTIQFGYRPSSSLDQCNESVHKPENGITLNNRPFGTDWSTRRLASKT
SGSIRDRLPVLAEGSNLDLNVNRYPDYTDPIDRMNRFDRDREIPLNMISSSSSFMQTNT
>Echinococcus multilocularis NRG
MMAIFVLVVLFTPFQCLSGAIKAPKCAPSPKASEIRQYLSTSSIVLHARLQEMIPLKDDQEFDVVILVTQVLAKPAGVNIPPR
LALRRFFIPSNKSSSSPSSGSTFDSFHQRFGCLEAFKPNAKYTFLLADTREIVVQRGNVLPVFALSGPSLTFSEEVTAEILRY
LCRTCYSPQVNTFEPQILPFGEDFSATCVGNGDPLPQVMWYKDNYPVDIAANGRNVRVEIVQTSSHVREAILEIDNLVLLDNG
DYVCKASNPLGSTQSLLQLRVSTAGVSLTDQEIAMESRDLESCPADENHCFNDGECFAKKSNPLERRCKCKDGFMGDQCQFRT
SGIVFSTMSVGNANTIWLQTLLQFAFFGLFGLIVVGIILAFRRKDLRAKQKRKIMLGDEVLLKSQYIQVNTIEPPPPPINSPN
SSNLQVPRQKVNFLAPVMPQIPNYTRLDTGELNFNAYGDSRNANSSEESPTSNRPRLTLLQQPQRFGLTRGGLEPIEEQEVDA
SSNFLSSNHENPNFL*

```
>Prostheceraeus vitattus NRG-1
MRILPGQVLTLKCRAKGRPTPIVTWYKNGHQISSQEGHVDITMESKGRYSKLKITDVRKKDSGKYACHAESKNGLLNKWIKVQ
VSRREKIVCSQTFCLNGQCHVTTNKKKICECRYPYYGQFCEHEFIVQPPPPLTSSSSIATTPRPVPIRLRFHNLTTIRVPSTI
RGCDMAVEKSLLHAGISIATTVFVMLIIVLCYLCRLQRSKSFVWKARDGRYSRKSQSSQTELSFPNLLKSTPRKVLYLSTNGL
YDNAEATPSTPSLPGCMCPVSSDHLSMFCWPQTSSADTTVKSAAHTVKYRYSVPTDGFSNAENLVSTPLDKPPSQNSTLNRRN
KLNIESHSQSNDISRIKRWYYEHDKGDVSLCSPGHHTNSTEFINHSGSSDSQTRCTCNVQIDPSCDDQIPETDYLINLHNTEK
>Prostheceraeus vitattus NRG-2
MEIEQLREVEDYVGGLSTPQPKRLKVSHDDMVTVGEGHVLNLVCKAKGSPAPKIKWYRKDVAINATEGRVSVKTSNNGRLVKL
RISSIHPEDSGHYTCKVENTNGYIKKWVFLAVESKFAPTAKSETATHITSLAPTEEASPVIRRDSLLPSVDCSPASCINGECV
NLSEQRYFCKCKSQYIGLHCDKLRPSSLPSQNSSHTGLDPDCIGSHDCVRTVDRTLTYVGIGLAGIFLLLILGISWFMARQRR
KDWRVFRRKSKSSFSAKSGATGVAFALDSSRRASVRTVGVDVGTDAIFIEAEANGENANRPPRPGAALPRSVSFPADIEAPPD
PRPPSRHRFGTLPTSQAGPMPASQTRPSPSDFAYHPLRSMPSGESAAAMTLPTERLASPPSPRLVSVSHTLGRSRRDRHAEDA
EIWIKQQQASLGIAPPLGDVGNGGGGSGGSDSGVHSTSNPLYSDSTGSGSLNYVVPPPPIGKKTLPRRGHNSGKPPKEVNFKD
TAQTVTIAATAAHRHCLVAGGFEGPWERSFGGLPGFVTFANTQQERVCICPTHLPNGPVNVMAPPPPRLTTFAQHGSMNAASV
SNKTLPNHRPH
>Schmidtea mediterranea NRG
MFIVTFSIILRLNQVASMNNRHVNSDIMCSSDMMKDLYSRIHISPLIFLAKVLRKEKIPYSDSNWQLVTFVRVKRTLKDIFTP
NKLSSNHSLWIGPFTDQINNTLCMTLLKNNSDYIFFATQLSGSKNSFQSLYVPLYYSENIYQKINILLKQPIKPLVISRIPSY
NITVEGKIEVQCQAEGYPIPQFFWYKNSNKPILESKYKSRIFYRVEGIKSILHIDNVEKSDEANYTCHAENIYGYVDSSFKLE
VRETDESRRKCSGEPNFCIHGMCSKDKVSCLCKNGFYGRRCNYHHIPKLK
>Branchiostoma floridae NRG
METRIDRVKGALAAAHQRWQREVPLLSDKWLIVPAALILALVCFLVVTLKWIWVDHIFDSVELPTYGHGAVEMAAKGDETTGM
EGPSATVSSDGDGSSGDSSGEEDGRNQGGGVGTNIIPDVILPSVPPLLEPTPPETTTPTGTVSSTTPVKTPIFQLTTIPIFQL
TTIPTQKTFTGHGIPCRDQDAGYCLNGGTCTQLPAPGAGNKFEKRCTCTDDYFGNRCQYAASIRELQARLRRAEEIHQKRVLA
ITGICLALLVVGIMCGVAWYMSRKQRKKWCRKHLMANGEARGGDAEEPHEGEIMIPMTNLAGASRTEPKQDLADETSFRNGDS
RAPHDNPNGDVGRNQPMDDLQGGRRKRLNGVAKQALSKPSWQSTPNKDRSPRKEFAALPDEPSPIPEPSTPPRPLREHRPLPV
PDIQQFRQASRESSQEDLANESTPLRKGSSASSSGSSLQLDTSRPVRKPFITREPDDVDLSTKEFRPYDVTDGGDLTPEFTPT
NTPRIQRDRRDFPNNNPPYISTSRPSDEEDENDNFNATDPDFHARLRNVIENQEAVAV
>Homo sapiens NRG-1
MSERKEGRGKGKGKKKERGSGKKPESAAGSQSPALPPRLKEMKSQESAAGSKLVLRCETSSEYSSLRFKWFKNGNELNRKNKP
QNIKIQKKPGKSELRINKASLADSGEYMCKVISKLGNDSASANITIVESNEIITGMPASTEGAYVSSESPIRISVSTEGANTS
SSTSTSTTGTSHLVKCAEKEKTFCVNGGECFMVKDLSNPSRYLCKCQPGFTGARCTENVPMKVQNQEKAEELYQKRVLTITGI
CIALLVVGIMCVVAYCKTKKQRKKLHDRLRQSLRSERNNMMNIANGPHHPNPPPENVQLVNQYVSKNVISSEHIVEREAETSF
STSHYTSTAHHSTTVTQTPSHSWSNGHTESILSESHSVIVMSSVENSRHSSPTGGPRGRLNGTGGPRECNSFLRHARETPDSY
RDSPHSERYVSAMTTPARMSPVDFHTPSSPKSPPSEMSPPVSSMTVSMPSMAVSPFMEEERPLLLVTPPRLREKKFDHHPQQF
SSFHHNPAHDSNSLPASPLRIVEDEEYETTQEYEPAQEPVKKLANSRRAKRTKPNGHIANRLEVDSNTSSQSSNSESETEDER
VGEDTPFLGIQNPLAASLEATPAFRLADSRTNPAGRFSTQEEIQARLSSVIANQDPIAV
>Homo sapiens NRG-2
MRQVCCSALPPPPLEKGRCSSYSDSSSSSSERSSSSSSSSSESGSSSRSSSNNSSISRPAAPPEPRPQQQPQPRSPAARRAAA
RSRAAAAGGMRRDPAPGFSMLLFGVSLACYSPSLKSVQDQAYKAPVVVEGKVQGLVPAGGSSSNSTREPPASGRVALVKVLDK
WPLRSGGLQREQVISVGSCVPLERNQRYIFFLEPTEQPLVFKTAFAPLDTNGKNLKKEVGKILCTDCATRPKLKKMKSQTGQV
GEKQSLKCEAAAGNPQPSYRWFKDGKELNRSRDIRIKYGNGRKNSRLQFNKVKVEDAGEYVCEAENILGKDTVRGRLYVNSVS
TTLSSWSGHARKCNETAKSYCVNGGVCYYIEGINQLSCKCPNGFFGQRCLEKLPLRLYMPDPKQKAEELYQKRVLTITGICVA
LLVVGIVCVVAYCKTKKQRKQMHNHLRQNMCPAHQNRSLANGPSHPRLDPEEIQMADYISKNVPATDHVIRRETETTFSGSHS
CSPSHHCSTATPTSSHRHESHTWSLERSESLTSDSQSGIMLSSVGTSKCNSPACVEARARRAAAYNLEERRRATAPPYHDSVD
SLRDSPHSERYVSALTTPARLSPVDFHYSLATQVPTFEITSPNSAHAVSLPPAAPISYRLAEQQPLLRHPAPPGPGPGPGPGP
GPGADMQRSYDSYYYPAAGPGPRRGTCALGGSLGSLPASPFRIPEDDEYETTQECAPPPPPRPRARGASRRTSAGPRRWRRSR
LNGLAAQRARAARDSLSLSSGSGGGSASASDDDADDADGALAAESTPFLGLRGAHDALRSDSPPLCPAADSRTYYSLDSHSTR
ASSRHSRGPPPRAKQDSAPL
>Homo sapiens NRG-3
MSEGAAAASPPGAASAAAASAEEGTAAAAAAAAAGGGPDGGGEGAAEPPRELRCSDCIVWNRQQTWLCVVPLFIGFIGLGLSL
MLLKWIVVGSVKEYVPTDLVDSKGMGQDPFFLSKPSSFPKAMETTTTTTSTTSPATPSAGGAASSRTPNRISTRLTTITRAPT
RFPGHRVPIRASPRSTTARNTAAPATVPSTTAPFFSSSTLGSRPPVPGTPSTQAMPSWPTAAYATSSYLHDSTPSWTLSPFQD
AASSSSSSSSATTTTPETSTSPKFHTTTYSTERSEHFKPCRDKDLAYCLNDGECFVIETLTGSHKHCRCKEGYQGVRCDQFL
PKTDSILSDPTDHLGIEFMESEEVYQRQVLSISCIIFGIVIVGMFCAAFYFKSKKQAKQIQEQLKVPQNGKSYSLKASSTMAK
SENLVKSHVQLQNYSKVERHPVTALEKMMESSFVGPQSFPEVPSPDRGSQSVKHHRSLSSCCSPGQRSGMLHRNAFRRTPPSP
RSRLGGIVGPAYQQLEESRIPDQDTIPCQGIEVRKTISHLPIQLWCVERPLDLKYSSSGLKTQRNTSINMQLPSRETNPYFNS
LEQKDLVGYSSTRASSVPIIPSVGLEETCLQMPGISEVKSIKWCKNSYSADVVNVSIPVSDCLIAEQQEVKILLETVQEQIRI
LTDARRSEDYELASVETEDSASENTAFLPLSPTAKSEREAQFVLRNEIQRDSALTK
>Homo sapiens NRG-4
MPTDHEEPCGPSHKSFCLNGGLCYVIPTIPSPFCRCVENYTGARCEEVFLPGSSIQTKSNLFEAFVALAVLVTLIIGAFYFLC
RKGHFQRASSVQYDINLVETSSTSAHHSHEQH
>Saccoglossus kowalevskii NRG
MGDSNFYIDLRVSVVSIRLEHYIKGHALVPGPSQMSCPIASLNGTQPPTIKKRLRNKTVIDGNKFTLKCGIAGFPQPEISWTK
```

DGEVLTQGVRTKKSSSRVKVKKALQSKHAGNYTCIGNNWVDPPVSTSAIISVCSLECVHGEPNLKKCRCKCEDNWKGEKCDIE
RNSCHSKKCKHGSLNKESCKCICDDGYSGEFCNVTSIATTTVLAVTVPQCPKVTCGAHGALDVPKCECNCEYGWQGDLCETAT
PQHGIPCEVENFCLNQGKCYYLPDIGEKICHCPAPHTGPRCQYKDPFHVERTREDAEQLQAKRVLTVVGIFVALLVLIIICII
AWCKARTGKKKYQKRQKEKEKEKRKKEKEKEKLMTYPVNRINGDVPLNEDCYTPMQSMKNDPDDVQDELEREEETSFVCPLSH
NSGISSAYKASPDGCYGDGSPHHSLGSRHSNNSPYSSHRGSPQRVHQSSPRHNFRNHSYSPTHSPLQDVPLTIESINPSYANP
DHNNMYNQSGGENDVNAPLHNGMNLAIHETDSDLEDEMPRMPVAETEEEDQDSNLDDSYIYLCQQPDEPVPEHAFIRPMLDSF
EVGSNDFSLAEAEDGSVYTNDGDVTTNASESDSDSSSSASDSSSSSDDNTIPNLLLETLQQPYMPSDKESGEYAWDDEGLTVL
QHPEANQPRHCFANPHAVTNPSPEHRQRQQPQRTAPIANHYASNPLYARPQQSPNQHRNQEMVTDEEYLSRLKDMMENQKTLK
I
>Strongylocentrotus purpuratus NRG
MQCSWSCDPLEEPTDLKAYRSAIVVEGTLVGSTAFVERKTMKGQFPSRVSDIQLRGFASEDPCTGPVSSFSLNTTYLLFINET
SDEGVYKLMANPEESSRTSKRTVRKVVKDGDRPVMTKKLKSKTMGEGERLLLTCAGKGTPMPLIYWERDGRRLQDNDIPRLKI
KPSKRNVKLSIRNLDKTLHEGTYTCVLTNKVDPPIIASSILSICAIKCMHGERHPRKCRCDCDEGYSGDLCQIPDPTTPSPPT
TPTPCDLDCGSNGICAIKCMHGERHPRKCRCDCDEGYSGDLCQIPDPTTPSPPTTPAPCDLDCGTNGTLDDILCFCNCNPGFE
GQSCSEVLPTGRTPQPISPDNCTLNCGDHGNMVRGSCSCLCDEGYSGSTCASEFTSTVVDVGEESDNDISSSSSSDYQGIEQN
SMKVIITLTVIFISLLLVLLLVVFIYIKNKNYQEERRKRVKTSRNPLRPPSYNASHPLHQNTNNQAGITSRDTIQELEMEPRR
PSAPSAIMRVESEGSGHTTPSQRSHSVDVRYSSVPVQANPVVARTIHNGSARKISQASIHSNRSASSHRSNGSSPVSGKRRRL
GYEAVPMNEPADTIEEYHGDTAACEDASIIMLPREEVCSDGESECSSEPDDGVDMPNPQISAIEKHLAQPSEDYDDNVDRDFS
ADVVNANVHFSNPYAQDDSPSLSPRGHMTSNMSPHEVNLHENPFQDGLSLQGKSPTNKLYSNDGYYGNRYEDEIDRSSPPHAV
